# Yoga vs Cognitive Processing Therapy for Military Sexual Trauma–Related Posttraumatic Stress Disorder: A Randomized Clinical Trial

**DOI:** 10.1001/jamanetworkopen.2023.44862

**Published:** 2023-12-08

**Authors:** Belle Zaccari, Melinda Higgins, Terri N. Haywood, Meghna Patel, David Emerson, Kimberly Hubbard, Jennifer M. Loftis, Ursula A. Kelly

**Affiliations:** 1 Research and Development Service, Veterans Affairs Portland Health Care System, Portland, Oregon; 2 Department of Mental Health and Clinical Neurosciences, Veterans Affairs Portland Health Care System, Portland, Oregon; 3 Department of Psychiatry, Oregon Health & Science University, Portland; 4 Nell Hodgson Woodruff School of Nursing, Emory University, Atlanta, Georgia; 5 Atlanta VA Health Care System, Atlanta, Georgia; 6 Mental Health Service Line, Joseph Maxwell Cleland Atlanta VA Medical Center, Atlanta, Georgia; 7 Department of Psychiatry and Behavioral Sciences, Emory University School of Medicine, Atlanta, Georgia; 8 The Center for Trauma and Embodiment at Justice Resource Institute, Needham, Massachusetts; 9 Department of Behavioral Neuroscience, Oregon Health & Science University, Portland; 10 Nursing and Patient Care Services, Joseph Maxwell Cleland Atlanta VA Medical Center, Atlanta, Georgia

## Abstract

**Importance:**

First-line treatment for posttraumatic stress disorder (PTSD) in the US Department of Veterans Affairs (VA), ie, trauma-focused therapy, while effective, is limited by low treatment initiation, high dropout, and high treatment refraction.

**Objective:**

To evaluate the effectiveness of Trauma Center Trauma-Sensitive Yoga (TCTSY) vs first-line cognitive processing therapy (CPT) in women veterans with PTSD related to military sexual trauma (MST) and the hypothesis that PTSD outcomes would differ between the interventions.

**Design, Setting, and Participants:**

This multisite randomized clinical trial was conducted from December 1, 2015, to April 30, 2022, within 2 VA health care systems located in the southeast and northwest. Women veterans aged 22 to 71 years with MST-related PTSD were enrolled and randomized to TCTSY or CPT.

**Interventions:**

The TCTSY intervention (Hatha-style yoga focusing on interoception and empowerment) consisted of 10 weekly, 60-minute group sessions, and the CPT intervention (cognitive-based therapy targeting modification of negative posttraumatic thoughts) consisted of 12 weekly, 90-minute group sessions.

**Main Outcome and Measures:**

Sociodemographic data were collected via self-report survey. The primary outcome, PTSD symptom severity, was assessed using the Clinician-Administered PTSD Scale for *DSM-5* (CAPS-5) and PTSD Checklist for *DSM-5* (PCL-5). Assessments were conducted at baseline, midintervention, 2 weeks post intervention, and 3 months post intervention.

**Results:**

Of 200 women veterans who consented to participate, the intent-to-treat sample comprised 131 participants (mean [SD] age, 48.2 [11.2] years), with 72 randomized to TCTSY and 59 randomized to CPT. Treatment was completed by 47 participants (65.3%) in the TCTSY group and 27 (45.8%) in the CPT group, a 42.6% higher treatment completion rate in the TCTSY group (*P* = .03). Both treatment groups improved over time on the CAPS-5 (mean [SD] scores at baseline: 36.73 [8.79] for TCTSY and 35.52 [7.49] for CPT; mean [SD] scores at 3 months: 24.03 [11.55] for TCTSY and 22.15 [13.56]) and the PCL-5 (mean [SD] scores at baseline: 49.62 [12.19] for TCTSY and 48.69 [13.62] for CPT; mean [SD] scores at 3 months: 36.97 [17.74] for TCTSY and 31.76 [12.47]) (*P* < .001 for time effects). None of the group effects or group-by-time effects were significant. Equivalence analyses of change scores were not significantly different between the TCTSY and CPT groups, and the two one-sided test intervals fell within the equivalence bounds of plus or minus 10 for CAPS-5 for all follow-up time points.

**Conclusions and Relevance:**

In this comparative effectiveness randomized clinical trial, TCTSY was equivalent to CPT in reducing PTSD symptom severity, with both groups improving significantly. The higher treatment completion rate for TCTSY indicates its higher acceptability as an effective and acceptable PTSD treatment for women veterans with PTSD related to MST that could address current VA PTSD treatment limitations.

**Trial Registration:**

ClinicalTrials.gov Identifier: NCT02640690

## Key Points

**Question:**

What is the effectiveness of Trauma Center Trauma-Sensitive Yoga (TCTSY) compared with first-line cognitive processing therapy (CPT) for posttraumatic stress disorder (PTSD) related to military sexual trauma in women veterans?

**Findings:**

In this randomized clinical trial of 131 women veterans with PTSD who use US Department of Veterans Affairs health care, TCTSY had large within-group effect sizes, equivalent effectiveness to CPT, and a 42.6% higher treatment completion rate than CPT.

**Meaning:**

The findings demonstrate that TCTSY is a cost-effective means to expand Veterans Affairs PTSD treatment options; increase access to acceptable, patient-driven, and effective PTSD treatment for women veterans; and provide a treatment option that could improve associated symptoms (eg, depression, anxiety).

## Teaser

This randomized clinical trial evaluated the effectiveness of trauma-sensitive yoga vs cognitive processing therapy for women veterans with military sexual trauma–related posttraumatic stress disorder (PTSD).

## Introduction

Posttraumatic stress disorder (PTSD) is prevalent and harmful to veterans’ physical and mental health, functioning, and well-being and has tremendous societal costs.^1^ The harmful consequences of PTSD for women veterans and associated economic costs to the Veterans Health Administration are increasing exponentially. One in 5 women patients in the Veterans Health Administration had PTSD in 2015, a 300% increase in prevalence from 2000.^2^ Military sexual trauma (MST) also is prevalent (38%) among women veterans and is their leading cause of military service–related PTSD.^3,4^ Military sexual trauma is defined by the US Department of Veterans Affairs (VA) as threatening sexual harassment or sexual assault while serving in the military.^5^ It is associated with numerous physical and psychiatric conditions, increased mortality due to suicide, and deficits in social determinants of health (eg, housing instability).^6,7,8^ Yet, many veterans do not engage in VA health care provided at no cost for MST-related conditions.^9,10^ Some MST survivors perceive that US military institutions have failed to act in ways that prevent harm or have created environments that can lead to or fail to ameliorate harm (ie, institutional betrayal).^11^ Institutional betrayal related to MST has been associated with more severe depression and PTSD; suicidal, self-directed violence^12,13^; and lower willingness to use VA health care.^13^ This reticence to seek PTSD treatment in the VA is compounded by the lack of PTSD treatments that are both effective and acceptable to veterans, particularly those who experienced MST.

First-line, evidence-based treatments (EBTs) for PTSD offered by the VA are trauma-focused psychotherapies, eg, prolonged exposure and cognitive processing therapy (CPT); however, these therapies lack acceptability and have high treatment dropout and incomplete effect, with more than one-half of treatment completers retaining their PTSD diagnosis.^14,15,16^ Veterans’ preference for and use of yoga for PTSD treatment have grown significantly and are supported by VA expansion of access to complementary and integrative health (CIH) modalities.^17^ Evidence of the effectiveness of yoga to treat PTSD is growing.^18,19,20,21,22^

In this multisite, randomized clinical trial (RCT), we examined the effectiveness of Trauma Center Trauma-Sensitive Yoga (TCTSY), a Hatha-style yoga intervention designed for women who experienced childhood sexual trauma. In contrast to cognitively based EBTs, TCTSY is a body-based intervention that focuses on reducing stress reactions of the body by cultivating interoception.^23,24^ This study is the first fully powered RCT to compare a yoga modality with a gold-standard PTSD treatment (CPT) and builds on our prior work.^20,25^ The primary aim of this study was to evaluate the effectiveness of TCTSY compared with CPT on outcomes of PTSD, chronic pain, and insomnia. We hypothesized that there would be a difference in PTSD outcomes between the interventions. Previous interim results demonstrated significant reductions in PTSD severity with large effect sizes for both TCTSY and CPT without significant differences between groups.^22^ Here, we present the final PTSD outcomes, including additional post hoc equivalence analyses.

## Methods

### Setting, Study Design, and Participants

For this RCT, we recruited participants from VA PTSD and other clinics from December 5, 2015, through June 22, 2020. In March 2020, due to the COVID-19 pandemic, we adapted enrollment, data collection, and intervention delivery from in-person to virtual methods.^26^ The study protocol including these changes is provided in Supplement 1. The data analysis was conducted from December 1, 2015, to April 30, 2022. This RCT was approved by the Emory University institutional review board and relevant VA institutional review boards and research committees. All participants gave written informed consent. The Consolidated Standards of Reporting Trials (CONSORT) reporting guideline was followed.

Participants were randomized to TCTSY or CPT using randomization sequences initially in groups of 24, then in 16 to 20 due to lower-than-expected enrollment (details reported elsewhere^22^). Group allocation was revealed following the baseline assessment. Additional assessments were conducted at midintervention, 2 weeks post intervention, and 3 months post intervention.

**Figure 1.**
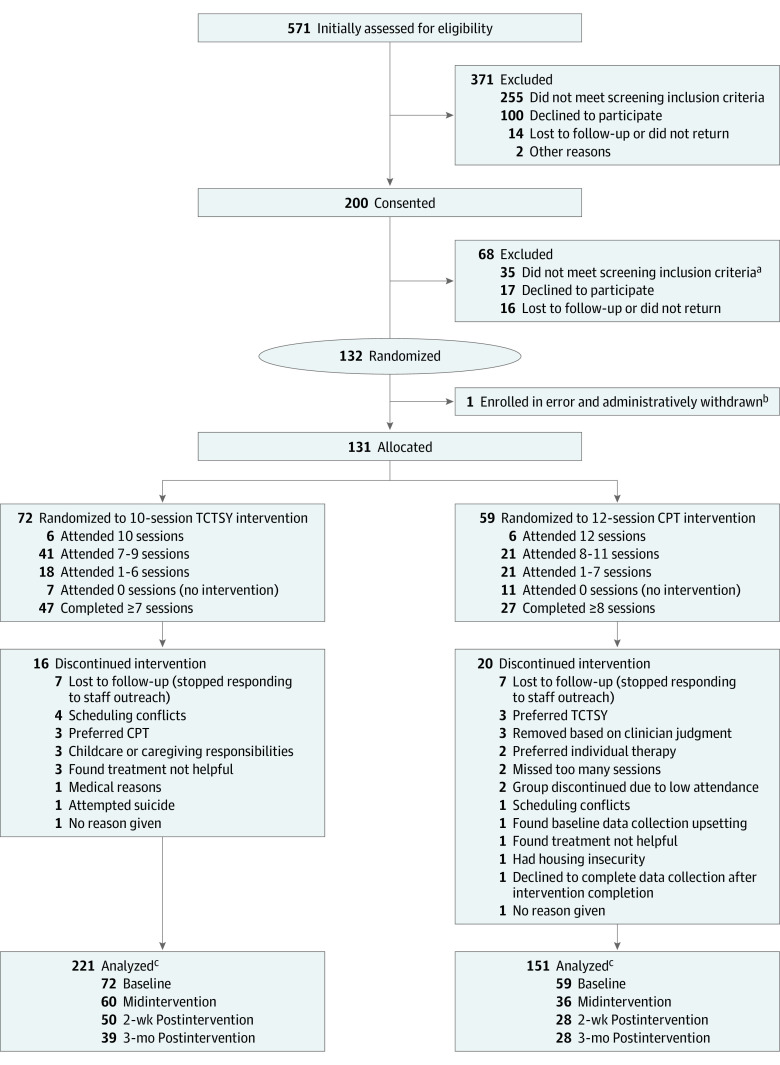
CONSORT Chart for Combined Sites Postrandomization attrition for the Trauma Center Trauma-Sensitive Yoga (TCTSY) intervention was 23 of 72 (31.9%) and for the cognitive processing therapy (CPT) intervention, 27 of 59 (45.8%). ^a^After consent, full assessments were completed to confirm eligibility. ^b^Participant was consented in error; did not meet eligibility criteria for posttraumatic stress disorder related to military sexual trauma. ^c^Analytic sample sizes vary for CAPS-5 and PCL-5 due to participants completing PCL-5 but not CAPS-5

Participants were women veterans aged 22 to 71 years at southeast (site 1) and northwest (site 2) VA health care systems. Sociodemographic data were collected via self-report survey. We included race and ethnicity as a variable because the existing literature on yoga as a treatment for PTSD is largely conducted with White populations, lacking diversity. We also included it because the racial and ethnic profile of the 2 study sites were substantially different. Inclusion criteria were (1) enrolled in VA care, (2) experienced MST, (3) had a current PTSD diagnosis with MST as the index trauma, and (4) had insomnia. Exclusion criteria included current EBT or yoga practice; suicidal ideation with intent or plan; moderate or severe traumatic brain injury or cognitive impairment; and moderate or severe substance abuse disorder, psychosis, or mania. Of the 200 women who consented to participate, 132 were eligible and randomized; the final intent-to-treat (ITT) sample was 131 participants (Figure 1; eFigures 1 and 2 in Supplement 2).

### Outcome Measures

The primary outcome of PTSD symptom severity was assessed using the Clinician-Administered PTSD Scale for *DSM-5* (CAPS-5) and PTSD Checklist for *DSM-5* (PCL-5).^27,28,29^ The CAPS-5 measures 20 PTSD symptoms on a Likert scale (0-4) that are summed for a total severity score of 0 to 80. The CAPS-5 interviews were audio recorded; data quality was maintained via review of 10% or more of recordings and ongoing CAPS-5 supervision by study investigators (B.Z. and M.P.). The PCL-5 self-report measure uses a Likert scale (0-4) to quantify the extent to which the respondent is bothered by 20 PTSD-related symptoms that are summed for a total severity score of 0 to 80. Additional primary outcomes were chronic pain and insomnia. Given the clinical complexity of these symptoms in the context of PTSD and the multiple moderators we considered (eg, obstructive sleep apnea), pain and insomnia outcomes will be reported elsewhere along with secondary outcomes.

### Interventions

Each intervention was delivered by 2 interventionists using established manualized protocols. Site 1 conducted sessions in person for 9 cohorts, except for the second half of the ninth cohort, which was conducted virtually, as was the single cohort at site 2. We provided a no-cost intervention crossover option for participants in the first 8 cohorts.

### Trauma Center Trauma-Sensitive Yoga

Trauma Center Trauma-Sensitive Yoga consisted of 10 weekly, 60-minute group sessions (10 hours of contact time). The treatment was created for survivors of sexual trauma; its foundations include trauma theory, attachment theory, and neuroscience.^30,31,32^ The core components of TCTSY are interoception, invitational language, choice making, noncoercion, and shared authentic experience. The TCTSY treatment centers around power sharing between facilitator and participant, emphasizes identification and clarification of body sensations as experienced within the physical yoga forms, and facilitates participants’ experiences of choice and noncoercion and taking effective action. The treatment was provided by TCTSY-certified facilitators who held registered yoga teacher credentials from the Yoga Alliance of at least 200 training hours.

### Cognitive Processing Therapy

Cognitive processing therapy consisted of 12 weekly, 90-minute group sessions (18 hours of contact time). The therapy involves modification of negative posttraumatic cognitions^33,34^; it helps patients identify the impact of traumatic events, including their sense-making of why the trauma occurred and how it has changed their views about themselves, others, and the world. Clinicians use Socratic dialogue and progressive worksheets that help the patient learn how to challenge their trauma-related stuck points (ie, unrealistic beliefs) and generate more balanced, realistic thoughts. The therapy was provided by VA CPT-certified licensed clinicians.

### Treatment Supervision and Fidelity Monitoring

The TCTSY facilitators completed standardized session and fidelity notes immediately after each session. Supervision was provided by a cofounder of TCTSY (D.E.) via weekly meetings with the facilitators. These meetings included fidelity feedback between the co-facilitators’ (D.E. and U.A.K.) review of fidelity notes with the TCTSY facilitators, debriefing of each session, and ongoing supervision. One TCTSY facilitator conducted sessions in all 9 cohorts at site 1 and supervised the TCTSY facilitators at site 2 with D.E., enhancing consistency in intervention delivery.

For CPT, clinicians maintained fidelity to the 2014 protocolized treatment manual,^34^ using the VA CPT SharePoint site for resources, case consultation, and a community practice call. Supervision was provided by a co-investigator (M.P.), a VA CPT trainer. Seven of the 9 CPT cohorts at site 1 were conducted by the same 2 clinicians, enhancing consistency in intervention delivery.

A data and safety monitoring board was established with the addition of site 2. There were no clinically significant adverse events reported throughout the trial.

### Data Plan

The study was proposed to have a final sample size of 104 participants (52 per group) after expected levels of 50% attrition from 208. This sample size was powered at 80% to detect moderate effect sizes for differences between the groups (Cohen *d* = 0.555 for differences in continuous outcomes and Cohen ω = 0.274 for differences in proportions), as well as moderate to large effect sizes for differences between the groups over time (group-by-time interaction effect size *f* = 0.28).^35^ Power analyses were completed using Power Analysis and Sample Size Software, version 13.0.8.^36^ The final sample size was 103 participants, close to the planned sample size (Figure 1).

### Statistical Analysis

Prior to analysis, all data were reviewed for completeness, missingness, and normality assumptions. Multilevel linear models with random effects for participants were used to compare the longitudinal outcomes over the 4 time points between the 2 groups for the CAPS-5 severity and PCL-5 scores, where group and time were fixed effects.^37^ For testing the main group and time effects and the group-by-time interaction effects, we used post hoc tests to compare changes from baseline to the 3 follow-up time points and differences between the 2 groups at each time point using Šidák pairwise error rate adjustment.^37^ We computed point estimates (means, SDs, 95% CIs, and effect sizes [Cohen *d*]).^35^ The study was designed to compare the gold standard of CPT with TCTSY using inequality hypothesis testing and an ITT approach, including data from all participants who had baseline outcome data, regardless of further study engagement. Additional analyses were performed for treatment completers, ie, an adequate dose per protocol (PP). Treatment completion definitions were 7 or more out of 10 for TCTSY sessions and 8 or more out of 12 for CPT sessions, consistent with the VA literature (eFigure 3 in Supplement 2).^38,39,40^

While the study was designed to test for group differences between CPT and TCTSY, given the lack of significant differences between the 2 treatments, we followed up our analyses with tests of equivalence to make specific estimates about the effect size we deemed worthwhile to examine.^41^ The equivalence tests compared the means between the 2 groups using two one-sided tests (TOSTs) via the TOSTER, version 0.4.0 package in R (eMethods 1 in Supplement 2).^41,42^ Participants’ change scores from baseline to each follow-up time point were used to perform TOSTs to test for equivalence between the 2 groups relative to the smallest effect size of interest. A smallest effect size of interest margin of 10 was used for the change score differences on the CAPS-5 and PCL-5 between the treatment groups, similar to previously published approaches for a clinically meaningful difference of plus or minus 10 for the CAPS-5 and PCL-5.^43,44^

Four clinically relevant end points (CAPS-5) are reported at each time point for the percentage of participants who (1) still met the criteria for a PTSD diagnosis, (2) achieved a clinical response (reduction of CAPS-5 severity scores of ≥10 points), (3) had a loss of diagnosis (≥10-point improvement, no PTSD diagnosis, and CAPS-5 severity score <25), and (4) achieved remission (loss of diagnosis plus CAPS-5 score <12).^44^ Statistical analyses were completed using SPSS, version 27.0.0.0^45^ and R, version 4.1.2^46^ software. A 2-sided *P* < .05 was considered significant. Given the COVID-19–related protocol adaptions, sensitivity analyses were conducted while running all the analyses described above with and without site 2 (virtual) and with and without the last cohort of site 1 (hybrid in person and virtual) (eMethods 2 in Supplement 2).

## Results

The ITT sample (n = 131) had a mean (SD) age of 48.2 (11.2) years; 95 (72.6%) self-identified as African American or Black; 11 (8.4%) as American Indian or Alaska Native, Asian, multiracial, or other race; and 25 (19.1%) as White. Detailed demographic information and clinical characteristics are provided in Table 1 (eTable 1 in Supplement 2 provides similar data and trauma exposure by study site). Participant dropout before the first intervention session was nearly double for CPT (11 [18.6%]) than for TCTSY (7 [9.7%]). Attrition post randomization through final intervention sessions was higher for CPT (27 of 59 [45.8%]) than for TCTSY (23 of 72 [31.9%]) (Figure 1). Nearly two-thirds (47 [65.3%]) of the TCTSY group completed treatment compared with less than one-half (27 [45.8%]) of the CPT group; ie, TCTSY had a 42.6% higher treatment completion rate than CPT, a significant difference (*P* = .03).

**Table 1.** Demographic and Clinical Characteristics

Characteristic	No. (%)		
	TCTSY (n = 72)	CPT (n = 59)	Total (N = 131)
Age, mean (SD) [range], y	48.2 (11.0) [26-70]	48.3 (11.6) [22-71]	48.2 (11.2) [22-71]
Race			
African American or Black	51 (70.8)	44 (74.6)	95 (72.6)
American Indian or Alaska Native	0	1 (1.7)	1 (0.8)
Asian	1 (1.4)	0	1 (0.8)
White	14 (19.4)	11 (18.6)	25 (19.1)
Multiracial^a^	4 (5.6)	3 (5.1)	7 (5.3)
Other^b^	2 (2.8)	0	2 (1.5)
Relationship status			
Nonpartnered	43 (57.7)	44 (74.6)	87 (66.4)
Married or partnered	29 (40.3)	15 (25.4)	44 (33.6)
Education level			
Less than college	27 (37.5)	26 (44.1)	53 (40.5)
College degree or more	45 (62.5)	33 (55.9)	78 (59.5)
Monthly income,^c^ $			
<2000	25 (35.2)	24 (40.7)	49 (37.7)
≥2000	46 (64.8)	35 (59.3)	81 (62.3)
Employment status			
Less than full time	47 (65.3)	45 (76.3)	92 (70.2)
Full time	25 (34.7)	14 (23.7)	39 (29.8)
BDI total score^c^			
Mean (SD)	28.0 (9.8)	29.0 (11.8)	28.5 (10.7)
Range	5-55	8-58	5-58
BDI total categories^c^			
Minimal (0-13)	4 (5.8)	6 (10.5)	10 (7.9)
Mild (14-19)	10 (14.5)	6 (10.5)	16 (12.7)
Moderate (20-28)	22 (31.9)	17 (29.8)	39 (31.0)
Severe (29-63)	33 (47.8)	28 (49.1)	61 (48.4)
MINI			
Suicidality (past month)			
No	50 (69.4)	33 (55.9)	83 (63.4)
Yes^d^	22 (30.6)	26 (44.1)	48 (36.6)
Suicidality (lifetime attempt)			
No	56 (77.8)	42 (71.2)	98 (74.8)
Yes	16 (22.2)	17 (28.8)	33 (25.2)
Suicidality rating^d^			
Low (1-8)	12 (54.5)	12 (46.2)	24 (50.0)
Moderate (9-16)	2 (9.1)	4 (15.4)	6 (12.5)
High (≥17)	8 (36.4)	10 (38.5)	18 (37.5)

Abbreviations: BDI, Beck Depression Inventory; CPT, cognitive processing therapy; MINI, Mini International Neuropsychiatric Interview; TCTSY, Trauma Center Trauma-Sensitive Yoga.

^a^ Four women self-reported as also Hispanic or Latino.

^b^ For the 2 women who self-reported other, 1 reported as being “African American, Caucasian” and 1 as “West African.”

^c^ Missing income for 1 participant; missing BDI total score for 5 participants.

^d^ Suicidality rating only completed for the 48 subjects who said yes for any lifetime suicide.

Both groups improved over time in the ITT analysis for CAPS-5 (mean [SD] scores at baseline: 36.73 [8.79] for TCTSY and 35.52 [7.49] for CPT; mean [SD] scores at 3 months: 24.03 [11.55] for TCTSY and 22.15 [13.56]) and the PCL-5 (mean [SD] scores at baseline: 49.62 [12.19] for TCTSY and 48.69 [13.62] for CPT; mean [SD] scores at 3 months: 36.97 [17.74] for TCTSY and 31.76 [12.47]; *P* < .001); these improvements were also seen in the PP analysis (Table 2; eTables 2 and 3 and eFigures 4 and 5 in Supplement 2). None of the group effects or group-by-time effects were statistically significant. None of the post hoc group comparison tests were significant, and all effect sizes for group differences were small (Cohen *d* < 0.367) (Table 2), indicating similar results in both groups. Nearly all within-group improvements in CAPS-5 and PCL-5 outcomes had moderate to large effect sizes for TCTSY and CPT (eTable 4 in Supplement 2).

**Table 2.** Posttraumatic Stress Disorder (PTSD) Outcomes Intent-to-Treat and Per-Protocol Group Difference Effect Sizes

	Baseline		Midpoint		2 wk Post intervention		3 mo Post intervention	
	No.	Mean (SD)	No.	Mean (SD)	No.	Mean (SD)	No.	Mean (SD)
**CAPS-5 (intent to treat)** ^a^								
TCTSY	71	36.73 (8.79)	59	26.32 (10.09)	50	23.24 (11.68)	39	24.03 (11.55)
CPT	58	35.52 (7.49)	34	28.97 (12.20)	26	27.77 (14.77)	27	22.15 (13.56)
Effect size		0.148		−0.243		−0.354		0.151
**CAPS-5 (per protocol)** ^b^								
TCTSY	46	35.13 (8.36)	45	26.56 (10.04)	45	23.51 (11.60)	36	24.08 (11.49)
CPT	27	37.22 (8.51)	24	30.58 (12.54)	20	28.05 (15.03)	21	22.00 (13.46)
Effect size		−0.249		−0.367		−0.357		0.170
**PCL-5 (intent to treat)** ^c^								
TCTSY	71	49.62 (12.19)	59	42.49 (14.25)	50	38.68 (15.72)	39	36.97 (17.74)
CPT	59	48.69 (13.62)	35	45.83 (15.77)	27	39.00 (17.65)	27	33.59 (15.51)
Effect size		0.072		−0.225		−0.019		0.200
**PCL-5 (per protocol)** ^d^								
TCTSY	47	47.02 (12.07)	45	41.69 (15.00)	45	39.04 (15.71)	36	36.22 (17.36)
CPT	27	47.48 (15.53)	25	44.04 (15.36)	21	37.52 (16.76)	21	31.76 (12.47)
Effect size		−0.034		−0.155		0.095		0.283

Abbreviations: CAPS-5, Clinician-Administered PTSD Scale for *DSM-5*; CPT, cognitive processing therapy; PCL-5, PTSD Checklist for *DSM-5*; TCTSY, Trauma Center Trauma-Sensitive Yoga.

^a^ Group: *F*_(1,141.6)_ = 0.00, *P* = .98; time: *F*_(3,258.6)_ = 48.13, *P* < .001; group by time: *F*_(3,258.6)_ = 1.87, *P* = .14.

^b^ Group: *F*_(1,73.6)_ = 1.00, *P* = .32; time: *F*_(3,189.8)_ = 31.77, *P* < .001; group by time: *F*_(3,189.8)_ = 1.61, *P* = .19.

^c^ Group: *F*_(1,139.2)_ = 0.17, *P* = .68; time: *F*_(3,257.2)_ = 23.30, *P* < .001; group by time: *F*_(3,257.2)_ = 1.55, *P* = .20.

^d^ Group: *F*_(1,74.1)_ = 0.10, *P* = .76; time: *F*_(3,192.5)_ = 16.71, *P* < .001; group by time: *F*_(3,192.5)_ = 1.27, *P* = .29.

**Figure 2.**
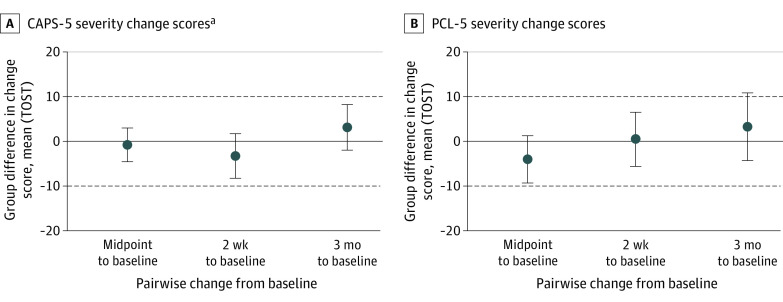
Equivalence Test Limits for Differences Between Groups for Clinician-Administered Posttraumatic Stress Disorder (PTSD) Scale for *DSM-5* (CAPS-5) Severity and PTSD Checklist for *DSM-5* (PCL-5) Change Scores Mean difference less than 0 indicates better improvement in 1 intervention over the other. Dots indicate the mean group difference in CAPS-5 change scores; bars, upper and lower bounds of the two one-sided test (TOST) intervals, dashed lines, plus or minus 10 equivalence bounds.

**Figure 3.**
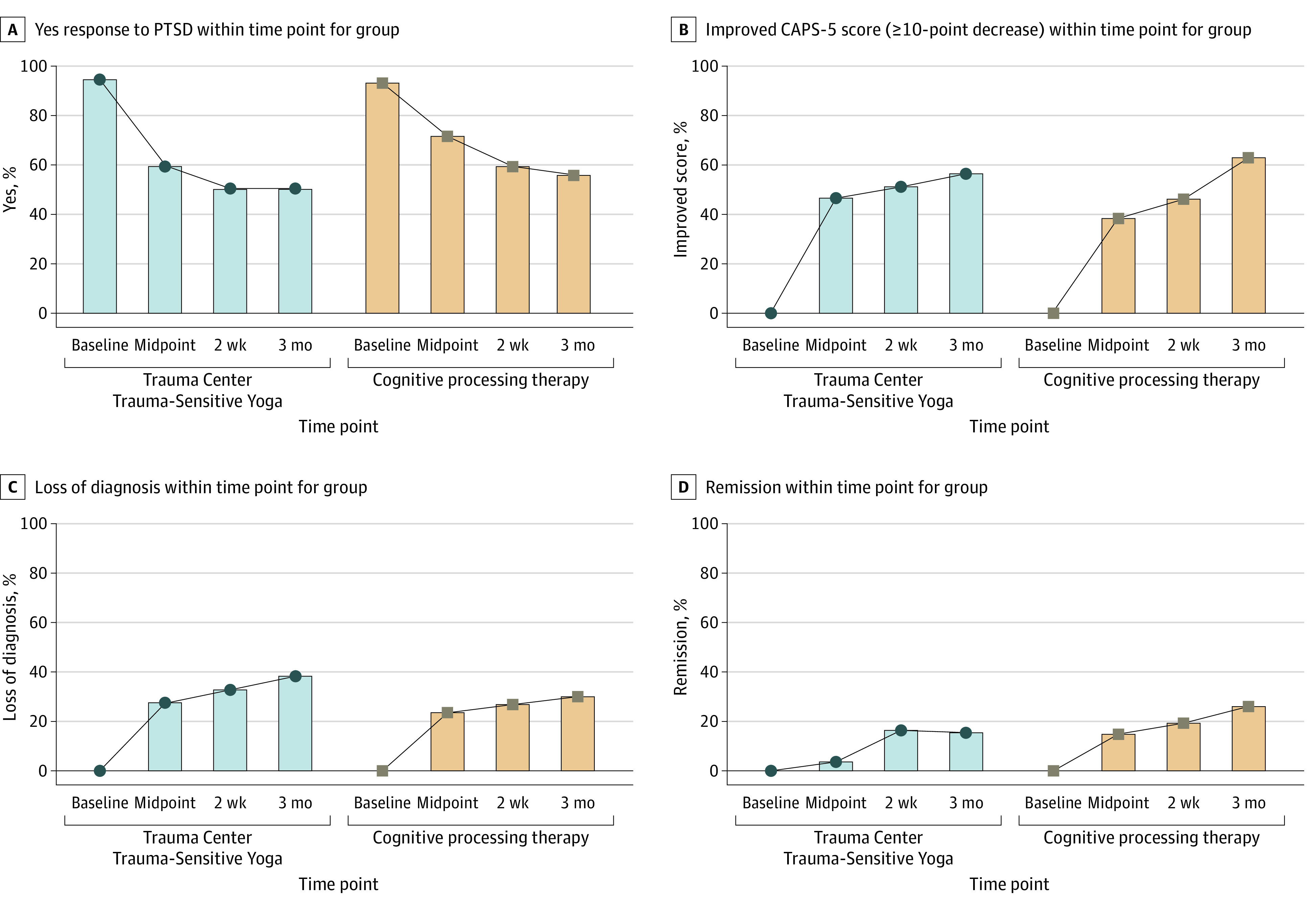
Clinical Posttraumatic Stress Disorder (PTSD) Diagnostic Changes CAPS-5 indicates Clinician-Administered PTSD Scale for *DSM-5*.

Subsequent analyses indicated treatment effectiveness equivalence between TCTSY and CPT on the CAPS-5 and PCL-5 (eTable 5 in Supplement 2). None of the change scores were significantly different between the TCTSY and CPT groups, and all TOST intervals fell within the equivalence bounds of plus or minus 10 for CAPS-5 and PCL-5 for every follow-up time point except PCL-5 changes from baseline to 3 months, which fell slightly outside the equivalence bounds (Figure 2; eTables 5 and 6 in Supplement 2). The average mean differences in change scores for both CAPS-5 and PCL-5 at each follow-up were less than 5 (or greater than −5), indicating small differences between TCTSY and CPT improvements (ie, treatment equivalence) in both the ITT and PP analyses. Analysis of clinically relevant outcomes at the study end point showed no significant differences between TCTSY and CPT for the presence of a PTSD diagnosis, a clinical response, loss of diagnosis, or remission (Figure 3; eTable 7 in Supplement 2).

## Discussion

The significant effectiveness of TCTSY for PTSD and the equivalence of TCTSY to CPT in improving PTSD outcomes confirm and extend our earlier findings.^22^ Notably, TCTSY had higher treatment initiation, retention, and completion than CPT. The TCTSY group had more robust symptom improvement early on, which may have contributed to higher retention and treatment completion than in the CPT group (eFigures 4 and 5 and eTables 2 and 3 in Supplement 2). However, the fraction of CPT participants who completed treatment continued to improve from 2 weeks to 3 months after treatment, while improvement leveled off for TCTSY participants. It is important to note the differences in total treatment time (18 hours [CPT] vs 10 hours [TCTSY]). While the TCTSY completion rate was higher at end point, the majority of participants who did not complete CPT dropped out within the first few sessions, not as a result of a longer time frame for treatment.

The TCTSY within-group effect sizes in this study (CAPS-5 ITT Cohen *d* = −0.90 to −0.93) compare favorably to those reported in a systematic review of 7 RCTs of yoga for PTSD, which indicated a postintervention average weighted Cohen *d* of 0.48 for PTSD symptoms.^21^ The TCTSY and CPT groups also showed similar declines in all the clinically relevant end points described by Schnurr et al,^44^ ie, continued PTSD diagnosis, clinical response, loss of diagnosis, and remission.

The TCTSY treatment completion rate was 42.6% higher than the CPT rate and is perhaps the most valuable finding, given the limited engagement in and completion of EBTs, particularly among women veterans.^13,47,48^ This higher completion rate indicates that TCTSY is more acceptable than CPT, which has higher treatment completion than prolonged exposure.^44^ This finding is critical given the personal burden and consequences of untreated or unresolved PTSD in women veterans,^49,50^ as well as the health care costs of PTSD. The total cost of PTSD for military populations in 2018 was $42.7 billion, driven by disability and direct health care costs.^1^

Yoga modulates the stress response, regulates the sympathetic nervous system, and activates the parasympathetic nervous system.^51^ Theoretically, via these mechanisms, in addition to improving autonomic nervous system dysregulation in PTSD,^52^ yoga improves cross-cutting symptoms of stress, anxiety, depression, and pain.^53,54^ As such, yoga may be appealing to individuals who are experiencing these and other stress-related symptoms in the absence of an established PTSD diagnosis or awareness that their symptoms represent PTSD.^55^

Treatment for PTSD that uses different theoretical frameworks and mechanisms of action is needed for veterans for whom existing EBTs are unacceptable or ineffective. Our premise was that sexual trauma is experienced by the body first and primarily. As such, an embodied treatment approach provides a direct trajectory for healing as opposed to the indirect pathway of cognitively based EBTs. However, yoga modalities in studies of yoga for PTSD have varied widely. The TCTSY modality was designed for women with complex trauma (childhood sexual abuse) and chronic, treatment-resistant PTSD, features shared by the clinical population of VA-using women veterans with PTSD related to MST. This design of TCTSY might explain the more robust CAPS-5 and PCL-5 change scores in the TCTSY group compared with those in a study by Davis et al^18^ of a Hatha yoga–based holistic yoga program for PTSD. The implication is that the mechanisms of yoga may differ across yoga types; thus, it is important to match the theorized mechanism of a yoga intervention to the target phenomena and outcomes, ie, TCTSY for PTSD related to sexual trauma.

Treatment of PTSD that is acceptable and effective is medical care to which all veterans are entitled and that is currently lacking. The robust equivalence results combined with the notably higher TCTSY completion rate indicate that TCTSY is such a treatment. While no single PTSD treatment will be acceptable and effective for everyone, TCTSY may help women veterans overcome resistance to seeking care at the VA and provide effective PTSD treatment, an acceptable first option for PTSD treatment, or an option for those who withdraw from or do not improve sufficiently with EBTs. Additionally, TCTSY is more widely available in community settings, such as wellness programs, yoga programs, and independent TCTSY facilities, than CPT, providing improved access to treatment for a broader population of women veterans and civilian women with PTSD related to sexual trauma.

### Limitations

This study has several limitations. First, the study end point of 3 months post intervention precluded determination of even longer-term sustained effects of the interventions. Second, the CAPS-5 assessors were not blinded, introducing a risk of bias. Third, participants may have participated in unreported CIH modalities or psychotherapy that could have confounded the results. Finally, the final cohort at site 1 and the only cohort at site 2 were conducted during the COVID-19 pandemic, introducing additional stressors, isolation, restricted access to health care, and a pivot of study procedures to virtual delivery, all potential confounders of study data during that time.

## Conclusions

The costs of untreated or incompletely treated PTSD to women veterans and their families, the VA, and society are sizable. The VA is facing exponential increases in the number of women enrolled and with PTSD. Given the limitations of first-line EBTs in the VA, new acceptable and effective PTSD treatments could reduce the economic costs of PTSD and the burden on women veterans and their families. The findings of this RCT demonstrate that TCTSY is an effective and acceptable treatment to be added to current EBTs. Policy and practice changes to include TCTSY could be a cost-effective means to expand PTSD treatment options; provide a treatment that could improve associated symptoms, such as depression and anxiety; potentially mitigate PTSD-related physical symptoms and comorbidities; address veterans’ preferences for CIH therapies; and increase access to acceptable, patient-driven, and effective PTSD treatment.

## Notes

Supplement 1.
Trial Protocol

Supplement 2. **eFigure 1.** CONSORT Chart for Primary Site (Site 1) **eFigure 2.** CONSORT Chart for Second Site (Site 2) **eFigure 3.** Number of Sessions Attended by Group **eMethods 1.** Equivalence Tests: Data Analysis Details **eMethods 2.** COVID Sensitivity Analysis **eTable 1.** Demographics, Clinical Characteristics, and Trauma Exposure by Study Site **eTable 2.** CAPS-5 Intent-to-Treat and Per-Protocol Group Differences Detailed **eTable 3.** PCL-5 Intent-to-Treat and Per-Protocol Group Differences Detailed **eFigure 4.** PTSD Severity Scores Over Time **eFigure 5.** CAPS-5 and PCL-5 Severity Scores **eTable 4.** CAPS-5 and PCL-5 Intent-to-Treat (ITT) and Per-Protocol (PP) Within-Group Differences From Baseline **eTable 5.** Tests of Equivalence: CAPS-5 Severity and PCL-5 Change Scores From Baseline **eTable 6.** CAPS-5 Severity and PCL-5 Change Scores From Baseline by Time and Group and Group Equivalence Tests (ITT and PP) **eTable 7.** Clinical PTSD Diagnostic Changes

Supplement 3.
Data Sharing Statement

## References

[1] Davis LL, Schein J, Cloutier M, . The economic burden of posttraumatic stress disorder in the United States from a societal perspective. J Clin Psychiatry. 2022;83(3):21m14116. doi:10.4088/JCP.21m14116 35485933

[2] Frayne SMPC, Saechao F, Friedman SA, . Sourcebook: Women Veterans in the Veterans Health Administration: Volume 4: Longitudinal Trends in Sociodemographics, Utilization, Health Profile, and Geographic Distribution. Veterans Health Administration, Dept of Veterans Affairs; 2018.

[3] Wilson LC. The prevalence of military sexual trauma: a meta-analysis. Trauma Violence Abuse. 2018;19(5):584-597. doi:10.1177/1524838016683459 30415636

[4] Department of Defense Inspector General. Report on Sexual Harassment and Assault at the U. S. Air Force Academy. Dept of Defense; 2005.

[5] Counseling and Treatment for Sexual Trauma. 38 USC §1720D (2014).

[6] Nichter B, Holliday R, Monteith LL, . Military sexual trauma in the United States: results from a population-based study. J Affect Disord. 2022;306:19-27. doi:10.1016/j.jad.2022.03.016 35301038

[7] Blosnich JR, Montgomery AE, Dichter ME, . Social determinants and military veterans’ suicide ideation and attempt: a cross-sectional analysis of electronic health record data. J Gen Intern Med. 2020;35(6):1759-1767. doi:10.1007/s11606-019-05447-z 31745856 PMC7280399

[8] Galovski TE, Street AE, Creech S, Lehavot K, Kelly UA, Yano EM. State of the knowledge of VA military sexual trauma research. J Gen Intern Med. 2022;37(suppl 3):825-832. doi:10.1007/s11606-022-07580-8 36042078 PMC9481813

[9] Holliday R, Monteith LL. Seeking help for the health sequelae of military sexual trauma: a theory-driven model of the role of institutional betrayal. J Trauma Dissociation. 2019;20(3):340-356. doi:10.1080/15299732.2019.1571888 30714879

[10] Monteith LL, Holliday R, Hoyt T, Bahraini NH. Military sexual trauma and suicidal self-directed violence: a narrative review and proposed agenda for future research. In: Kumar U, ed. The Routledge International Handbook of Military Psychology and Mental Health. Taylor & Francis; 2019:389-410. doi:10.4324/9780429281266-26

[11] Kelly UA. Barriers to PTSD treatment-seeking by women veterans who experienced military sexual trauma decades ago: the role of institutional betrayal. Nurs Outlook. 2021;69(3):458-470. doi:10.1016/j.outlook.2021.02.002 33863545

[12] Andresen FJ, Monteith LL, Kugler J, Cruz RA, Blais RK. Institutional betrayal following military sexual trauma is associated with more severe depression and specific posttraumatic stress disorder symptom clusters. J Clin Psychol. 2019;75(7):1305-1319. doi:10.1002/jclp.22773 30947374

[13] Monteith LL, Holliday R, Schneider AL, Miller CN, Bahraini NH, Forster JE. Institutional betrayal and help-seeking among women survivors of military sexual trauma. Psychol Trauma. 2021;13(7):814-823. doi:10.1037/tra0001027 33764096

[14] Steenkamp MM, Litz BT, Marmar CR. First-line psychotherapies for military-related PTSD. JAMA. 2020;323(7):656-657. doi:10.1001/jama.2019.20825 31999301

[15] Maguen S, Madden E, Holder N, . Effectiveness and comparative effectiveness of evidence-based psychotherapies for posttraumatic stress disorder in clinical practice. Psychol Med. 2023;53(2):419-428. 34001290 10.1017/S0033291721001628 PMC9899565

[16] US Department of Veterans Affairs, Department of Defense. Management of Posttraumatic Stress Disorder and Acute Stress Disorder. Version 3.0. Dept of Defense; 2017.

[17] Taylor SL, Hoggatt KJ, Kligler B. Complementary and integrated health approaches: what do veterans use and want. J Gen Intern Med. 2019;34(7):1192-1199. doi:10.1007/s11606-019-04862-6 31011973 PMC6614301

[18] Davis LW, Schmid AA, Daggy JK, . Symptoms improve after a yoga program designed for PTSD in a randomized controlled trial with veterans and civilians. Psychol Trauma. 2020;12(8):904-912. doi:10.1037/tra0000564 32309986

[19] Taylor J, McLean L, Korner A, Stratton E, Glozier N. Mindfulness and yoga for psychological trauma: systematic review and meta-analysis. J Trauma Dissociation. 2020;21(5):536-573. doi:10.1080/15299732.2020.1760167 32453668

[20] Zaccari B, Sherman ADF, Febres-Cordero S, Higgins M, Kelly U. Findings from a pilot study of Trauma Center Trauma-Sensitive Yoga versus cognitive processing therapy for PTSD related to military sexual trauma among women veterans. Complement Ther Med. 2022;70:102850. doi:10.1016/j.ctim.2022.102850 35820575 PMC9704511

[21] Sciarrino NA, DeLucia C, O’Brien K, McAdams K. Assessing the effectiveness of yoga as a complementary and alternative treatment for post-traumatic stress disorder: a review and synthesis. J Altern Complement Med. 2017;23(10):747-755. doi:10.1089/acm.2017.0036 28708415

[22] Kelly U, Haywood T, Segell E, Higgins M. Trauma-sensitive yoga for post-traumatic stress disorder in women veterans who experienced military sexual trauma: interim results from a randomized controlled trial. J Altern Complement Med. 2021;27(S1):S45-S59. doi:10.1089/acm.2020.0417 33788599

[23] Emerson D. Trauma-sensitive yoga: principles, practice, and research. Int J Yoga Therap. 2009;19(1):123-128. doi:10.17761/ijyt.19.1.h6476p8084l22160

[24] Emerson D, Hopper E, Levine PA, Cope S, Van Der Kolk B. Overcoming Trauma Through Yoga: Reclaiming Your Body. North Atlantic Books; 2011.

[25] Zaccari B, Sherman ADF, Higgins M, Ann Kelly U. Trauma Center Trauma-Sensitive Yoga vs. cognitive processing therapy for women veterans with PTSD who experienced military sexual trauma: a feasibility study. J Am Psychiatr Nurses Assoc. Published online July 14, 2022. doi:10.1177/10783903221108765 35833676 PMC9839891

[26] Zaccari B, Loftis JM, Haywood T, Hubbard K, Clark J, Kelly UA. Synchronous telehealth yoga and cognitive processing group therapies for women veterans with posttraumatic stress disorder: a multisite randomized controlled trial adapted for COVID-19. Telemed J E Health. 2022. doi:10.1089/tmj.2021.0612 35357957 PMC9519809

[27] Weathers FW, Bovin MJ, Lee DJ, . The Clinician-Administered PTSD Scale for *DSM-5* (CAPS-5): development and initial psychometric evaluation in military veterans. Psychol Assess. 2018;30(3):383-395. doi:10.1037/pas0000486 28493729 PMC5805662

[28] Blevins CA, Weathers FW, Davis MT, Witte TK, Domino JL. The Posttraumatic Stress Disorder Checklist for *DSM-5* (PCL-5): development and initial psychometric evaluation. J Trauma Stress. 2015;28(6):489-498. doi:10.1002/jts.22059 26606250

[29] American Psychological Association. Diagnostic and Statistical Manual of Mental Disorders. 5th ed. American Psychological Association; 2013.

[30] van der Kolk B. The Body Keeps The Score: Brain, Mind, and Body in the Healing of Trauma. Viking; 2014.

[31] Herman JL. Trauma and Recovery: The Aftermath of Violence—From Domestic Abuse to Political Terror. Hachette Book Group; 1992.

[32] Balasubramaniam M, Telles S, Doraiswamy PM. Yoga on our minds: a systematic review of yoga for neuropsychiatric disorders. Front Psychiatry. 2013;3:117. 23355825 10.3389/fpsyt.2012.00117 PMC3555015

[33] Boehler J. The efficacy of cognitive processing therapy for PTSD related to military sexual trauma in veterans: a review. J Evid Based Soc Work (2019). 2019;16(6):595-614. doi:10.1080/26408066.2019.1666767 32459157

[34] Resick PA, Monson CM, Chard KM. Cognitive Processing Therapy Veteran/Military Version: Therapist and Patient Materials Manual. Dept of Veterans Affairs; 2014.

[35] Cohen J. Statistical Power Analysis for the Behavioral Sciences. 2nd ed. Academic Press; 2013. doi:10.4324/9780203771587

[36] *Power Analysis and Sample Size Software (PASS)*. NCSS; 2021.

[37] Hedeker DR, Gibbons RD. Longitudinal Data Analysis. Wiley-Interscience; 2006.

[38] van der Kolk BA, Stone L, West J, . Yoga as an adjunctive treatment for posttraumatic stress disorder: a randomized controlled trial. J Clin Psychiatry. 2014;75(6):e559-e565. doi:10.4088/JCP.13m08561 25004196

[39] Maguen S, Holder N, Madden E, . Evidence-based psychotherapy trends among posttraumatic stress disorder patients in a national healthcare system, 2001-2014. Depress Anxiety. 2020;37(4):356-364. doi:10.1002/da.22983 31850650

[40] Khan AJ, Holder N, Li Y, . How do gender and military sexual trauma impact PTSD symptoms in cognitive processing therapy and prolonged exposure? J Psychiatr Res. 2020;130:89-96. doi:10.1016/j.jpsychires.2020.06.025 32798774

[41] Lakens D. Equivalence tests: a practical primer for *t* tests, correlations, and meta-analyses. Soc Psychol Personal Sci. 2017;8(4):355-362. doi:10.1177/1948550617697177 28736600 PMC5502906

[42] Lakens D, Scheel AM, Isager PM. Equivalence testing for psychological research: a tutorial. Adv Methods Pract Psychol Sci. 2018;1(2):259-269. doi:10.1177/2515245918770963

[43] Sloan DM, Marx BP, Resick PA, ; STRONG STAR Consortium. Effect of written exposure therapy vs cognitive processing therapy on increasing treatment efficiency among military service members with posttraumatic stress disorder: a randomized noninferiority trial. JAMA Netw Open. 2022;5(1):e2140911. doi:10.1001/jamanetworkopen.2021.40911 35015065 PMC8753496

[44] Schnurr PP, Chard KM, Ruzek JI, . Comparison of prolonged exposure vs cognitive processing therapy for treatment of posttraumatic stress disorder among us veterans: a randomized clinical trial. JAMA Netw Open. 2022;5(1):e2136921. doi:10.1001/jamanetworkopen.2021.36921 35044471 PMC8771295

[45] SPSS Statistics for Windows. Version 27.0. IBM Corporation; 2020.

[46] R: A Language and Environment for Statistical Computing. R Core Team; 2021.

[47] Berke DS, Kline NK, Wachen JS, ; STRONG STAR Consortium. Predictors of attendance and dropout in three randomized controlled trials of PTSD treatment for active duty service members. Behav Res Ther. 2019;118:7-17. doi:10.1016/j.brat.2019.03.003 30933748

[48] Steenkamp MM, Litz BT, Hoge CW, Marmar CR. Psychotherapy for military-related PTSD: a review of randomized clinical trials. JAMA. 2015;314(5):489-500. doi:10.1001/jama.2015.8370 26241600

[49] Pacella ML, Hruska B, Delahanty DL. The physical health consequences of PTSD and PTSD symptoms: a meta-analytic review. J Anxiety Disord. 2013;27(1):33-46. doi:10.1016/j.janxdis.2012.08.004 23247200

[50] Lohr JB, Palmer BW, Eidt CA, . Is post-traumatic stress disorder associated with premature senescence? a review of the literature. Am J Geriatr Psychiatry. 2015;23(7):709-725. doi:10.1016/j.jagp.2015.04.001 25959921 PMC4568841

[51] Streeter CC, Gerbarg PL, Saper RB, Ciraulo DA, Brown RP. Effects of yoga on the autonomic nervous system, gamma-aminobutyric-acid, and allostasis in epilepsy, depression, and post-traumatic stress disorder. Med Hypotheses. 2012;78(5):571-579. doi:10.1016/j.mehy.2012.01.021 22365651

[52] Kelly UA, Evans DD, Baker H, Noggle Taylor J. Determining psychoneuroimmunologic markers of yoga as an intervention for persons diagnosed with PTSD: a systematic review. Biol Res Nurs. 2018;20(3):343-351. doi:10.1177/1099800417739152 29130314

[53] Sullivan MB, Erb M, Schmalzl L, Moonaz S, Noggle Taylor J, Porges SW. Yoga therapy and polyvagal theory: the convergence of traditional wisdom and contemporary neuroscience for self-regulation and resilience. Front Hum Neurosci. 2018;12:67. doi:10.3389/fnhum.2018.00067 29535617 PMC5835127

[54] Gard T, Noggle JJ, Park CL, Vago DR, Wilson A. potential self-regulatory mechanisms of yoga for psychological health. Front Hum Neurosci. 2014;8:770. doi:10.3389/fnhum.2014.00770 25368562 PMC4179745

[55] Kabat-Zinn J. Mindfulness-based stress reduction. In: Catastrophe Living: Using the Wisdom of Your Body and Mind to Face Stress, Pain, and Illness. Bantum Books; 1990:467.

