# Supplementary material for: Yoga vs Cognitive Processing Therapy for Military Sexual Trauma–Related Posttraumatic Stress Disorder: A Randomized Clinical Trial
Source: JAMA Netw Open. 2023 Dec 8;6(12):e2344862. doi: 10.1001/jamanetworkopen.2023.44862 (PMC10709771; doi:10.1001/jamanetworkopen.2023.44862)
Supplement: Supplement 1. — Trial Protocol [file jamanetwopen-e2344862-s001.pdf]

1. Study protocol (Study protocol both sites and COVID Contingency)

IRB Protocol

Full Title: A Randomized Controlled Trial Comparing Trauma Center-Trauma Sensitive Yoga and Cognitive Processing Therapy for PTSD and Associated Symptoms in Women Veterans

Short Title: RCT of Yoga versus CPT in Women Veterans (Project Stress-Less II)

Coordinating Center (Atlanta):

Principal Investigator: Ursula A. Kelly, PhD, ANP-BC, PMHNP-BC

Nurse Scientist
Atlanta VA Medical Center, Trauma Recovery Program

Local Site (Portland):

Principal Investigator: Belle Zaccari, PsyD

Staff Psychologist
VA Portland Health Care Center, Rural Telemental Health

Sponsor Information
This study is funded by VA HSR&D Nursing Research Initiative award NRI 15-151.

A. SPECIFIC AIMS

This project builds directly on a one-year pilot study (Project Stress-Less) conducted by the PI which demonstrated the feasibility of the current protocol. This study will test the effectiveness of a Trauma Center- Trauma Sensitive Yoga (TC-TSY) intervention to improve the health, social functioning, and quality of life for women Veterans with posttraumatic stress disorder (PTSD) related to military sexual trauma (MST). The randomized controlled trial (RCT) will compare results from a TC-TSY group (experimental group) to an evidence based PTSD treatment known as Cognitive Processing Therapy- (CPT; control group). Outcomes to be assessed include PTSD symptom severity, levels of chronic pain and insomnia, quality of life, social functioning and objective measures of biological and psychophysiological stress. We hypothesize that the TC- TSY intervention will reduce PTSD symptoms and psychophysiological responsivity to stress which could lead to fewer medical consequences of PTSD and improve functioning and quality of life in this population. New, evidence-based PTSD treatments to supplement current approaches are needed and this RCT has the potential to fill this gap.

Women Veterans experience MST and PTSD at alarming rates; consistently reported prevalence rates among VA patient samples are 21%-40% for MST and 20% for PTSD[1, 2]. Women Veterans who experienced MST and have PTSD exhibit a variety of clinically significant physical and psychological symptoms, including chronic pain, insomnia, and depression[3]. **Although there are evidence-based, cognitively-oriented treatments for PTSD, not all individuals respond to or agree to participate in these; therefore, new treatments from innovative theoretical perspectives are needed.**

Evidence indicates that yoga reduces stress and pain, and enhances sleep and functioning in civilian populations[4]. Results from pilot investigations demonstrate that yoga is beneficial in reducing PTSD symptoms in survivors of natural disasters[5], as well as those with chronic PTSD[6,7] and combat-related PTSD[8]. A small number of studies indicate that yoga interventions are feasible and acceptable to U.S. Veterans[8,9]. Overall, the science of yoga is in early development, and studies examining the beneficial effects of yoga have been limited by methodological problems including small sample sizes, inadequate power, lack of randomization, and inconsistency among the yoga methods tested. In addition, these studies employed divergent measures to evaluate the effects of yoga with a lack of objective measurement of outcomes including biological or psychophysiological markers that are associated with previous psychological trauma and with PTSD symptoms. These include inflammatory markers, exaggerated acoustic startle response to darkness (termed dark-enhanced startle), and depressed heart rate variability[10-13]. In addition, studies that recruit Veterans with PTSD often disproportionately under-represent or do not include data specific to women; furthermore, sexual trauma is typically not considered, although it is the most common cause of PTSD among women Veterans.

In 2014, our team conducted a one-year NRI pilot study (n=42), referred to as *Project Stress-Less*, which was the precursor to the current NRI study. In Project Stress-Less, a trauma-sensitive Hatha yoga intervention was conducted in parallel to a Cognitive Processing Therapy-Cognitive group (CPT-C) in a sample of women Veterans with PTSD related to MST. Hatha yoga is a gentle, slow approach to physical postures and includes breath work and mindfulness. The specific aims of Project Stress-Less were to: assess the feasibility of recruitment and retention (including randomization and intervention acceptability) in yoga versus evidence-based group psychotherapy; to evaluate study participants' adherence and yoga teachers' fidelity to the yoga intervention; and to assess the feasibility of obtaining biological and psychophysiological data for a study of yoga as a treatment for PTSD in this population. We succeeded in achieving these aims. In addition, our results support our hypothesis for the

proposed study that this Trauma Center-Trauma Sensitive Yoga intervention may be effective in reducing PTSD symptoms, chronic pain, insomnia, and biological and psychophysiological hyper-responsivity. The proposed RCT is the culmination of the successful implementation and feasibility demonstrated throughout the one-year Project Stress-Less pilot study.

The specific aims and hypotheses of the proposed RCT:

Aim 1: To evaluate the effectiveness of Trauma Center-Trauma Sensitive Yoga (TC-TSY) compared to Cognitive Processing Therapy (CPT) in reducing PTSD symptoms, chronic pain, and insomnia in women Veterans with PTSD related to MST.

Hypothesis 1: Participants in the TC-TSY group will show statistically and clinically meaningful reductions in PTSD symptoms, chronic pain and insomnia (PTSD Checklist-5 (PCL-5) scores, Clinician Administered PTSD Scale (CAPS-5) scores, Brief Pain Inventory (BPI) scores, Pittsburgh Sleep Quality Index (PSQI) scores compared to CPT-C group results following treatment.

Aim 2: To evaluate the effectiveness of TC-TSY as compared to CPT in improving quality of life and social functioning in women Veterans with PTSD related to MST.

Hypothesis 2: Participants in the TC-TSY group will show statistically and clinically meaningful improvements in quality of life and social functioning (VR-12, PROMIS measures) compared to CPT group results.

Aim 3: To evaluate the effectiveness of TC-TSY as compared to CPT on biological stress response and psychophysiological hyper-responsivity.

Hypothesis 3: Participants in the TC-TSY group will show statistically and clinically meaningful changes in biological stress response (inflammatory cytokines (IL-6, IL-10), C-reactive protein levels) and psychophysiological hyper-responsivity (dark-enhanced startle measures and heart rate variability)

B. BACKGROUND AND SIGNIFICANCE

Women represent 9.2% of the total Veteran population[14]. There are currently over two million women Veterans, 20%-27% of whom have PTSD[15]. This number is expected to grow significantly over the next decade. While the VA is working diligently to develop interventions for women Veterans, the continuing increase in numbers means that the development of evidence-based treatments optimized for this population is essential. Women Veterans often are exposed to a host of traumas across their lifespan, including childhood trauma, civilian trauma (e.g., intimate partner violence, sexual assault, physical abuse, emotional abuse), MST, and combat exposure[3,16]. Given that both PTSD and exposure to interpersonal violence are associated with multiple psychological and physical symptoms, medical problems and suicide, targeted interventions for this population are critical.

Current PTSD treatment guidelines almost universally recommend exposure therapy and cognitive- behavior therapies[17]. Eye movement desensitization and reprocessing is also recommended in most guidelines. However, despite substantial evidence of the effectiveness of these interventions for some individuals, there are many others whose symptoms do not improve with these treatments. Even with treatment, as many as one-third of individuals with PTSD experience substantial or disabling distress that persists for years or even decades[18]. **In addition, both chronic pain and sleep problems are resistant to standard PTSD treatments[16,19].** Even in those individuals for whom some PTSD symptoms improve, residual PTSD symptoms including pain and insomnia, affective and attention regulation difficulties, and loss of meaning in life can persist[20]. Further, some individuals with PTSD avoid exposure therapy and other trauma- focused cognitive-behavioral interventions because of fear of increased distress and the belief that they will be unable to tolerate this added distress[21,22]. Dropout and non-response rates in outcome studies using standard PTSD treatments often exceed 50% of the sample[23]. In one PTSD treatment study, combined skills training, support groups and exposure therapy were used with women with chronic trauma, and dropout rates ranged from 15.2%-39.4% across study arms[24].

The limitations of current evidence-based therapies for PTSD suggest the need for further investigation into novel approaches from different theoretical perspectives for PTSD treatments that are acceptable to patients, effective, and that address the constellation of symptoms associated with PTSD, including chronic pain and insomnia. This need prompted the Project Stress-Less pilot study which built a solid foundation for the implementation of the current RCT. This RCT is being conducted in order to further study a complementary and alternative PTSD treatment that could supplement current evidence-based modalities for this population and that could be disseminated to VA Medical Centers (VAMC’s) nationwide.

There is growing interest in complementary and alternative therapy modalities to treat mental health disorders in the VA[25,26]. A majority of VA Medical Centers report using complementary and alternative therapies as elements in their PTSD treatment programs[27]. However, the utilization of these modalities varies widely, the interventions are not evidence-based or consistently provided by trained personnel, and they are not evaluated for effectiveness. Research leading to new developments in PTSD treatment for women Veterans not only has significant positive potential for this population, but also can be more broadly applied to trauma-exposed civilian women, particularly those who have experienced sexual trauma, for whom evidence- based complementary and alternative therapy interventions are lacking.

Health Impact of MST and PTSD

Women Veterans who experienced MST are at risk for a wide range of trauma-related

health problems including PTSD, depression, anxiety, chronic pain, sleep disturbances, wide-ranging physical symptoms, substance abuse, and negative health behaviors[3]. For example, compared to women with no prior history of sexual assault, women Veterans who experienced MST had a nine-fold risk for PTSD[28]. PTSD is a strong predictor of suicidal ideation and suicide attempts for both men and women[29]. **In 2009 and 2010, PTSD was the top diagnosis for women Veterans treated by the VA[30].** These multiple symptoms and health problems may be compounded in this population due to high rates of additional trauma (e.g., combat trauma, intimate partner violence, and/or child abuse)[16]. The multiple health problems for which this population is at increased risk can result in significant health problems, disability, decreased quality of life and impaired social functioning [19, 31, 32].

Chronic pain is a persistent problem for women Veterans.

Dobie and colleagues[32] reported that, in their study of nearly 2000 female Veterans, those with PTSD were more than twice as likely to report bodily pain in the previous month. Co-morbidity between PTSD and pain is as high as 80% in the general and Veteran populations[33,34]. Chronic pain and insomnia are common problems in female survivors of multiple trauma exposures, particularly interpersonal violence and childhood trauma[16, 35-37]. The interplay between PTSD symptoms, chronic pain, and insomnia creates a symptom cluster in which individual symptoms cause or exacerbate other symptoms synergistically[38]. Of the four diagnostic PTSD symptom clusters (re-experiencing, avoidance, negative cognitions and mood, and arousal), hyperarousal symptoms may account for more reported health problems in women Veterans than do re-experiencing and numbing symptom clusters[39]. Logically, an intervention that targets multiple symptoms within this broader symptom cluster (PTSD symptoms, chronic pain, and insomnia) is most likely to be effective.

Scientific Rationale

Trauma and Women Veterans.

Women Veterans often have high rates of pre-military, military, and post-military trauma. In an integrated literature review, Zinzow (2007) and colleagues found that more than 80% of women Veterans reported at least one type of trauma and had higher rates of trauma than their civilian counterparts[3]. In a study we conducted in the Atlanta VA Medical Center, we found that of 135 participants, 95.4% reported at least one trauma in addition to MST, most notably sexual abuse as adult civilians (77.0%) and as children (52.6%). Nearly one in four reported experiencing all three types of childhood abuse: emotional, physical and sexual[16]. Given the high rates of cumulative trauma in addition to MST among women Veterans, particularly those with PTSD, it is not feasible to isolate the effects of MST on autonomic nervous system responses, for example the hyperarousal associated with PTSD. The rationale behind our focus on MST in this study is related to a PTSD intervention specific to women who experienced MST. Although the focus of our study is MST, we hope that our findings may increase overall understanding of the immunological and neurobiological factors associated with trauma exposure. In this study, we will investigate autonomic nervous system and inflammatory responses associated with PTSD. Detailed descriptions of these immunological and psychophysiological responses and how they relate to trauma, pain, and sleep are provided below. This will establish the foundation of the theoretical framework for this study, that is, the postulated counter-acting effect of yoga on harmful inflammatory and psychophysiological manifestations of psychological trauma and PTSD.

Psychophysiology of PTSD

Understanding the neurobiology of PTSD is central to understanding the potential impact of yoga on PTSD and its associated symptoms of chronic pain and insomnia. PTSD is associated with altered neurobiological responses, including autonomic nervous system dysfunction, notably increased sympathetic nervous system activity and decreased parasympathetic nervous system activity. Under most circumstances, the sympathetic nervous system functions in opposition to the parasympathetic nervous system. Early and/or repeated exposure to extreme stress or trauma affects an individual's long-term capacity to modulate the sympathetic nervous system and parasympathetic nervous system responses to subsequent stress. Activation of the sympathetic nervous system is traditionally associated with the "fight or flight" response to an extreme trauma, which is characterized by increased heart rate, increased cardiac output, and increased blood pressure. While activation of the parasympathetic nervous system is characterized by reduced heart rate, reduced cardiac output, and reduced blood pressure. Inadequate parasympathetic nervous system response tips the sympathetic nervous system/parasympathetic nervous system balance towards hyperarousal, difficulty in responding to stressors, and an impaired relaxation response, all of which are characteristic in posttraumatic stress responses. Heightened sympathetic nervous system activity is marked by an increased basal heart rate, a robust cardiovascular marker of PTSD.

Inflammatory Processes, Trauma, Pain, and Sleep

PTSD and chronic pain are also associated with immunologic responses, including inflammatory processes such as the inflammatory cytokine cascade. Inflammatory cytokines and C-reactive protein have been associated in various studies with trauma exposure, subsequent PTSD, and chronic pain. There is a relationship between sleep and the immune response; however the association of cytokines with sleep disturbances is inconclusive. Given these relationships, cytokines and C-reactive protein may be key biomarkers of the effect of yoga on PTSD symptoms, chronic pain, and insomnia.

Inflammatory Cytokines, C-Reactive Protein, Trauma Exposure, and PTSD

As with other physiological systems, the sympathetic nervous system and parasympathetic nervous system have opposite effects on inflammation and the immune response. Sympathetic nervous system stimulation of white blood cells, via the release of the neurotransmitter norepinephrine and its subsequent binding to alpha- adrenergic receptors, increases pro-inflammatory cytokine release from macrophages, neutrophils, and T cells[40]. In stark contrast, parasympathetic nervous system stimulation of surrounding macrophages and other white blood cells, via the release of the neurotransmitter acetylcholine, inhibits pro-inflammatory cytokine release[40]. Elevated levels of inflammatory markers have been found in survivors of trauma (interleukin-6 (IL-6)) and individuals with PTSD (C-reactive protein; interleukin-1 (IL-1); IL-6, IL-10)[41,42]. Alterations in C-reactive protein and cytokine activity are also associated with symptoms that commonly co-occur with PTSD, including depressive symptoms, fatigue, chronic tissue inflammation, and enhanced sensitivity to pain[43]. Elevated inflammatory markers are also associated with "sickness behavior," an inflammation-related syndrome characterized by depression, fatigue, reduced physical activity, changes in sleep and appetite, and an increased responsiveness to pain[44].

Inflammatory Cytokines and Pain Response

The relationship between inflammatory markers and pain suggests that these biomarkers may also be useful in understanding the mechanism by which chronic pain may be maintained in patients with PTSD. Elevations in pro-inflammatory cytokines (IL-1β, IL-2, IL-6, TNF-α) and C-reactive protein have been shown to correlate with increasing pain

intensity in patients with chronic pain[43], psychological stress[45] and PTSD[46]. The mechanisms by which inflammatory cytokines are believed to cause hyperalgesia are attributed to neuroplastic changes within the peripheral and central nervous system, which alter nociceptive signaling, and as such, can increase sensitivity[47]. IL-6 has been shown to act as a messenger relaying chemotactic peripheral immune signals to the central nervous system[43]. Similarly, glial cells within the brain secrete cytokines, further altering peripheral nociceptive responses affecting the subjective experience of pain[43]. This complex interplay is hypothesized to contribute not only to pain, but also to fatigue and depressive symptomatology[48].

Inflammatory Cytokines, C-Reactive Protein, and Sleep Impairment

There is a bidirectional relationship between sleep and the immune response. C-reactive protein and cytokines, including TNF, IL-1, IL-4, IL-6, and IL-10, have been established as part of the biochemical sleep regulatory process[49]. In addition to changes in C-reactive protein and cytokine activity[49,50], disordered sleep is associated with changes in stress hormone levels and reduced heart rate variability, indicative of greater sympathetic nervous system activity[51]. Because the associations between sleep and cytokines remains inconclusive and because it is unclear how cytokine levels relate to subjective and objective indices of sleep quality, further investigation of the role of cytokines in sleep in women with PTSD is needed. Pain disrupts sleep, with a majority of patients with chronic pain (50% to 70%) reporting poor sleep quality[52]. Because pain and disrupted sleep often co-exist, it is difficult to determine whether poor sleep or pain is triggering changes in immune function. **Taken together, these data on the relationships between inflammatory markers, chronic pain, and sleep disturbances suggest that C-reactive protein and certain cytokines may be key biomarkers of the effects of yoga on symptoms of PTSD and associated insomnia and chronic pain.**

Research on Yoga as a Therapeutic Intervention

Effectiveness of Yoga for Depression, Anxiety, Insomnia, and Physical Pain

Yoga is increasingly being studied as a therapeutic intervention for a variety of physical and mental health problems and symptoms[53]. Growing evidence demonstrates that yoga is safe and effective in treating depression and anxiety[54,55], and improving sleep quality[56] and insomnia in post-menopausal women[57]. Yoga has also been found to significantly reduce general musculoskeletal pain and disability[58,59]. The majority of researchers investigating yoga as a treatment for pain have focused on chronic low back pain. Groessl et al. studied yoga for chronic low back pain in male and female Veterans and found that while both women and men had significant reduction in pain and depressive symptoms with increased energy and mental health-related quality of life, women had significantly larger benefits than men[60]. PTSD symptoms were not evaluated in this study, yet 30% of the men and 15% of the women in the sample reported having a PTSD diagnosis.

Yoga in the Treatment of Trauma-Exposed Individuals

Yoga has been shown to reduce anxiety and depression in male and female active duty military personnel, as well as male and female Veterans with PTSD[9,61,62]. Trauma Center-Trauma Sensitive Yoga, the modality to be used for the current study, was developed specifically for civilian women survivors of childhood sexual trauma with PTSD[6,63]. Trauma Center-Trauma Sensitive Yoga focuses on interoception (i.e., the sense of the physiological condition of the body) and links breath with movement, builds body awareness and self-

regulation, and uses invitational rather than instructional or commanding language. Emerson, a member of our research team, and colleagues (2014) found that Trauma Center-Trauma Sensitive Yoga decreased symptoms of PTSD in a study of civilian women (n=60) with chronic PTSD[7]. We have evaluated Trauma Center-Trauma Sensitive Yoga with a different and under-studied population, women Veterans with PTSD who experienced MST and reported chronic pain and insomnia, in two preliminary projects preceding the development of this RCT.

Within the few studies of yoga as a potential intervention for PTSD, most researchers have relied on self- report data. **In Project Stress-Less, we established the feasibility of using objective outcome measures such as inflammatory biomarkers (cytokines and C-reactive protein) and psychophysiological markers, including the acoustic startle response and heart rate variability.** Emerging research findings strongly suggest that yoga is an effective therapeutic intervention for the symptoms to be evaluated in this study.

However, the body of research examining the beneficial effects of yoga is limited by methodological problems including inadequate power, lack of randomization, and inconsistency among the yoga procedures and measures used to assess yoga effects[25,26]. In addition, studies that recruit Veterans with PTSD often disproportionately under-represent or do not include data specific to women; furthermore, sexual trauma is typically not considered, although it is the most common cause of PTSD among women Veterans.

<u>Psychophysiological Markers Associated with Trauma Exposure and PTSD</u>

Heart Rate Variability (HRV)

Evaluation of parasympathetic nervous system and sympathetic nervous system activity is typically accomplished via measurement of HRV. HRV reflects the central nervous system's ability to respond immediately to fluctuations in blood pressure occurring with each beat; high-frequency heart rate variability indicates fine control via a parasympathetic nervous system-dominant response while low-frequency heart rate variability reflects both sympathetic nervous system and parasympathetic nervous system activity. HRV is measured via electrocardiographic monitoring of the R-R interval. Computerized power spectral analysis is used to identify low-frequency versus the high-frequency components of HRV, reflecting sympathetic nervous system and parasympathetic nervous system activity. Decreased HRV has been correlated with morbidity and mortality from diverse diseases, including anxiety and depression[64] and cardiovascular disease[65]. The relationship of decreased HRV to morbidity and mortality in these disorders is believed to be related to the inverse relationship between HRV and inflammatory markers: that is, the lower the HRV, the higher the level of inflammatory markers. Heart rate variability has been empirically studied in patients with PTSD; most researchers report decreased high-frequency heart rate variability during rest[66]. Heart rate variability measures demonstrate gender differences; a recent study found that increased heart-rate response to trauma reminders soon after trauma exposure predicted PTSD diagnosis six months later in women but not men[67]. Lower heart rate variability has recently been found to correlate with MST in women Veterans with PTSD[68]. **The proposed study will measure heart rate variability as a psychophysiological marker to assess intervention outcomes.**

Dark Enhanced Startle

Dark-enhanced startle is an ecologically valid psychophysiological paradigm for assessing contextual levels of fear and anxiety. We have previously shown the magnitude of the acoustic startle (blink) response is exaggerated in previously traumatized women

when assessed in darkness versus light conditions[69]. Using startle measures, studies have shown that patients with PTSD show overgeneralization of fear and an inability to inhibit fear responses in the presence of safety[70]. Impaired fear inhibition may be a specific biomarker of PTSD[70], specifically autonomic hyperarousal. In humans, the startle response is increased in darkness as compared to well-lit environments, a phenomenon termed dark-enhanced startle. Dark-enhanced startle is a laboratory analogue of sustained anxiety and represents a clinically useful tool for assessing anxiety-like behaviors and hyperarousal as they relate to symptom severity.

Psychophysiological Markers Associated with Pain Response

Vascular parameters, including heart rate and blood pressure, skin conductance measures and electromyographic readings, are the most commonly used physiological markers of patients' subjective experiences of chronic pain syndromes. However, these measures may be differentially influenced if the pain syndrome stems from sympathetic nervous system hyperarousal (complex pain syndromes), musculoskeletal (back pain) or vascular origins (migraine headache), as well as the individual's psychological coping abilities. Experimental studies in healthy men demonstrate that increasing levels of pain are associated with increases in heart rate[71]; however, this association has not been found in women. Changes in heart rate variability may be a better measure of reduced hyperarousal and pain in women than heart rate, although the relationship between heart rate variability and pain in women is complex and may vary based on whether the pain is acute or chronic. The proposed study will add a better understanding of the relationship between heart rate variability and pain in women, specifically in those with PTSD.

Theoretical Framework

The proposed study is based on a theoretical framework in which the impact of the yoga intervention is conceived to directly counteract immunological and psychophysiological responses to trauma. Our proposed model suggests that, through its effects on the central nervous system, yoga reduces the inflammation and psychophysiological hyperarousal associated with trauma and improves autonomic regulation. **We propose that these effects (reduced inflammation and hyperarousal and improved autonomic regulation) are one pathway through which the practice of yoga may lead to reduced PTSD symptoms, chronic pain and insomnia.** Because of the data suggesting a bidirectional relationship between inflammation/autonomic dysregulation/hyperarousal and PTSD symptoms/chronic pain/insomnia, it is also expected that the yoga intervention will facilitate improved functioning in both domains (inflammation/autonomic dysregulation/hyperarousal and PTSD symptoms/chronic pain/insomnia). It is hoped that this synergistic improvement across the two domains will counteract the alternate process in which increases in one domain lead to increases in the second, thereby increasing the overall negative impact of trauma exposure.

In other words, our theory proposes that the practice of yoga produces a relaxation response that counteracts the physiological stress associated with PTSD and other associated trauma-related chronic health problems[56]. As described above, the beneficial effects of yoga on stress are postulated to result from control of physiologic reactivity, decreased sympathetic activity and activation of the parasympathetic nervous system[4,72], since yoga is associated with increased heart rate variability[72,73]. Slow and rhythmic yoga breathing techniques have been found to synchronize sympathetic nervous system and parasympathetic nervous system vagal activity outflow, resulting in increased heart rate variability and arterial baroreceptor sensitivity characterized by a sense of calm[74,75]. The evidence that yoga increases the ability to focus attention, calm the mind and relax

the body[4,72], resulting in reduced respiratory rates[74] and increased cardiac vagal modulation and heart rate variability[72,73], suggests that yoga may reduce the acoustic startle response by decreasing hyperarousal, a finding supported in Project Stress-Less. Additionally, preliminary research demonstrates that yoga is associated with reduced C-reactive protein and inflammatory cytokines, specifically IL-6[76].

This RCT will investigate a **novel approach to treating not only PTSD symptoms, but chronic pain and insomnia,** which appear to be the most intractable symptoms associated with MST and PTSD[19]. Our theoretical framework suggests that yoga will reduce PTSD symptoms, chronic pain, and insomnia via psychophysiological mechanisms, rather than the cognitive mechanisms of current psychotherapeutic treatments. Further, **the anti-inflammatory mechanism of yoga may minimize inflammatory sequelae of MST and PTSD, thereby reducing conditions with which PTSD is co-morbid and, in turn, improving general health.**

Preliminary Data

Trauma Exposure and Mental and Physical Health Symptoms in Women Veterans with MST and PTSD In a study of women in a VA Women's Trauma Program (n=200), Kelly (PI) et al found that the women's PTSD symptoms were highly correlated with major depression (r=.715; p<.001), sleep difficulty (r=.484; p<.001), pain (r=.249; p<.01), and lower quality of life (r=.469; p<.001)[16]. In a random sample (n=50) of our Women's Trauma Program patients, 80% had chronic pain, e.g. low back pain (40%) and headaches (48%), unrelated to malignancy, injury or surgery in the previous three months. These data demonstrate the substantial co-morbid mental and physical health problems of women Veterans with PTSD and MST in our setting.

PTSD Treatment Seeking by Women Veterans who Experienced MST The PI and several co-investigators on the proposed study conducted a qualitative study to explore the barriers and facilitators to PTSD treatment seeking by the target population[79]. Participants were recruited from the Women's Trauma Program after completing the intake process. A constructivist grounded theory approach, was used to collect data via individual semi-structured interviews and to conduct data analysis. These patients (n=12) had clinically severe symptoms of PTSD (PCL = 59.62; clinically significant symptoms are defined as ≥ 50 in Veteran populations), major depression (BDI-II mean 33.18; severe symptoms are defined as ≥ 29), and somatic complaints (PHQ- 15 mean 30.10; severe symptoms are defined ≥ 15). **Several of the women in this study described physical pain, difficulty sleeping, perceived poor physical health, multiple diagnosed medical problems, and significant weight gain as reasons for seeking PTSD treatment.**

Project Stress-Less (NRI 12-417): Trauma Center-Trauma Sensitive Yoga for Women Veterans with PTSD symptoms and MST exposure. In the Project Stress-Less pilot study, we enrolled 42 women Veterans with PTSD related to MST who were seeking mental health treatment for PTSD in the Atlanta VAMC Women's Trauma Program and who reported chronic pain. Participants were randomly assigned to the two study conditions: Trauma Center-Trauma Sensitive Yoga or Cognitive Processing Therapy-Cognitive (CPT-C) groups. These groups did not differ significantly on age, race, or education, however the CPT-C group had higher clinician assessed PTSD severity (CAPS), depressive symptoms (BDI-II), and pain (POQ) at baseline. Of note, participants were notified of their group assignment prior to data collection. Several participants who were

randomly assigned to the CPT-C group expressed dismay at their assignment. One possible explanation of the higher symptom severity in this group at baseline is that some in CPT-C were disappointed in their randomization assignment. To address this possibility, in the proposed RCT, we will collect baseline data before randomization.

The TC-TSY protocol[7] involved ten weekly 90-minute group sessions. The standard CPT-C protocol was used and included twelve weekly 90-minute group sessions. We conducted two cohorts of the interventions concurrently and collected data at four time points: Baseline (T1), mid-intervention (T2), two-weeks post- intervention (T3) and three months post-intervention (T4).

The specific aims of Project Stress-Less were to assess the feasibility of recruitment, retention, intervention implementation and obtaining biological and psycho-physiological data. In addition, we looked at these measures to determine their use as outcome measures for yoga and PTSD research in this population. These aims were achieved. All data have been analyzed and are supportive of our hypotheses on the mental and physical benefits of the trauma sensitive yoga intervention. An outline of feasibility and intervention results is provided below.

Study Recruitment and Retention Feasibility

Retention of participants in the TC-TSY group was higher at each time point than in the CPT-C group. Of note, some individuals declined to participate or withdrew from the study without a guarantee of receiving the yoga intervention or when randomized to the CPT-C arm of the study. In the current RCT, potential participants will be informed that they can receive the intervention they are not assigned to after completion of their participation in the study at no cost.

We based our overall study attrition estimate on that of van der Kolk and colleagues[77]. However, our population and setting was different in several ways: 1) our sample had higher levels of complex trauma (Table 3); 2) our sample was less educated and had higher rates of unemployment; and 3) our setting in a VA Medical Center versus a private clinic was undesirable to participants, was not easily accessible by participants, and had limited parking. We are conservatively accounting for 50% retention in the current RCT, but hope to achieve higher retention with the implementation of several changes to the setting, protocol and retention strategies.

Intervention Feasibility: Completion Rates and Acceptability to Participants

We determined the number of intervention sessions and defined intervention completion based on published standards for the specific interventions. The TC-TSY intervention is a manualized curriculum of ten weekly sessions. Emerson and colleagues, who designed the intervention, defined intervention completion as attendance at seven of the ten sessions[7]. For the CPT-C group, treatment completion was defined as attendance at nine or more of the twelve sessions, based on the definition used in most empirical studies. However, it is important to note that in the Women's Trauma Program, drop-out rates for CPT-C groups average 50%. The retention rate in the TC-TSY groups (58.8%) was significantly higher than in the CPT-C groups (36%). The higher retention rates for the TC-TSY group could suggest that the yoga sessions may be a more acceptable form of treatment for the target population.

Qualitative data were collected via focus groups and individual interviews after the first session, mid- intervention and at post-intervention data collection points. Participants were receptive and engaged in TC- TSY sessions. Positive feedback given about TC-TSY was that participants found the intervention to be calming and helpful for stress reduction. Several also reported an increased awareness of their bodies as a result of yoga. Some

participants in the CPT-C group stated that they appreciated the process of learning how to challenge their thoughts, though many found the process difficult. Most participants in both groups found getting to the sessions difficult due to traffic and parking. Participants also shared that they would prefer to have the group sessions somewhere other than the VA, as this location served as a trigger for PTSD symptoms. Most recently, the Women's Trauma Program has moved from the main VA Medical Center to a community based outpatient clinic in a more accessible location, with ample parking, and a quieter environment. We anticipate that this new location may address the concerns voiced by previous participants in Project Stress-Less.

Intervention Fidelity

Group sessions were led by two yoga teachers experienced in teaching Trauma Center-Trauma Sensitive Yoga and who received 20 hours of in-person training and 20 hours of consultation calls from David Emerson, a collaborator on the pilot study and the current project. The teachers co-taught the sessions, allowing for peer monitoring and feedback. The teachers completed summary and debriefing sheets after each session and had consultation calls to review each session. Intervention fidelity was maintained and no deviations were identified. We will adhere to the same plan to maintain fidelity on the current study.

Data Collection & Participant Burden

Study visits typically lasted 3-5 hours and involved some unnecessary participant burden. Based on feasibility data and participant feedback, data collection has been streamlined for the current study. Moving forward, we have opted to remove sleep actigraphy and several interview/self-report measures which lacked clinical utility or were redundant. In addition, we will not collect dark-enhanced startle/heart rate variability data and inflammatory markers at mid-point (Time-2 Visit), primarily to decrease participant burden.

Feasibility of Measuring Immunologic and Psychophysiological Biomarkers as Outcomes

In Project Stress-Less, participants' willingness and tolerance of blood collection and dark-enhanced startle/heart rate variability assessments were greater than anticipated. We had hypothesized that the hyperarousal associated with PTSD might pose potential technical and data interpretation challenges, however very few participants declined or discontinued these procedures. We have established stream-lined data collection processes, and the technical difficulties we encountered with dark-enhanced startle/heart rate variability data collection have been resolved with the purchase of new equipment and more experienced staff.

Preliminary Effectiveness Results

Though not an aim of the pilot study, we analyzed available data to explore the effectiveness of TC-TSY compared to CPT-C. These analyses suggest, descriptively, that the TC-TSY group experienced improvements in PTSD severity, depression, pain levels, and acoustic startle response, from baseline to two- week post intervention. The TC-TSY group had: 1) **a reduction in self-reported PTSD symptoms**; 2) **a reduction in clinician assessed PTSD (CAPS)**; 3) **substantial decreases in pain** 4) **distinct decreases in depressive symptoms**. The TC-TSY group showed notable decreases in acoustic startle response (dark- enhanced startle) while the CPT-C group's startle response increased. Data indicate that TC-TSY as compared to CPT-C may represent an effective means of reducing the fear-related sequelae of trauma exposure as evidenced by psychophysiological indices, i.e. dark-enhanced startle and heart rate variability.

This will be further explored in the current RCT with enhanced recruitment and statistical power.

C. STUDY DESIGN AND METHODS

COVID-19 Contingency Plan (Atlanta and Portland)

As a result of the COVID-19 pandemic, local directive (3/12/2020) suspended study intervention visits for this study and national (3/17/2020) directive suspended all non-essential, in-person visits until further notice. Thus, we have modified the study design and methods to conduct group interventions and data collection virtually when possible. All interview-based assessments will be conducted via a suitable/authorized remote conferencing service between participant and research coordinator, i.e. Zoom Pro. All group intervention sessions will be conducted via a suitable/authorized remote conferencing service with group facilitators and group participants, i.e. Zoom Pro. Participant self-report will be entered by participant via direct entry of fully de-identified data into a secure REDCap web-based application. In the event self-report and/or interview-based assessments cannot be conducted via remote/virtual methods, the assessments will be administered via telephone. Informed consent and HIPAA authorization will be emailed and mailed to potential participants and reviewed via remote conferencing service or telephone. Once signed consent has been received by research staff, data collection visits and interventions will be conducted in the aforementioned manner. We will suspend collection of in-person physiological data at the Atlanta site. Portland site will utilize mail and drop off/ pick up for data collection kits such as portable KardioScreen ECG machines finger-stick blood collection method, portable BodyGuard HRV devices (Atlanta and Portland). Devices will be cleaned/sterilized in concordance with manufacturer's instructions and CDC guidelines.

Setting and Location (Atlanta)

This is a multi-site study with Atlanta VA Health Care System as the Coordinating Center. Study procedures in Atlanta will be conducted at the VA Clinical Studies Center. The Acoustic Startle Lab and the Atlanta VA Trauma Recovery Program located at the Henderson Mill Annex will also be used for intervention and data collection visits.

Clinical Studies Center (CSC)

The CSC is a professional center designated to facilitate human subjects' research at the Atlanta VA Medical Center and includes a laboratory (equipped with centrifuge, freezer, and biosafety hood), phlebotomy services for participants, and exam rooms to conduct study visits. The study team will utilize these services throughout the study.

Acoustic Startle Lab

The Acoustic Startle Lab is conveniently located on the fifth floor of the main VA. This space is dedicated to the collection of psychophysiological data (acoustic startle, HRV, skin conductance) and includes a Biopac System, sound attenuating audiology booth and control room for staff.

Trauma Recovery Program

The Trauma Recovery Program is located on the fourth floor of a leased VA space in the Henderson Mill Annex (2296 Henderson Mill Road) and contains ample parking, office space and group rooms. Study procedures including informed consent, self-report measures, interview-based assessments and dark psychophysiological data collection will also be conducted in a dedicated research office within the TRP.

Additionally, the yoga and CPT group sessions will be conducted in the TRP group rooms.

Setting and Location (Portland)

Study procedures will be conducted at the VA Portland Health Care System at both the Portland and Vancouver VA Medical Centers. Specifically, intervention sessions (Yoga and CPT) will be held at the VAPHCS Vancouver, WA campus and data collection visits will be conducted at the main VAPHCS campus in Portland, OR. Once IRB approval is received, space will be designated for data collection and intervention sessions for the duration of this study.

Participant Selection

Inclusion Criteria

1) Women Veterans who experienced military sexual trauma;
2) Meets DSM-V criteria for PTSD due to military sexual trauma
3) Difficulty falling asleep or difficulty staying asleep (insomnia)
4) Ability to give informed consent;
5) Willing to participate in either TC-TSY or CPT;
6) Available to attend the study intervention at the scheduled times.

Exclusion Criteria

1) Diagnosis of schizophrenia with significant psychotic symptoms (determined via clinician interview);
2) Current, active suicidal intent or plan (determined via clinician interview);
3) Current severe alcohol substance use disorder (determined via clinician interview);
4) Medical conditions that can contribute significantly to psychiatric symptoms, including poorly controlled hypo/hyperthyroidism, kidney or liver failure (determined via review of medical record);
5) Dementia (determined via review of medical record);
6) Moderate or severe traumatic brain injury (TBI) or other cognitive impairment sufficient to interfere with ability to give informed consent (determined via review of medical record);
7) Pain due to acute injury (<3 months), post-surgical pain (<3 months) or pain due to malignancy (determined through Veteran self-report and/or medical record);
8) Receiving mental health trauma focused therapy (TFT) treatment outside of the VA (determined through Veteran self-report);
9) Current engagement in trauma-focused treatment, yoga practice or other treatment/intervention at odds with the study intervention.

Recruitment (Atlanta)

Veterans will be recruited in the following ways:

1) Veterans may self-refer from flyers and brochures located in waiting areas and bulletin boards located in various clinics within the VA including the TRP, Women's Wellness and Primary Care. Flyers may also be distributed during VA sponsored events (For Example: Vet Fest, Heart Health events, VA Research Day). Following initial contact, a phone screen and chart review will be completed to assess

eligibility.

2) Clinicians within the TRP, Women's Wellness and Primary Care, and any other out-patient clinic or community-based out-patient clinic (CBOC) may also refer patients to the study. Veterans will be given the option to contact the study team directly or they may give consent to be contacted by a member of the study team. A brochure with an overview of study information and contact information may be provided to the interested Veteran by the clinician. Following initial contact, a phone screen and chart review will be completed to assess eligibility.
3) Study staff will conduct ongoing chart review of patients who have been referred to TRP for services, and/or who have recently completed TRP intake. The study team will contact TRP clinicians regarding any patient who could be a potential candidate for the study. At that time, the TRP provider will discuss the study with the Veteran and if the Veteran expresses interest, TRP staff will provide study contact information or facilitate an introduction to the study team. The study team and TRP providers will work closely to make sure that anyone who is interested will be screened for the study.
4) Study staff will attend TRP psychoeducational groups and staff meetings to present the study. Flyers with study contact information will be provided so that anyone who is interested may contact the team directly.
5) Study staff will conduct pre-screening of selected out-patient clinics within the Atlanta VAHCS to identify potential participants, for example, Women's Wellness. We have obtained a partial HIPAA waiver for this purpose. The clinical providers of potentially eligible participants will be contacted to request that they inform the patient of the study.
6) Recruitment will also be done outside of the VA setting. Study staff will place flyers in clinics, centers, college campuses, and other locations in the community which have the potential to reach women Veterans. Study staff will get consent from these locations prior to posting the flyers. In addition, study staff will attend community events geared towards Veterans in order to explain the research study to providers and provide information to those who may be interested. Note: The study team will not be recruiting non-Veterans into the study, but will expand recruitment efforts outside the VA setting.
7) To supplement provider referrals, we may also conduct initial outreach to potentially eligible participants via recruitment letters, as required by the IRB. In addition to patients identified via pre-screening, we propose to use the VA Informatics and Computing Infrastructure (VINCI) databases to identify patients at the Atlanta VA Health Care System who may be eligible for participation in the research study. We will ask the VINCI data managers to pull requested data from the Corporate Data Warehouse (CDW). The data pulled from VINCI will include patient names, phone numbers and address, dates, social security number, visit and hospital information, and other health information. This information will be necessary to recruit participants, and a partial HIPAA authorization waiver has been obtained. These identified data will be directly transferred electronically from the VINCI environment to the secured research server VHAATGFPC10 located in the OIT server room at the facility. This will be done by Christine Jasien, Atlanta VAMC statistician. The servers are managed by the Atlanta and Region 3 OIT offices and security is managed by the Information Security Officers at that facility. The server room has appropriate security controls in place (including a locked room, password protection, and encryption), and the drive that the data will reside at will be controlled so that only the research team

has access to the data. Patients identified for contact will be sent an IRB-approved letter referring to the study as "Project Stress-Less" for privacy purposes. The study team will discuss the study with interested patients by telephone, providing further information about the study.

The PI has been granted a partial HIPAA waiver in order to conduct these pre-screening/recruitment activities which facilitate identification of eligible participants. Recruitment rates will be calculated by comparing the total number of patients screened for the study to the number of patients who provide informed consent to participate.

Recruitment (Portland)

Veterans will be recruited in the following ways:

1) Veterans may self-refer from flyers and brochures located in waiting areas and bulletin boards located in various clinics within the VAPORHCS including the Mental Health Clinic (MHC), Women Veteran Health Clinic (WVHC), PTSD Clinical Team (PCT), and Primary Care Mental Health Integration (PCMHI). Following Veteran initiated contact, a phone screen and chart review will be completed to assess eligibility.
2) Clinicians within participating mental health clinics and any other out-patient clinics (CBOC) may also refer patients to the study. Veterans that are identified by treatment providers (who have pre-existing relationships with the potential participant), meet criteria, and are interested in participation will be provided a verbal explanation and printed IRB-approved study flyer from that provider who also will collect verbal consent to be contacted by a study member. At that time, Veterans will be given take home information about the study purpose and details. Consent of potential participants to be contacted by a study team member will prompt the treatment provider to refer the Veteran by first and last name along with last 4 digits of the Veteran's SSN to the study team either via encrypted email, phone, or by attaching a study team member as a cosigner to a CPRS note. Providers will document in CPRS the conversation with the Veteran and the Veteran having consented to be contacted for study participation. Following Veteran consent to be contacted, a study team member will follow up with a phone screen and chart review will be completed to assess eligibility.
3) Study staff will conduct pre-screening of selected out-patient clinics within the VAPORHCS to identify potential participants. In cases where a potential participant was not identified as such by other recruitment efforts described in this waiver (e.g. via their treating clinicians or flyers), under the principle of beneficence, the study should be made available to them. In those cases, a study team member will request permission from the clinician in charge of the clinic to reach out to the potential participant. If granted permission by the clinician in charge to contact the potential participant, a study team member will contact the potential participant. That contact will include a letter to the identified potential participant acknowledging PHI that assists the Veteran in understanding why they were identified for participation and will be signed by the treating clinician or clinician in charge. In addition, that contact will include a letter signed by a member of the study team introducing the study to the potential participant as a means for recruitment. This letter will not be individualized and will include a brief/general description of the study. The two letters included in this contact will be documented in the CPRS chart but included as a research related document that is not viewable to the Veteran.
4) Study staff will attend staff meetings of participating mental health clinics to

present the study. Flyers with study contact information will be provided so that anyone who is interested may contact the team directly.

5) Study staff will conduct pre-screening of selected out-patient clinics within the VAPORHCS to identify potential participants, for example, Women Veterans Health Clinic. We have requested a partial HIPAA waiver for this purpose. The clinical providers of potentially eligible participants will be contacted to request that they inform the patient of the study. Additionally, study staff will send opt-out letters to these identified potential participants as a means of recruitment (see Appendix 2).
6) Recruitment will also be done outside of the VA setting. Study staff will place flyers in clinics, centers, college campuses, and other locations in the community which have the potential to reach women Veterans. Study staff will get consent from these locations prior to posting the flyers. In addition, study staff will attend community events geared towards Veterans in order to explain the research study to providers and provide information to those who may be interested. Note: The study team will not be recruiting non-Veterans into the study but will expand recruitment efforts outside the VA setting.
7) To supplement provider referrals in Portland, we may also conduct initial outreach to potentially eligible participants via recruitment letters, as required by the IRB. In addition to patients identified via pre- screening, we propose to use the VA Informatics and Computing Infrastructure (VINCI) databases to identify patients at the VA Portland Health Care System who may be eligible for participation in the research study. We will ask the VINCI data managers to pull requested data from the Corporate Data Warehouse (CDW). The data pulled from VINCI will include patient names, phone numbers and address, dates, social security number, visit and hospital information, and other health information. This information will be necessary to recruit participants, and a partial HIPAA authorization waiver will be requested. These identified data will be directly transferred electronically from the VINCI environment to the secured research server VHAATGFPC10 located in the OIT server room at the facility then to the secure research folder at VAPORHCS. This will be done by Christine Jasien, Atlanta VAMC statistician. The servers are managed by the respective OIT offices and security is managed by the Information Security Officers at that facility. The server room has appropriate security controls in place (including a locked room, password protection, and encryption), and the drive that the data will reside at will be controlled so that only the research team has access to the data. Patients identified for contact will be sent an IRB-approved letter referring to the study as “Project Stress-Less” for privacy purposes. The study team will discuss the study with interested patients by telephone, providing further information about the study.

The PI will request a partial HIPAA waiver in order to conduct these pre-screening/recruitment activities which facilitate identification of eligible participants. Recruitment rates will be calculated by comparing the total number of patients screened for the study to the number of patients who provide informed consent to participate.

Methods

Data Collection *(See Table 1: Study Flowchart and Data Collection Schedule)*

Participants will be assessed at five time-points during the study. Following the phone pre-

screening interview, the participant will be scheduled for the Screening/Consent Visit (Time 0) for further determination of eligibility. If eligible, participants will be scheduled for the Baseline/Randomization Visit (Time 1) followed by the Mid- Treatment Visit (Time 2), the 2-Week Post Treatment Visit (Time 3) and the 3-Month Follow-Up Visit (Time 4). Each study visit will last 2-3 hours and includes collection of self-report measures, interview-based assessments, psychophysiological assessment, and immunological measures. Finally, if the participant completes intervention and elects to participate in the crossover intervention, they will be asked to complete several self-report measures (PCL, BDI, BPI, PSQI, VR-12 & Treatment Evaluation Form) before treatment, midway through treatment and again 2-weeks and 3-months post treatment. Participants opting to do the crossover intervention will not be compensated and will be given the option to complete the measures via phone.

Self-Report and Interview-Based Assessments

Participant self-report and interview-based assessments will be administered on paper forms or via direct entry of fully de-identified data into the VA REDCap web-based application. Various self-report forms and interviews will be administered at each study visit.

The following self-report and interview-based measures will be used for patient assessment during the course of the study:

Medical Outcomes

- Adverse Events will be assessed at each time-point.
- Concomitant Medications will be assessed at the Baseline/Randomization Visit and any changes will be documented at follow-up visits.
- VA Group Engagement will be assessed at all timepoints to determine if the Veteran is engaged in other groups and if so, which groups and how frequently they attend.

Demographics

- A Basic Demographics form will be administered at the screening visit and updates to demographics and contact information will be assessed at each follow-up visit.
- Currently enrolled participants that have consented to additional demographic survey questions, will be administered a sexual orientation and gender identify (SOGI) survey. New participants enrolled will be administered the SOGI survey during the demographics portion of the screening visit.
-

Trauma Exposure:

- The Deployment Risk and Resilience Inventory-2[78] (DRRI-2) was prepared with support from the Department of Veterans Affairs and contains questions regarding experiences before, during and after deployment. For purposes of this study, Section D (Combat Experiences) will be administered to assess level of combat exposure.
- The Childhood Trauma Questionnaire (CTQ) assesses exposure to and severity of childhood trauma across five subscales including: physical neglect, physical abuse, emotional neglect, emotional abuse and sexual abuse.
- The Life Events Checklist (DSM-5 version) is a self-report measure with established psychometrics designed to screen for potentially traumatic events in a respondent's lifetime and has been used with Veterans[79].

PTSD Severity:

- The Clinician Administered PTSD Scale (CAPS-5) is the gold-standard for assessing PTSD. The CAPS uses a clinician-administered diagnostic instrument that assesses lifetime and current PTSD diagnosis and symptoms and global PTSD symptom severity[80]. The CAPS also yields a continuous measure of the severity of both overall PTSD and of the four PTSD symptom clusters. It assesses the validity of the participant's responses and has excellent psychometric properties. The Past-Month version will be administered at Baseline and the Past Week version will be administered at each of the follow-up visits. CAPS-5 interviews will be audio-recorded for quality assurance purposes.
- The PTSD Checklist (PCL-5) is a 20-item self-report rating-scale instrument that parallels diagnostic criteria for PTSD, as delineated in the Diagnostic and Statistical Manual of Mental Disorders (DSM-V) [81]. The PCL is the standard measure of PTSD symptoms in the VA and can be used to determine PTSD diagnosis and PTSD symptom severity.

Psychiatric Symptoms/Diagnoses:

- The MINI International Neuropsychiatric Interview-Version[82] (MINI) 7.0.0 will be administered to assess participant's mental health symptoms/diagnoses according to DSM-V criteria. MINI Interviews will be audio recorded for quality assurance purposes.
- The Beck Depression Inventory-II (BDI-II) is a 21-item psychometrically sound self-report rating scale of the common symptoms of depression and the severity of these symptoms[83].
- The Difficulties in Emotion Regulation Scale (DERS) is a brief, 36-item, self-report questionnaire designed to assess multiple aspects of emotion dysregulation. The measure yields a total score as well as scores on six scales derived through factor analysis.
- The Dissociative Experiences Scale (DES) is a 28-question self-report form developed as a screening tool for Dissociative Identity Disorder.

Symptoms:

- The Brief Pain Inventory[84] is a widely used measurement tool for assessing pain severity and pain interference.
- The Pittsburgh Sleep Quality Index (PSQI) is a self-report scale consisting of 19 items that produce a global sleep quality score and the following seven component scores: sleep quality, sleep latency, sleep duration, habitual sleep efficiency, sleep disturbance, use of sleeping medications, and daytime dysfunction[85]. The PSQI has established internal consistency and reliability in various clinical populations of women (Cronbach alphas 0.80 across groups).
- The Berlin Questionnaire[86] for sleep obstructive apnea is a 10-item questionnaire well-known for its accuracy in predicting the presence of sleep apnea in patients. It has established sensitivity and specificity (Cronbach alphas 0.86 and 0.77, respectively, when polysomnography was used for confirmation).
- The Epworth Sleepiness Scale[87] is an 8-item self-report measure which provides a measure of a general level of daytime sleepiness, or average sleep propensity in daily life. It is widely used for making this assessment.
- The Patient Health Questionnaire-15 (PHQ-15) is a 15 item scale that inquires about somatic symptoms or symptom clusters that account for more than 90% of the physical complaints reported in the outpatient setting and include 14 of the 15 most

prevalent DSM-V somatization disorder somatic symptoms[88]. It has demonstrated internal consistency and convergent validity with the BDI ($r = 0.559; p < 0.01$).

Social Functioning and Quality of Life:

- The Veterans RAND 12 Item Health Survey[89] (VR-12) is a 12-item instrument used to measure health related quality of life and will be administered at various time-points.
- The PROMIS® instruments use modern measurement theory to reliably and validly assess patient– reported outcomes (PROs) for clinical research and practice. The PROMIS ® short forms for social functioning will be used in the proposed study.

Sexual Orientation and Gender Identity (SOGI):

- In the current study, we will expand data collection of demographic variables to include sexual orientation and gender identity based on current recommendations for inclusive research. Currently enrolled participants in the study will be asked to verbally consent to participate in a follow-up phone visit to complete a demographic survey lasting no more than 15 minutes. This follow-up survey will include pertinent demographic items including sexual orientation and gender identity. Research team members will review the electronic medical record of the enrolled participants to gather their most current phone number and address. Currently enrolled participants will then be contacted by phone. If the participants are unable to be reached by phone within 10 days of first contact, a letter will be sent to the last known address of the participant. This letter will provide information regarding the follow-up visit and how to complete the visit or opt-out of the visit. All future participants will complete the SOGI demographic survey as part of their initial visit and will not require an additional follow-up phone visit.

Dark Enhanced Startle Assessment (Atlanta Only)

Dark enhanced startle response will be assessed using a paradigm in which acoustic startle (blink) response is assessed in darkness versus light conditions. Data will be collected by trained research staff using Biopac MP150 for Windows. The acquired data will be processed using Mindware software and then exported to Excel for analyses. Dark enhanced startle data will be collected at Baseline, 2-Weeks Post and 3-Months Post Treatment.

Heart Rate Variability Assessment

In order to measure heart rate variability, we will utilize the Firstbeat Bodyguard-2[90] device which is a reliable R-R interval recording device for short and long-term measurements. The device is lightweight and user friendly. The device is attached directly to the skin with two chest electrodes and begins recording data

automatically. Participants will wear the device for up to 48-hours. Data will be downloaded directly to Firstbeat Analysis Server or SPORTS software for analysis. This data will be used to monitor changes in heartbeat due to stress.

Cardiovascular Disease Assessment (CVD)

In order to screen for CVD, a 12-lead ECG will be conducted at the Baseline and Time 3 Visits by trained study staff. Results will be interpreted by Dr. Amit Shah, a co-investigator and cardiologist at the Atlanta VAMC and will be analyzed to evaluate the relationship between heart health and PTSD diagnosis/severity. Additionally, there are some ECG markers that are autonomic in nature (QT interval) and TC-TSY could positively influence this measure.

Polysomnogram (Atlanta Only)

Participants with moderate to high risk scores on the Berlin questionnaire and the Epworth Sleepiness Scale will be given the option of participating in a sub-study which involves taking part in an overnight sleep study (polysomnogram) at the Atlanta VA Sleep Lab. This will yield information regarding the prevalence of OSA and other sleep disorders in this sample of women Veterans as well as an objective measure of sleep quality. Participants who consent will be asked to complete an overnight sleep study at any point during their participation in the study. A stand-alone consent and HIPAA form will be provided to eligible, interested participants.

Immunological Measures (IL-6, IL-10, C-Reactive Protein)

Trained study personnel, CSC phlebotomists and VA Lab phlebotomists will perform blood draws. Blood samples of 25mL will be obtained using EDTA-containing (purple top) tubes. Tubes will be spun in a centrifuge and plasma will be pipetted into cryovials which will be stored in a freezer located in a VA laboratory. Trained study personnel will be responsible for processing the samples, ensuring proper storage and sample tracking. Samples will be labeled with the subject identification number and visit number. No identifiers will be included in the sample label. Plasma levels of IL-6, IL-10, and C-Reactive Protein will be obtained at Baseline, 2-Weeks Post and 3-Months Post Treatment.

Table 1: Study Flowchart and Data Collection Schedule

	Pre-Screen Phase	Screening Visit[a]	Baseline/ Random-ization Visit	Mid-Tx Visit	2-Week Post Tx Visit	3-Month Follow-Up Visit	Crossover Intervention[d] or subsequent survey
		Time-0	Time-1	Time-2	Time-3	Time-4	
Phone Screening	X						
Chart Review/Med. Clearance	X	X					
Inclusion/Exclusion Review	X	X					
Informed Consent/HIPAA		X					
AEs/Con Meds/VA Groups Log		X	X	X	X	X	
Basic Demographics/ Updates		X	X	X	X	X	X
DRRI-2 Combat Experiences		X					
Childhood Trauma Ques.		X					
Life Events Checklist		X					
MINI (for DSM-5)		X					
Berlin Questionnaire		X				X	
Epworth Sleepiness Scale		X				X	
**RANDOMIZATION**			X[b]				
Beck Depression Inventory			X	X	X	X	X
Difficulties in Emotion Regulation Scale			X	X	X	X	
Dissociative Experiences Scale			X	X	X	X	
Brief Pain Inventory			X	X	X	X	X
Pittsburgh Sleep Quality Index		X	X	X	X	X	X
Patient Health Questionnaire			X	X	X	X	
PROMIS Measures			X	X	X	X	
PROMIS Social Isolation							X
CAPS-5			X	X	X	X	
PTSD (PCL-5)							X
Chronic Pain Survey (BPI)							X
PTSD-Checklist-5		X	X	X	X	X	X
Intervention Tracking				X	X	X	X

VR-12			X	X	X	X	X
12-Lead ECG			X		X		
Dark-Enhanced Startle Session[e]			X		X	X	
Heart Rate Variability Measure			X		X	X	
Blood Draw/ Processing			X		X	X	
Provide Compensation		X	X	X	X	X	
Offer alternate intervention						X	
Polysomnogram (optional)		X[c,e]					

[a]Given the nature of this study, the T-0 and T1 Visits may be combined into one visit

[b]Randomization will occur after all other Baseline/Randomization Visit procedures have been completed

[c]The sub-study will be described at the screening visit and if interested, consent will be obtained for eligible participants. The PSG can be completed at any time during study participation

[d]If the participant elects to do the crossover intervention, measures will be administered by phone or in-person at baseline, mid-treatment and at 2-weeks and 3-months post treatment e Given the availability of equipment, this measure will only be conducted for participants in Atlanta

Informed Consent Process

Informed consent in Atlanta will be obtained by trained staff during the Screening/Consent Visit in a private room at the AVAMC Clinical Studies Center or the Trauma Recovery Program located at the Henderson Mill Annex. In Portland, informed consent will be obtained by trained staff during the Screening/Consent Visit in a private room at the VA Portland Health Care System at either the Portland or Vancouver VA Medical Center. Participants will be informed that they are being asked to participate in a research study. They will be told the nature of the procedures and the interventions, informed of the risks associated with their participation, asked to read the consent form, and encouraged to ask questions or discuss any pertinent issues. They will be told that declining to participate will not influence or compromise their care or employment at the either VAMC (if pertinent). Participants will be notified that the research project is voluntary, that participation can be stopped at any time and that participation will not impact any services or benefits they receive at the Atlanta or Portland VAMC. Participants will also be informed that they will be compensated for time and travel. All research volunteers will be asked to sign the consent form after they have read it and discussed the study with research personnel. Participants will be given as much time as needed to read the form thoroughly and have all questions addressed. Study personnel will ask questions to determine adequate comprehension of the study. Only participants who have given their written informed consent will participate. In addition to informed consent, subjects will read and sign a HIPAA authorization. Participants may withdraw their consent at any time and may withdraw from the study at any time.

Randomization

Once a participant has completed the Screening/Consent Visit and is found to be eligible to participate, they will be assigned a sequential study number which will be used to identify data. They will be scheduled for a Baseline/Randomization Visit prior to the start of study intervention. The study statistician, who is responsible for making and maintaining the study randomization schedule, will provide the project manager with the randomization assignment for each participant. Each participant’s assignment will be revealed to both study staff and participant at the completion of the Baseline/Randomization Visit. The participant will be given specific information regarding their treatment assignment and confirm availability for the upcoming sessions.

Study Intervention

Trauma Center-Trauma Sensitive Yoga

The yoga intervention consists of a standardized trauma-sensitive Hatha yoga protocol developed by Emerson, Spinazzola and colleagues for use with civilian women with treatment-resistant PTSD[63]. Hatha yoga is a general term to describe the practice of physical postures, breath work, and mindfulness—what is commonly referred to in the West simply as "yoga." There are many styles of Hatha yoga (e.g., Iyengar, vinyasa, Ashtanga, Bikram), but when the term Hatha yoga is used in the West in contrast to another specified style, it most often designates a more gentle, slower-paced approach to the physical postures. Our protocol uses this gentler, slower approach. It involves ten weekly 90-minute group sessions of Trauma Center-Trauma Sensitive Yoga. Sessions address themes related to establishing safety, individual choice, being in the present moment, and taking effective action. The intervention will be conducted by yoga teachers certified in Trauma Center- Trauma Sensitive Yoga. Study staff will provide participants in the yoga intervention with detailed information related to the sessions. In addition, instructional DVDs will be provided to participants in the yoga intervention to facilitate practice at home. For participants who do not have access to a DVD player, a link will be provided which will direct participants to the private online videos on YouTube. These videos are hidden from the public, therefore the only way to gain access is by entering the link provided by the study team. Finally, reminder calls will be made each week to ensure optimal group attendance. During follow-up visits, study staff will assess whether participant engaged in at-home TC-TSY practice, and the frequency of at-home practice (via use of DVD/online videos, breathing techniques etc.) using an intervention tracking form.

Cognitive Processing Therapy-Cognitive

The control group, Cognitive Processing Therapy (CPT), is one of the gold-standard therapies for PTSD treatment in the VA mental health system. CPT is a cognitively-based, trauma focused treatment which will be led by two licensed clinical social workers who work in the Women's Trauma Program. As per the treatment protocol, there are twelve 90-minute weekly sessions. The group sessions focus on identifying how thoughts change as a result of trauma exposure and ways in which to realistically evaluate these maladaptive thoughts and come up with more accurate alternative thoughts. Also per protocol, group members do not share their specific trauma details in group. These sessions will be conducted concurrently with the yoga intervention throughout the course of the study. Study staff will provide participants in the CPT group with detailed information related to the sessions. Reminder calls will be made each week to ensure optimal group attendance. During follow-up visits, study staff will assess the degree to which participant engaged in homework assignments and used strategies learned in group using an intervention tracking form.

Compensation (Atlanta)

Participant compensation will be provided following each study visit and following each treatment session. Participants will receive $40 for completion of the Screening/Consent, Baseline/Randomization and Midpoint Visits. For completion of the 2-Week and 3-Month post-treatment visits, participants will receive $60. For each treatment session, participants will receive $15. Compensation will be sent in the form of a VA check or via direct deposit. Each method of payment will be discussed and participants will indicate their preference at the initial visit. Eligible participants who consent and complete the sub-study will receive $60 once they have completed the sleep study. The total possible

compensation for completion of the study is $480. In certain cases, if the participant has to return a second time to complete a study visit, they may receive additional compensation of up to $20. For visits when the participant receives the heart-rate variability monitor, compensation will be sent as soon as the device is returned to the study site. Participants taking part in the crossover intervention will not receive compensation for completion of the study forms.

Compensation (Portland)

Participant compensation will be provided following each study visit and following each treatment session. Participants will receive $40 for completion of the Screening/Consent, $60 for Mid-treatment and 3-Month Follow-up visit, and $85 for the Enrollment/Baseline/Randomization and 2-Week Post-Treatment Visits. For each treatment session, participants will receive $10 for which they attend. Compensation will be sent in the form of a VA check or via direct deposit. Each method of payment will be discussed, and participants will indicate their preference at the initial visit. The total possible compensation for completion of the study is $430-450. In certain cases, if the participant has to return a second time to complete a study visit, they may receive additional compensation of up to $20. For visits when the participant receives the heart-rate variability monitor, compensation will be sent as soon as the device is returned to the study site.

Participants taking part in the crossover intervention will not receive any compensation.

D. POTENTIAL RISKS/DISCOMFORTS TO STUDY PARTICIPANTS AND MEASURES TO PREVENT OCCURRENCE

Self-Report and Interview-Based Assessments

The assessments include questions about exposure to stressors and other topics that might produce transitory distress in some individuals. There is the potential for participants to experience embarrassment or other negative consequences if some of the experiences were disclosed, particularly if their identity was linked to interview data. We think that these risks are minimal given the protections in place to maintain confidentiality and the plans to respond to participants who experience distress.

Study Interventions

Trauma Center-Trauma Sensitive Yoga: There are physical risks in participating in the yoga intervention, as well as the potential for emotional distress during yoga sessions. The protocol has been designed specifically for women with PTSD. It is expected that some women will experience intense emotion during the yoga. The protocol has been designed to mitigate these risks and address physical and emotional distress if necessary.

Cognitive Processing Therapy-Cognitive: There is a risk for transitory psychological distress in participating in the control condition, cognitive processing psychotherapy. This intervention is evidence-based and manualized and some psychological distress is expected. Clinicians providing this intervention are highly trained to respond to any such distress.

Venipuncture

The blood draw may cause mild discomfort, bruising, redness, or swelling. Study staff will discuss the process of having blood drawn with the participant to determine if there are any concerns or past difficulties with having blood drawn. Pertinent information will be relayed to

the phlebotomist beforehand. These procedures are only done by skilled personnel in order to reduce the risk of any negative reactions. The risks associated with the blood draw are unlikely and, if they occur, should resolve within a few days.

<u>Psychophysiological Data Collection</u>
Participants may experience mild discomfort or distress during the psychophysiological assessment in response to being in the booth during the session which involves periods of light and dark. Procedures will be explained fully and participants will be instructed to end the session at any time if they feel any discomfort or stress.

<u>Heart Rate Variability Data Collection</u>
Participants may experience redness or itching at the site where the electrodes for the Bodyguard are placed (clavicle and ribcage). These risks are unlikely, and if they occur, should resolve within a few days.

<u>Electrocardiogram</u>
Participants may experience temporary irritation/discomfort from the ECG electrodes on the skin.

<u>Loss of Confidentiality</u>
In order to protect against the risk of a breach in confidentiality, study data will be stored in locked filing cabinets within a locked research office that can only by accessed by research personnel. No names or identifying information will be used in publications that result from this study. Identifying information will not be released to any outside party (beyond those connected to the study) without written consent from the participant. An electronic dataset that includes identifiers linked to the subject identification code will be stored in a password-protected database that is located on a secure LAN within our locked research suite. Hard copies of forms that include identifiers (e.g., signed informed consent forms) will be kept in a secure, locked file cabinet within a locked VA research office.

E. BENEFITS

There are potential benefits to participants. Trauma Center-Trauma Sensitive Yoga and Cognitive Processing Therapy-Cognitive are both treatments that have the potential to improve PTSD symptoms, sleep, and pain. They will be exposed to a therapeutic activity that they can continue outside of sessions if they choose.
Participants will also be offered the intervention to which they were not randomly assigned following their completion of the study intervention and data collection, e.g. those assigned to CPT will be offered Trauma Center-Trauma Sensitive Yoga after completion of the study.
Participants in this study will be involved in a process that may provide future benefit to other MST-exposed female Veterans with PTSD. The potential benefits to others include knowledge development necessary to implement this intervention throughout the VA if it is successful in treating PTSD, chronic pain and insomnia in female Veteran sexual assault survivors.

The knowledge to be gained in this study is scientifically essential to determine the effectiveness of the experimental intervention. This study has the potential to impact PTSD treatment at the national level of the VA, which is the largest health care system in the United States. This RCT could yield meaningful data to support clinical guidelines for a complementary and alternative medicine intervention which could be disseminated to and implemented in VA Medical Centers nationwide, and thus improve the quality of care for

female Veterans with PTSD.

F. DATA MONITORING AND ANALYSIS

Data Management

As part of this protocol, we will collect data via chart review, self-report form, interview-based assessment, blood collection, heart-rate variability device, ECG report, polysomnogram and dark-enhanced startle testing (Atlanta only). Data will either be collected at the AVAMC Clinical Studies Center or the Trauma Recovery Program or Portland VA or Vancouver, WA VA location of the VAPORHCS and then subsequently transported back to respective VAMC for storage. Any sensitive data including consent forms, session sign-in sheets etc. will be transported in a blue lock-bag as outlined in the approved VA Research Data Inventory (Privacy and Data Security Section). Data will be captured, stored and processed in the following ways:

Chart Review

Data will be extracted from the VA medical record including diagnoses, active medications, and reportable adverse events (if applicable). These data will be added to the VA REDCap database.

Self-Report and Interview Data: Self-report and interview data will be captured through pen and paper measures or through direct data entry into the VA REDCap (Research Electronic Data Capture) project database using a VA-approved tablet. REDCap[89] is a secure, web-based application designed to support data capture for research studies. REDCap for VA is installed and accessible only behind the VA firewall and data is backed-up nightly and every 6 hours. In addition, VA REDCap provides data de-identification features and restricts access to PHI at the user-level. Deidentified self-report and interview data will be securely exported from VA REDCap to Excel or SPSS and then uploaded to the VA password-protected research drive for analysis as needed. Periodically, deidentified self-report and interview data will be submitted to the study statistician for data analysis and review.

Additionally, demographic information, including identifiers (study ID), for each participant will be stored in the REDCap database that is password-protected and accessible only by approved members of the research team. The research suite is locked at all times with access granted only to authorized personnel. Data residing on VA desktop computers are routinely backed up on a secure, off-site server. Thus, all data are protected from loss and are stored devoid of all patient identifiers.

Cytokine Data: In Atlanta, blood samples will be collected by trained study staff or CSC staff in the Atlanta VA Clinical Studies Center and labelled with date, study ID, and visit number only. Initial sample processing will take place in the CSC lab by trained staff before being sent to another lab within the VA for final analysis. Any sample remaining after initial analysis will be stored until the end of the study at which point remaining specimen will be destroyed. Deidentified cytokine data will be sent from the lab to the project manager. These data will be stored on the secure VA research server and the VA REDCap database until being sent to the Emory statistician for analysis. In Portland, blood samples will be collected by trained study staff in the VAPORHCS and labelled with date, study ID and visit number only. Initial sample processing will take place in the Loftis Lab at the VAPORHCS prior to shipment to the CSC of the AVAHCS for analysis. Any sample remaining after initial processing will be stored until the end of the study at which point remaining specimens will be destroyed.

Heart Rate Variability (HRV) Data: At both sites. HRV data will be captured using the portable Bodyguard device which participants will wear for up to 48 hours. In Atlanta, once the device is returned to study staff, deidentified HRV data from the USB device will be downloaded to either an Emory laptop or VA computer that belongs to the PI and contains data analysis software. The data will then be analyzed by trained study staff and exported to Excel. Periodically, the data will be retrieved from the laptop and uploaded to Box.net. Dr. Amit Shah (Cardiologist/Co-Investigator) will assist the study staff with troubleshooting any issues and address any questions that arise during the data analysis process. Dr. Shah will process the de-identified HRV data on his Emory workstation for generation of heart rate variability metrics. Data, although de-identified, will be transferred via HIPAA compliant methods, including encrypted VA-issued USB

flash drive, and Box.net.

In Portland, HRV data will be captured using the portable Bodyguard device which participants will wear for up to 48 hours. Once the device is returned to study staff, fully de-identified HRV data from the USB device will be downloaded to a VA computer and saved on the secured research folder designated for this study (\\R01PORHSM03.r01.med.va.gov\Research\Zaccari Research\Project Stress Less II) to which Dr. Amit Shah will have access for the purpose of data analysis.

Electrocardiogram Data: In Atlanta, 12-Lead ECGs will be completed by trained study staff using an IRB approved ECG machine. Paper ECG reports coded with the SID will be printed, stored in the participant binder and scanned to the secure VA research server where they will be reviewed periodically by Dr. Amit Shah (Cardiologist/Co-Investigator). These reports will contain the subject identification number and time-point, but no protected health information. Periodically, these data will be saved to a VA-issued USB drive and uploaded to the secure research drive and VA REDCap database. Dr. Shah will process the de-identified ECG data on his Emory workstation for generation of repolarization metrics. Data, although de-identified, will be transferred via HIPAA compliant methods, including encrypted VA-issued USB flash drive, and Box.net.

In Portland, 12-Lead ECGs will be completed by trained study staff using an IRB approved ECG machine. Paper ECG reports coded with the SID will be printed, stored in the participant binder and scanned to the secure VA research server. These reports will contain the subject identification number and time-point, but no protected health information. The PI will have access to these original data to provide to Dr. Amit Shah (cardiologist/CO-Investigator) for analysis.

Polysomnogram Data (Atlanta Only)
Polysomnogram results will be extracted from the VA medical record, recorded onto a form, and entered into the VA REDCap database.

Psychophysiological Data (Dark-Enhanced Startle [Atlanta Only])
Psychophysiological data will be captured on a laptop containing Biopac software. These deidentified data will be analyzed using Mindware software and then exported to Excel. These data will be stored on the VA research drive.

Data Quality
All data will be examined for completeness and an assessment of missing data will be performed to check for any biases which may occur. Data will be monitored and entered promptly in order to detect issues as they arise. Special attention will be paid to note what, if any, demographic and clinical factors may be related to subject attrition, retention and adherence. Study retention refers to those who complete all study-related assessments. Dose-effects will be accounted for based on the number of treatment sessions attended by each subject relative to their assigned group. Analyses will be performed to determine what if any covariates may be predictive of missing data over time and these variables will be adjusted for in subsequent models[90]. Close attention will be paid to underlying normality assumptions. Numerical transformations (such as square root, log, and inverse functions) may be applied to correct for deviations due to skewness.

Power Analysis
Given the estimated final sample size of 104 (52 per group) after expected levels of attrition (50% attrition from 210), we will be powered at 80% to detect moderate effect sizes for any

differences between the groups (two- group independent t-tests effect size (Cohen's d) of 0.555 and chi-square tests for differences in proportions between the two-groups effect size (Cohen's omega, ω) 0.274 and higher) as well as moderate-to-large effect sizes detected for differences between the groups over time (repeated measures group-by-time interaction effects: effect size of 0.28, Cohen's f) will be detected. Power analyses will be completed using PASS v.13.0.8[91].

Statistical Analysis

Comparisons between the groups at baseline will be run using t-tests, Mann Whitney non-parametric tests, and chi-square tests as appropriate. However, when numerical transformations are not sufficient, non-parametric and generalized non-normal response functions may also be employed. Multilevel mixed models (MLM) will be used instead of repeated-measures analysis of variance (RM-ANOVA), to analyze the differences between the groups over time. As opposed to RM-ANOVA which assumes independence between time points and assumes complete data for all subjects at all time-points, MLM adjusts for attrition (missing data) over time and applies appropriate correlation structure between the time points[92]. Multilevel modeling also provides insight into the variance components of the outcomes relative to those related to within-subject variability over time and those related to between-subject differences. These variance components are important for understanding the utility of each measure relative to reliably assessing each outcome. Generalized functions of these MLM models may also be run for non-normal outcomes due to highly skewed or zero-inflated data (common with biomarker and some behavioral measures) or binary outcomes (e.g. diagnosis determinations). These statistical modeling approaches will be applied appropriately to the outcome measures of interest for each of the three research aims. SPSS Version 22.0 will be used for all statistical analyses.

G. TRAINING

All study team members have completed the web-based Collaborative IRB Training Initiative (CITI) Program in the Protection of Human Subjects Research. The Project Manager will ensure that all study team members stay current with all required Emory and VA research trainings.

In addition, for all study team members working directly with participants, trainings will be conducted on the administration of structured interviews (CAPS, MINI), informed consent process, psychophysiological data collection, ECG, HRV data collection, phlebotomy (if applicable) and specimen processing. Once the staff member has met all training requirements for each item, they can begin working with study participants.

A detailed log of all study-related trainings will be maintained throughout the study.

H. DATA AND SAFETY MONITORING PLAN

Data and Safety Monitoring Plan (DSMP)

Safety Monitoring: The PI or anyone else who has contact with study participants during study activities have the responsibility to monitor for any potential adverse events and protocol deviations. Any potential participant for an adverse event will be reported immediately via "warm transfers" per VA guidelines at respective site and PI, who will contact the participant and determine if additional intervention is needed to ensure participant safety. Study personnel will be trained on these procedures, including contacting VA Police, on-call Mental Health providers Veterans Crisis Line and/or local

crisis lines, Protocol deviations will also be immediately reported to respective site PI who will ensure that adverse events deemed to be unanticipated problems and protocol deviations are properly reported to the IRB in a timely manner.
Detailed written documentation will be kept for all adverse events that occur over the course of the study. PI will hold regular meetings with study staff where they will discuss adverse events and protocol deviations associated with this project and ways to reduce repeat occurrences. Research staff will examine all cumulative adverse events quarterly to determine if there are any systematic problems and to implement protocol corrections as needed after receiving IRB approval.

Data Monitoring: All information linking study data to PHI will be kept within VHA electronically in secure computer files stored behind firewalls requiring password access, or in hardcopy form in locked file cabinets in locked offices. All patient identifiers will be removed prior to analysis. All investigators and team members who will have access to the data will have received appropriate background checks as part of hiring and/or credentialing and will have completed Data Security Training within the prior 12 months.

Per VHA guidelines, data resulting from this study will be stored locally on VHA password-secure folders. Requests for data access will be considered and responded to within one month of the request and datasets will be made available electronically. Requests must be made in writing to the study PI and provide information on the purpose for accessing the data.

All data used in final, published results will be made available for sharing. Published data will be available upon request to any investigator in order to enable independent validation and interpretation of published data.

Once the current study is closed, we will store de-identified, anonymized dataset in an approved data repository consistent with policies in 1200.12 (Use of Data and Data Repositories in Research). A sharing agreement will prohibit the recipient from identifying or re-identifying (or taking steps to identify or re- identifying (or taking steps to identify or re-identify) any individual whose data are included in the dataset.

Adverse Event Reporting

The principal investigator will be responsible for following adverse event reporting requirements as outlined below in the protocol. These responsibilities include: 1) reviewing the accuracy and completeness of all adverse events reported, 2) compliance with local IRB policies for reporting adverse events and/or serious adverse events, and 3) overseeing monitoring of research volunteers at each follow-up visit and as indicated by notification by the yoga teachers at each yoga intervention session. Relatedness involves an assessment of the degree of causality between the study intervention and the event. The PI will perform an assessment of relatedness, in conjunction with Dr. Skelton, who has significant experience in data safety monitoring. All AEs with a reasonable, causal relationship to the intervention will be considered “related.” A definite relationship does not need to be established.

AE and SAE Monitoring

For the proposed study, participants will be monitored at each data collection point and as indicated by notification from the yoga/CPT teachers at each intervention session.

Adverse events and serious adverse events will be assessed for relatedness to study participation and whether the event was anticipated or unanticipated. Those adverse events found to be unanticipated and related to study participation will be reported to IRB and VA R&D according to local reporting guidelines.

Safety Assessments

Safety assessments will be conducted with any study participant who reports new onset of: suicidal or homicidal thoughts, psychosis, substance use, or physical pain/symptom or physical injury that is determined to require medical evaluation beyond evaluation conducted within the TRP by Dr. Skelton or her designee.

Expedited Reporting of SAEs

Serious Adverse Events found to be related to participation in the research will be reported to IRB and Atlanta VA R&D according to local reporting requirements.

Collaborative Research

This protocol describes a multi-site study to be conducted at the AVAHCS and the VAPORHCS. AVAHCS is the coordinating center with VAPORHCS recently added as an additional site.

As the primary site/coordinating center, study staff of the AVAHCS will be responsible for equivalent study procedures described in this protocol but conducted in Atlanta. Additionally, AVAHCS study staff will be responsible for assistance in screening for the purposes of recruitment and the storage and analysis of biophysiological data that is collected from participants of VAPORHCS. Where applicable, these tasks have been described as to be conducted at AVAHCS. Project Management of the coordinating center is being conducted by Terri Haywood (Terri.Haywood@va.gov; 404-321-6111, ext. 207026).

Certificate of Confidentiality

N/A. The study does not include a Certificate of Confidentiality

Disclosure/Sharing

N/A. The study does not include disclosure/sharing outside the IRB-approved VAPORHCS and AVAHC study personnel.

References

1. Kimerling R, Street AE, Pavao J, et al. Military-related sexual trauma among Veterans Health Administration patients returning from Afghanistan and Iraq. *American Journal of Public Health.* August 1, 2010 2010;100(8):1409-1412.
2. Vogt DS, Pless AP, King LA, King DW. Deployment stressors, gender, and mental health outcomes among Gulf War I veterans. *Journal of Traumatic Stress.* 2005;18(2):115-127.
3. Zinzow HM, Grubaugh AL, Monnier J, Suffoletta-Maierle S, Frueh BC. Trauma among female veterans: A critical review. *Trauma, Violence, & Abuse.* 2007;8(4):384-400.
4. Khalsa SBS. Yoga as a therapeutic intervention: A bibliometric analysis of published research studies. *Indian Journal of Physiological Pharmacology.* 2004;48:269-285.
5. Telles S, Singh N, Joshi M, Balkrishna A. Post traumatic stress symptoms and heart rate variability in Bihar flood survivors following yoga: A randomized controlled study. *BMC Psychiatry.* 2010;10(18):1- 10.
6. Emerson DRSJ. Trauma-Sensitive Yoga : Principles, Practice, and Research. *International Journal of Yoga Therapy.* 2009;19:123-128.
7. van der Kolk B, Stone L, West J, et al. Yoga as an adjunctive treatment for posttraumatic stress disorder: A randomized controlled trial. *Journal of Clinical Psychiatry.* 2014;75(6):e559-e565.
8. Johnston J, Khalsa SB. Yoga as an intervention for post-traumatic stress disorder (PTSD) in military personnel. *International Journal of Yoga Therapy.* 2010(Supplement):28.
9. Groessl EJ, Weingart KR, Aschbacher K, Pada L, Baxi S. Yoga for Veterans with Chronic Low-Back Pain. *Journal of Alternative and Complementary Medicine.* 2008;14(9):1123-1129.
10. Kelly U, Dowling Evans D, Baker H. Yoga and posttraumatic stress disorder (PTSD) - Part I: A theoretical model and objective measures of yoga mechanisms of action *Complementary Therapies in Medicine.* 2014;under review.
11. Kelly U, Dowling Evans D, Baker H. Yoga and posttraumatic stress disorder (PTSD) - Part II: A systematic review and state of the science of the effectiveness of yoga and its components for PTSD. *Complementary Therapies in Medicine.* 2014;under review.
12. Ressler KJ, Mercer KB, Bradley B, et al. Post-traumatic stress disorder is associated with PACAP and the PAC1 receptor. *Nature.* 2011;470(7335):492-497.
13. Kamkwalala A, Norrholm SD, Poole JM, et al. Dark-enhanced startle responses and heart rate variability in a traumatized civilian sample: putative sex-specific correlates of posttraumatic stress disorder. *Psychosomatic Medicine.* 2012;74(2):153-159.
14. US Department of Veteran Affairs. *Veteran Population Projections Model (VetPop2014).* Washington, DC: National Center for Veterans Analysis and Statistics; November 7, 2014 2014.
15. United States Government Accountability Office. VA Health Care: Preliminary Findings on VA's Provision of Health Care Services to Women Veterans. 2009.
16. Kelly U, Skelton K, Patel M, Bradley B. More than MST: Complex Trauma and Complex Mental Health in Women Veterans. *Research in Nursing & Health.* 2011;34:457-467.
17. Foa EB, Keane TM, Friedman MJ, eds. *Effective treatments for PTSD: Practice guidelines from the International Society for Traumatic Stress Studies.* 2nd ed. New York: Guilford Press; 2009.
18. Kessler RC, Sonnega A, Bromet E, Hughes M, Nelson CB. Posttraumatic Stress Disorder in the National Comorbidity Survey. *Archives of General Psychiatry.* 1995;52(12):1048-1060.
19. Cloitre M, Stolbach BC, Herman JL, et al. A developmental approach to complex PTSD:

Childhood and adult cumulative trauma as predictors of symptom complexity. *Journal of Traumatic Stress.* 2009;22(5):399-408.

20. Cahill SP, Rothbaum BO, Resick PA, Folette VM. Cognitive-behavioral therapy for adults. In: Foa EB, Keane TM, Friedman MJ, eds. *Effective treatments for PTSD: Practice guidelines from the International Society for Traumatic Stress Studies*. 2nd ed. New York: Guilford Press; 2009.
21. Ready DJ, Pollack S, Rothbaum BO, Alarcon RD. Virtual reality exposure for veterans with posttraumatic stress disorder. *Journal of Aggression, Maltreatment & Trauma.* 2006;12(1/2):199-220.
22. Walser RD, Westrup D. *Acceptance & commitment therapy for the treatment of post-traumatic stress disorder: A practitioner's guide to using mindfulness & acceptance strategies.* Oakland, CA: New Harbinger Publications; 2007.
23. Schottenbauer MA, Glass CR, Arnkoff DB, Tendick V, Gray SH. Nonresponse and dropout rates in outcome studies on PTSD: Review and methodological considerations. *Psychiatry: Interpersonal and Biological Processes.* 2008;71(2):134-168.
24. Cloitre M, Stovall-McClough K, Nooner K, et al. Treatment for PTSD related to childhood abuse: a randomized controlled trial. *American Journal of Psychiatry.* 2010;167:915-924.
25. Coeytaux R, McDuffie J, Goode A, et al. Evidence Map of Yoga for High-Impact Conditions Affecting Veterans. 2014.
26. Strauss J, Coeytaux R, McDuffie J, Nagi A, Williams JJ. Efficacy of Complementary and Alternative Therapies for Posttraumatic Stress Disorder. 2011.
27. Libby DJ, Pilver CE, Desai RA. *Complementary and Alternative Medicine (CAM) in Specialized VA PTSD Treatment Programs.* Department of Veterans Affairs;2011.
28. Surís A, Lind L, Kashner TM, Borman PD, Petty F. Sexual Assault in Women Veterans: An Examination of PTSD Risk, Health Care Utilization, and Cost of Care. *Psychosomatic Medicine.* September 1, 2004 2004;66(5):749-756.
29. Cougle JR, Keough ME, Riccardi CJ, Sachs-Ericsson N. Anxiety disorders and suicidality in the National Comorbidity Survey-Replication. *Journal of Psychiatric Research.* Jun 2009;43:825-829.
30. U.S. Department of Veterans Affairs V. Women Veterans Health Workload Report, October 2010.. Washington, D.C.2010.
31. Cloitre M, Miranda R, Stovall-McClough KC, Han H. Beyond PTSD: Emotion regulation and interpersonal problems as predictors of functional impairment in survivors of childhood abuse. *Behavior Therapy.* 2005;36(2):119-124.
32. Dobie DJ, Kivlahan DR, Maynard C, Bush KR, Davis TM, Bradley KA. Posttraumatic stress disorder in female veterans: Association with self-reported health problems and functional impairment. *Archives of Internal Medicine.* 2004;164(4):394-400.
33. Shipherd JC, Keyes M, Jovanovic T, et al. Veterans seeking treatment for posttraumatic stress disorder: What about comorbid chronic pain? *Journal of Rehabilitation Research and Development.* 2007;44(2):153-165.
34. Haskell SG, Papas RK, Heapy A, Reid MC, Kerns RD. The association of sexual trauma with persistent pain in a sample of women veterans receiving primary care. *Pain Medicine.* 2008;9(6):710-717.
35. Caldwell BA, Redeker N. Sleep and trauma: an overview. *Issues in Mental Health Nursing.* 2005;26(7):721-738.
36. Kelly UA. Intimate partner violence, physical health, posttraumatic stress disorder, depression, and quality of life in Latinas. *Western Journal of Emergency Medicine.* 2010;11(3):247.
37. Wuest J, Ford-Gilboe M, Merritt-Gray M, et al. Abuse-Related Injury and Symptoms of Posttraumatic Stress Disorder as Mechanisms of Chronic Pain in Survivors of Intimate

Partner Violence. *Pain Medicine.* May-Jun 2009;10(4):739-747.
38. Crofford LJ. Violence, Stress, and Somatic Syndromes. *Trauma, Violence, & Abuse.* 2007;8(3):299- 313.
39. Kimerling R, Clum GA, Wolfe J. Relationships among trauma exposure, chronic posttraumatic stress disorder symptoms, and self-reported health in women: replication and extension. *Journal of traumatic stress.* 2000;13(1):115-128.
40. Corwin EJ, Williams L. *Handbook of pathophysiology.* 3rd ed. Philadelphia, PA: Lippincott Williams Publisher; 2007.
41. Pace TWW, Heim CM. A short review on the psychoneuroimmunology of posttraumatic stress disorder: from risk factors to medical comorbidities. *Brain, Behavior, and Immunity.* 2011;25(1):6-13.
42. Guo M, Liu T, Guo J-C, Jiang X-L, Chen F, Gao Y-S. Study on serum cytokine levels in posttraumatic stress disorder patients. *Asian Pacific journal of tropical medicine.* 2012;5(4):323-325.
43. Ren K, Dubner R. Interactions between the immune and nervous systems in pain. *Nature Medicine.* 2010;16(11):1267-1276.
44. Arora S, Bhattacharjee J. Modulation of immune responses in stress by Yoga. *International Journal Of Yoga.* 2008;1(2):45-55.
45. Steptoe A, Hamer M, Chida Y. The effects of acute psychological stress on circulating inflammatory factors in humans: a review and meta-analysis. *Brain, Behavior, And Immunity.* 2007;21(7):901-912.
46. Gill JM, Saligan L, Woods S, Page G. PTSD is associated with an excess of inflammatory immune activities. *Perspectives in Psychiatric Care.* 2009;45(4):262-277.
47. Sommer C, Kress M. Recent findings on how proinflammatory cytokines cause pain: peripheral mechanisms in inflammatory and neuropathic hyperalgesia. *Neuroscience Letters.* 2004;361(1-3):184- 187.
48. Hayley S, Poulter MO, Merali Z, Anisman H. The pathogenesis of clinical depression: stressor- and cytokine-induced alterations of neuroplasticity. *Neuroscience.* 2005;135(3):659-678.
49. Krueger JM. The role of cytokines in sleep regulation. *Current Pharmaceutical Design.* 2008;14(32):3408-3416.
50. Irwin M. Effects of sleep and sleep loss on immunity and cytokines. *Brain, Behavior, And Immunity.* 2002;16(5):503-512.
51. Spiegelhalder K, Fuchs L, Ladwig J, et al. Heart rate and heart rate variability in subjectively reported insomnia. *Journal of Sleep Research.* 2011;20(1 Pt 2):137-145.
52. Lavigne G, Smith MT, Denis R, Zucconi M. Pain and sleep. In: Kryger MH, Roth T, Dement WC, eds. *Principals and Practice of Sleep Medicine*. 5th ed. St. Louis, MO: Elsevier Saunders; 2011:1442-1451.
53. Balasubramaniam M, Telles S, Doraiswamy PM. Yoga on our minds: a systematic review of yoga for neuropsychiatric disorders. *Frontiers in psychiatry.* 2012;3.
54. Rani K, Tiwari S, Singh U, Singh I, Srivastava N. Yoga Nidra as a complementary treatment of anxiety and depressive symptoms in patients with menstrual disorder. *International Journal of Yoga.* 2012;5(1):52.
55. Li AW, Goldsmith CW. The Effects of Yoga on Anxiety and Stress. *Alternative Medicine Review.* 2012;17(1):21-35.
56. Khalsa SBS. Treatment of Chronic Insomnia with Yoga: A Preliminary Study with Sleep-Wake Diaries. *Applied Psychophysiology & Biofeedback.* 2004;29(4):269-278.
57. Afonso RF, Hachul H, Kozasa EH, et al. Yoga decreases insomnia in postmenopausal women: a randomized clinical trial. *Menopause.* 2012;19(2):186.
58. Büssing A, Ostermann T, Lüdtke R, Michalsen A. Effects of yoga interventions on pain and pain- associated disability: a meta-analysis. *The Journal of Pain.* 2012;13(1):1-9.

59. Cramer H, Lauche R, Haller H, Dobos G. A systematic review and meta-analysis of yoga for low back pain. *The Clinical journal of pain.* 2013;29(5):450-460.
60. Groessl EJ, Weingart KR, Johnson N, Baxi S. The benefits of yoga for women veterans with chronic low back pain. *The journal of alternative and complementary medicine.* 2012.
61. Smeeding SJW, Bradshaw DH, Kumpfer K, Trevithick S, Stoddard GJ. Outcome evaluation of the Veterans Affairs Salt Lake City Integrative Health Clinic for chronic pain and stress-related depression, anxiety, and post-traumatic stress disorder. *Journal of Alternative and Complementary Medicine.* 2010;16(8):823-835.
62. Stoller CC, Greuel JH, Cimini LS, Fowler MS, Koomar JA. Effects of Sensory-Enhanced Yoga on Symptoms of Combat Stress in Deployed Military Personnel. *The American Journal of Occupational Therapy.* 2012;66(1):59-68.
63. Emerson D, Hopper E, Levine PA, Cope S, Van Der Kolk B. *Overcoming Trauma Through Yoga: Reclaiming Your Body.* North Atlantic Books; 2011.
64. Barr CT. Depression, heart rate related variables and cardiovascular disease. *International Journal of Psychophysiology.* 2010;78(1):80-88.
65. Thayer JF, Yamamoto SS, Brosschot JF. The relationship of autonomic imbalance, heart rate variability and cardiovascular disease risk factors. *International Journal of Cardiology.* 2010;141(2):122-131.
66. Jovanovic T, Norrholm SD, Sakoman AJ, Esterajher S, Kozaric-Kovacic D. Altered resting psychophysiology and startle response in Croatian combat veterans with PTSD. *International Journal of Psychophysiology.* 2009;71(3):264-268.
67. Kleim B, Wilhelm FH, Glucksman E, Ehlers A. Sex differences in heart rate responses to script-driven imagery soon after trauma and risk of posttraumatic stress disorder. *Psychosomatic Medicine.* 2010;72(9):917-924.
68. Lee EAD, Theus SA. Lower heart rate variability associated with military sexual trauma, rape, and posttraumatic stress disorder. *Biological Research For Nursing.* 2012;14(4):412-418.
69. Norrholm SD, Jovanovic T, Olin IW, Sands LA, Bradley B, Ressler KJ. Fear extinction in traumatized civilians with posttraumatic stress disorder: relation to symptom severity. *Biological Psychiatry.* 2011;69(6):556-563.
70. Jovanovic T, Kazama A, Bachevalier J, Davis M. Impaired safety signal learning may be a biomarker of PTSD. *Neuropharmacology.* 2011.
71. Loggia ML, Juneau M, Bushnell MC. Autonomic responses to heat pain: Heart rate, skin conductance, and their relation to verbal ratings and stimulus intensity. *Pain.* 2011;152(3):592-598.
72. Raghuraj P, Telles S. Immediate Effect of Specific Nostril Manipulating Yoga Breathing Practices on Autonomic and Respiratory Variables. *Applied Psychophysiology & Biofeedback.* 2008;33(2):65-75.
73. Khattab K, Khattab AA, Ortak J, Richardt G, Bonnemeier H. Iyengar yoga increases cardiac parasympathetic nervous modulation among healthy yoga practitioners. *Evidence-Based Complementary And Alternative Medicine: Ecam.* 2007;4(4):511-517.
74. Bernardi L, Sleight P, Bandinelli G, et al. Effect of rosary prayer and yoga mantras on autonomic cardiovascular rhythms: Comparative study. *BMJ: British Medical Journal.* 2001;323(7327):1446-1449.
75. Satyapriya M, Nagendra HR, Nagarathna R, Padmalatha V. Effect of integrated yoga on stress and heart rate variability in pregnant women. *International Journal Of Gynaecology And Obstetrics: The Official Organ Of The International Federation Of Gynaecology And Obstetrics.* 2009;104(3):218-222.
76. Kiecolt-Glaser JK, Christian L, Preston H, et al. Stress, inflammation, and yoga practice. *Psychosomatic Medicine.* 2010;72(2):113-121.

77. Stone, Spinazzola, Sharma, et al. Yoga as a Complementary Treatment for Chronic PTSD. International Society for Traumatic Stress Studies 27th Annual Meeting; 2011; Baltimore, MD.
78. Vogt, D., et al., Deployment risk and resilience inventory-2 (DRRI-2): an updated tool for assessing psychosocial risk and resilience factors among service members and veterans. J Trauma Stress, 2013. 26(6): p. 710-7.
79. Gray M, Litz B, Hsu J, Lombardo T. Psychometric properties of the Life Events Checklist. *Assessment.* 2004;11:330-341.
80. Weathers FW, Keane TM, Davidson JR. Clinician-Administered PTSD Scale: A review of the first ten years of research. *Depression and Anxiety.* 2001;13(3):132-156.
81. Weathers FW, Litz BT, Huska JA, Keane TM. *PTSD Checklist—Civilian version.* Boston: National Center for PTSD, Behavioral Science Division;1994.
82. Sheehan DV, Lecrubier Y, Harnett-Sheehan K, Amorim P, Janavs J, Weiller E, Hergueta T, Baker R, Dunbar G: The Mini International Neuropsychiatric Interview (M.I.N.I): The Development and Validation of a Structured Diagnostic Psychiatric Interview. J. Clin Psychiatry, 1998; 59 (suppl 20): 22-33.
83. Beck AT, Steer RA, Ball R, Ranieri WF. Comparison of Beck Depression Inventories-IA and -II in Psychiatric Outpatients. *Journal of Personality Assessment.* 1996;67(3):588.
84. Cleeland CS, Ryan KM. Pain assessment: global use of the Brief Pain Inventory. Ann Acad Med Singapore 23(2): 129-138, 3/1994.
85. Carpenter JS, Andrykowski MA. Psychometric evaluation of the Pittsburgh sleep quality index. *Journal of Psychosomatic Research.* 1998;45(1):5-13.
86. Netzer NC, Stoohs RA, Netzer CM, Clark K, Strohl KP. Using the Berlin Questionnaire to identify patients at risk for sleep apnea syndrome. *Annals of Internal Medicine.* 1999;131:485-491. doi:10.7326/0003-4819-131-7-199910050-00002
87. Johns MW. A new method for measuring daytime sleepiness: the Epworth Sleepiness Scale. *Sleep* 1991; 50-55
88. American Psychiatric Association. *Diagnostic and Statistical Manual of Mental Disorders, Fifth Edition.* 4th, Text Revision ed. Arlington, VA: American Psychiatric Association; 2013.
89. Usman Iqbal, S., W. Rogers, A. Selim, S.X. Qian, A. Lee, X. Xinhua, J. Rothendler, D. Miller, L. Kazis. “The Veterans Rand 12 Item Health Survey (Vr-12): What It Is and How It Is Used”. Technical report.
90. J. Parak and I. Korhonen, "Accuracy of Firstbeat Bodyguard 2 beat-to-beat heart rate monitor", *(whitepaper)*, 2013.
91. Harris P, Taylor R, Thielke R, Payne J, Gonzalez N, Conde J. Research electronic data capture (REDCap) - A metadata-driven methodology and workflow process for providing translational research informatics support. *J Biomedical Informatics.* 2009;42(2):377-381.
92. Hedeker DR, Gibbons RD. *Longitudinal data analysis.* Hoboken, NJ: John Wiley and Sons; 2006.
93. *PASS 13* [computer program]. Kaysville, UT: NCSS, LLC.; 2014.

Singer JD, Willett JB. *Applied longitudinal data analysis: Modeling change and event occurrence.* First ed. New York: Oxford University Press; 2003.

1678
