# Supplementary material for: Yoga vs Cognitive Processing Therapy for Military Sexual Trauma–Related Posttraumatic Stress Disorder: A Randomized Clinical Trial
Source: JAMA Netw Open. 2023 Dec 8;6(12):e2344862. doi: 10.1001/jamanetworkopen.2023.44862 (PMC10709771; doi:10.1001/jamanetworkopen.2023.44862)
Supplement: Supplement 2. — eFigure 1. CONSORT Chart for Primary Site (Site 1) eFigure 2. CONSORT Chart for Second Site (Site 2) eFigure 3. Number of Sessions Attended by Group eMethods 1. Equivalence Tests: Data Analysis Details eMethods 2. COVID Sensitivity Analysis eTable 1. Demographics, Clinical Characteristics, and Trauma Exposure by Study Site eTable 2. CAPS-5 Intent-to-Treat and Per-Protocol Group Differences Detailed eTable 3. PCL-5 Intent-to-Treat and Per-Protocol Group Differences Detailed eFigure 4. PTSD Severity Scores Over Time eFigure 5. CAPS-5 and PCL-5 Severity Scores eTable 4. CAPS-5 and PCL-5 Intent-to-Treat (ITT) and Per-Protocol (PP) Within-Group Differences From Baseline eTable 5. Tests of Equivalence: CAPS-5 Severity and PCL-5 Change Scores From Baseline eTable 6. CAPS-5 Severity and PCL-5 Change Scores From Baseline by Time and Group and Group Equivalence Tests (ITT and PP) eTable 7. Clinical PTSD Diagnostic Changes [file jamanetwopen-e2344862-s002.pdf]

Supplemental Online Content

Zaccari B, Higgins M, Haywood T, et al. Effectiveness of yoga vs cognitive processing therapy for military sexual trauma-related posttraumatic stress disorder: a randomized clinical trial. *JAMA Netw Open*. 2023;6(12):e2344862. doi:10.1001/jamanetworkopen.2023.44862

eFigure 1. CONSORT Chart for Primary Site (Site 1)

eFigure 2. CONSORT Chart for Second Site (Site 2)

eFigure 3. Number of Sessions Attended by Group

eMethods 1. Equivalence Tests: Data Analysis Details

eMethods 2. COVID Sensitivity Analysis

eTable 1. Demographics, Clinical Characteristics, and Trauma Exposure by Study Site

eTable 2. CAPS-5 Intent-to-Treat and Per-Protocol Group Differences Detailed

eTable 3. PCL-5 Intent-to-Treat and Per-Protocol Group Differences Detailed

eFigure 4. PTSD Severity Scores Over Time

eFigure 5. CAPS-5 and PCL-5 Severity Scores

eTable 4. CAPS-5 and PCL-5 Intent-to-Treat (ITT) and Per-Protocol (PP) Within-Group Differences from Baseline

eTable 5. Tests of Equivalence: CAPS-5 Severity and PCL-5 Change Scores from Baseline

eTable 6. CAPS-5 Severity and PCL-5 Change Scores from Baseline by Time and Group and Group Equivalence Tests (ITT and PP)

eTable 7. Clinical PTSD Diagnostic Changes

This supplemental material has been provided by the authors to give readers additional information about their work.

© 2023 Zaccari B et al. *JAMA Network Open.*

eFigure 1. Consort Chart for Primary Site (Site 1)

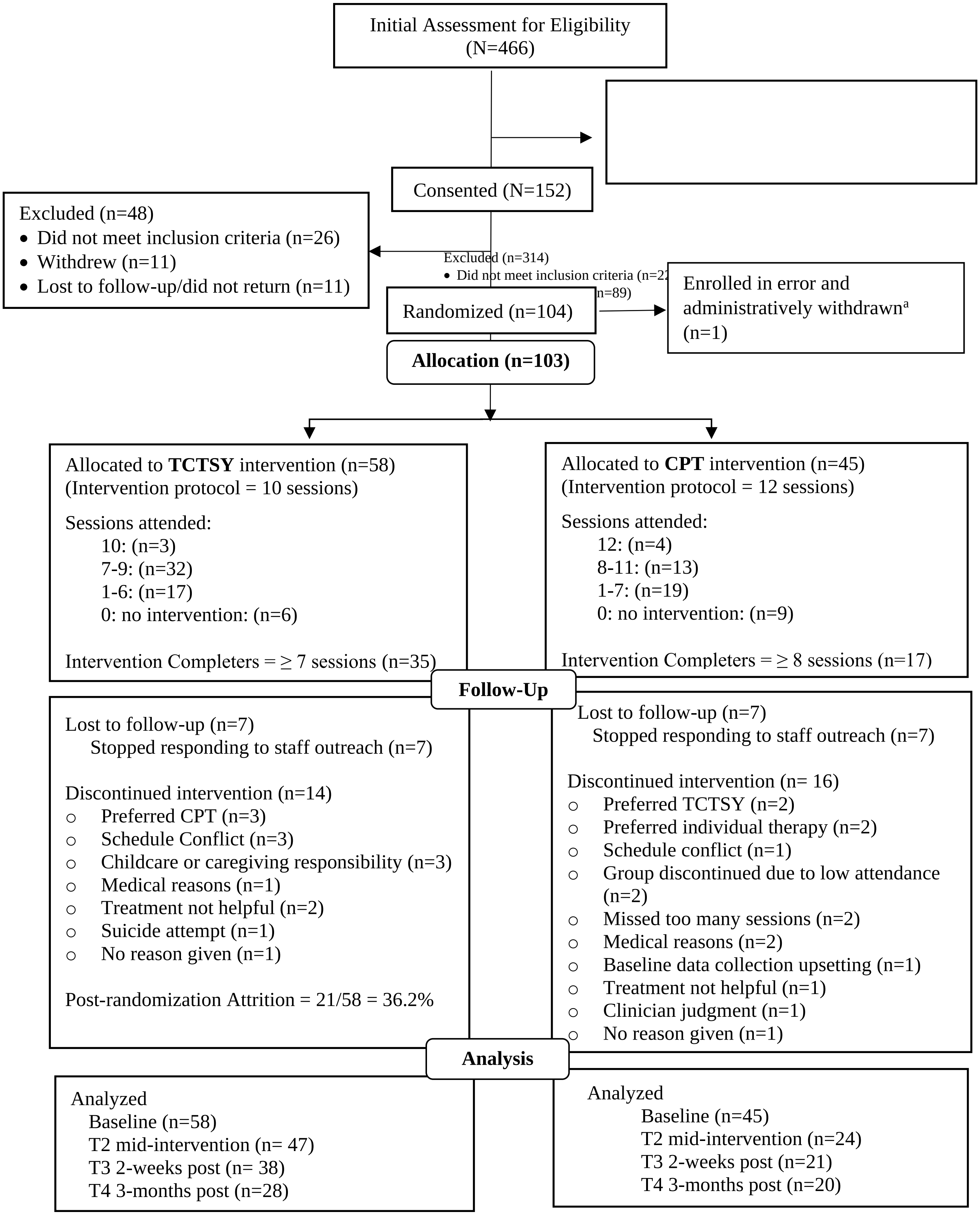

[a] Participant was consented in error; did not meet eligibility criteria for PTSD related to MST.

© 2023 Zaccari B et al. *JAMA Network Open.*

eFigure 2. Consort Chart for Second Site (Site 2)

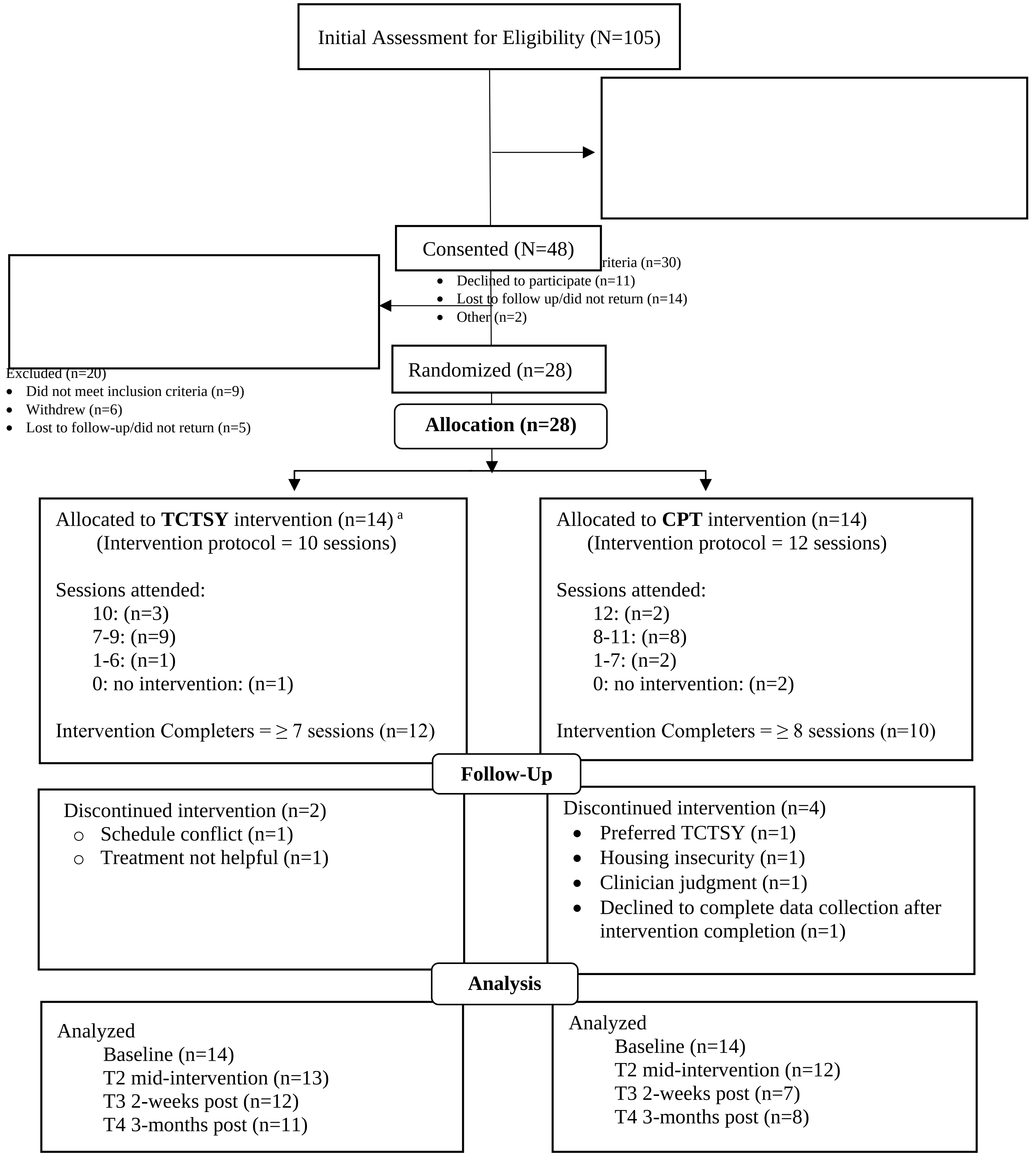

[a]One participant who completed TCTSY was discovered to have a history of a moderate TBI, and therefore was enrolled in error.

© 2023 Zaccari B et al. *JAMA Network Open.*

eFigure 3. Number of Sessions Attended by Group: 3A Both Sites; 3B Site 1; 3C Site 2

eFigure 3A: Number of Sessions Attended: Both Sites

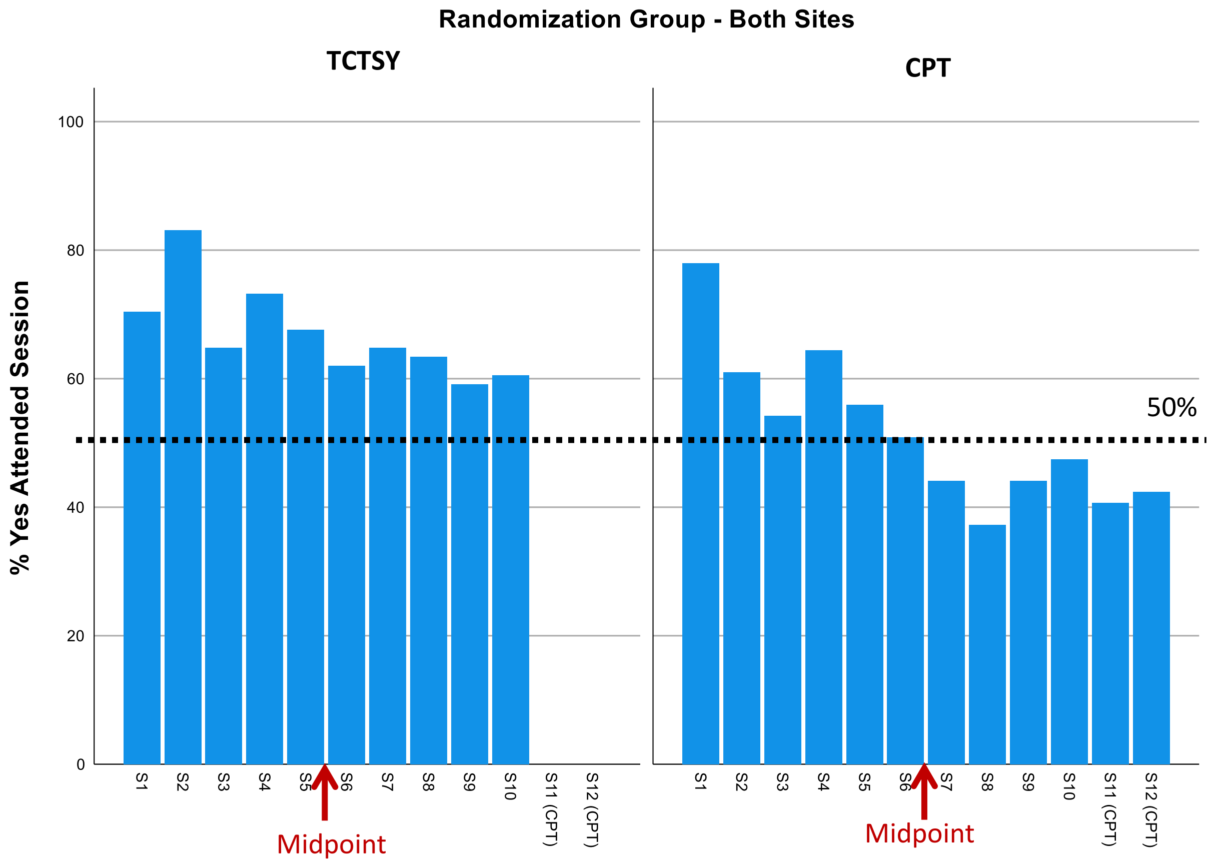

Legend: S=session

© 2023 Zaccari B et al. *JAMA Network Open.*

eFigure 3B: Number of Sessions Attended: Site 1

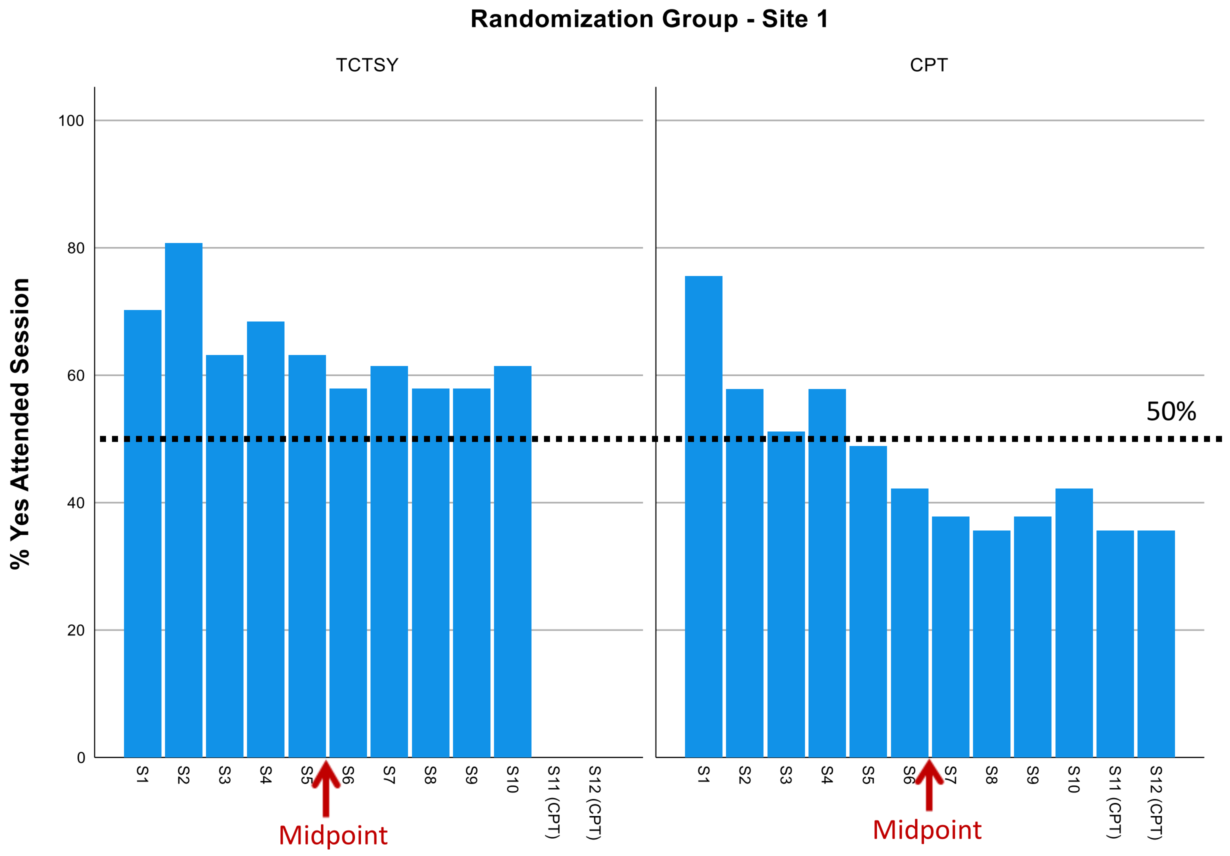

Legend: S=session

© 2023 Zaccari B et al. *JAMA Network Open.*

eFigure 3C: Number of Sessions Attended: Site 2

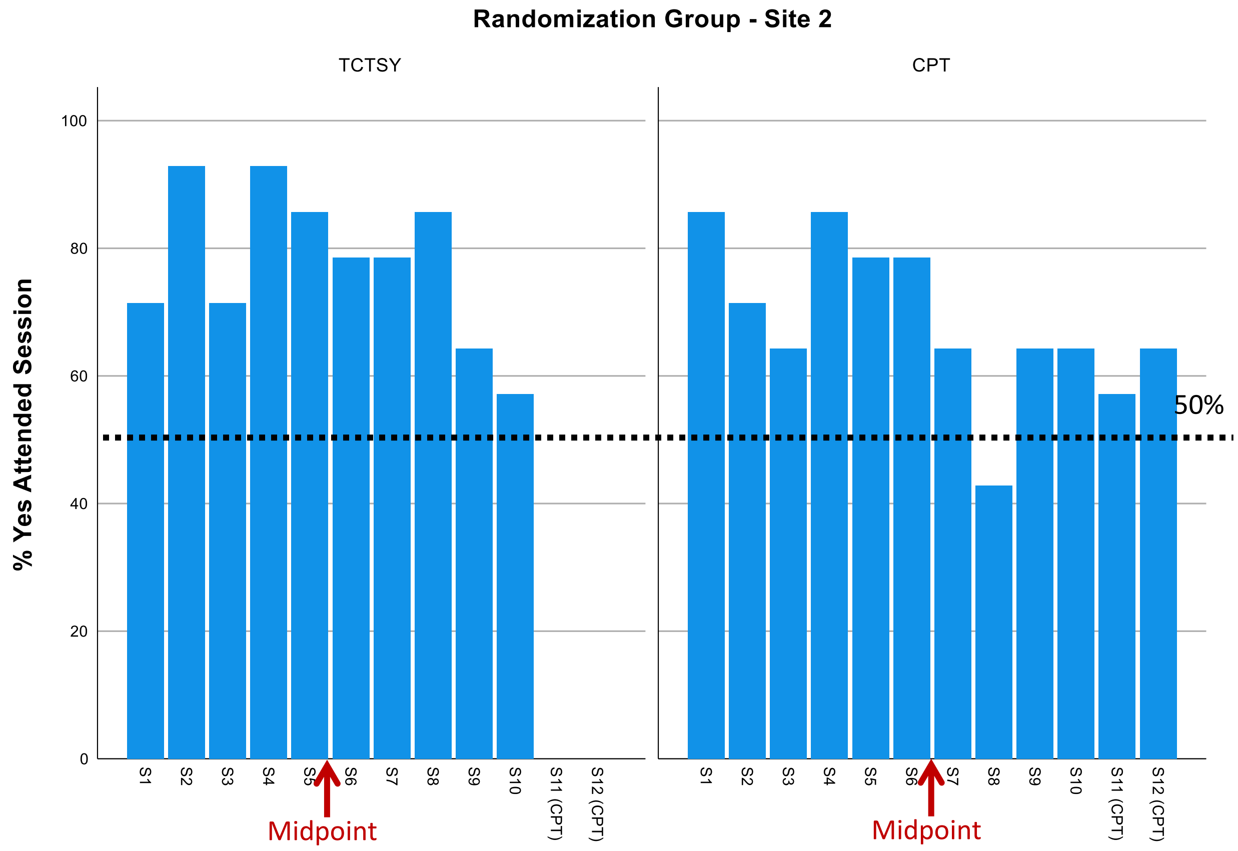

Legend: S=session

© 2023 Zaccari B et al. *JAMA Network Open.*

eMethods 1. Equivalence Tests: Data Analysis Details

Statistical Analysis Post hoc: Equivalence Testing

Detailed Methods:
While the original RCT was designed for inequality hypothesis testing, given the lack of differences seen between the 2 treatments (both performed well), we additionally conducted equivalence tests of means between the two treatment groups using two one-sided unequal-variance t-tests (TOST). These equivalence tests were performed using the TOSTER v.0.4.0 package for R [REFS Lakens (2017); Lakens, Scheel and Isager (2018)]. The changes from baseline to each follow-up time point were computed for each participant. These change scores were then used to perform two one-sided t-tests (TOST) between the two treatment groups to test for equivalence and reject the presence of the smallest effect size of interest (SESOI). This approach essentially checks to see if the 90% confidence interval bounds for the mean difference between the two groups are within the limits defined by the SESOI. Similar to Sloan, et.al. (2022), a margin of 10 was used as the SESOI. Sloan was only interested in one-side of the margin and performed non-inferiority tests since they assumed one treatment might be inferior to the other. However, we did not have an initial assumption that one treatment would be better than the other, so two one-sided tests or TOSTs were performed to test for equivalence within +/- 10 points. This SESOI was used for both the CAPS-5 and PCL-5 change score differences between the two treatment groups. It has also been suggested that a difference as small as 5 points for the CAPS-5 may be useful for comparing treatments for PTSD so the mean and standard deviation of all changes scores are also reported by group and time for descriptive comparisons. For clinical diagnostic comparisons, similar to Schnurr, et.al. (2022), the percentage of participants at each time point who still have PTSD are reported as well as the percentage who achieved a clinical response (reduction of CAPS-5 severity scores by 10 or more points from baseline), loss of diagnosis (CAPS-5 change <= -10, no PTSD diagnosis and CAPS-5 severity score <25), as well as those who achieved remission (loss of diagnosis plus CAPS-5 score < 12). All statistical analyses completed using SPSS version 27.0.0.0 [IBM Corp (2020)] and R version 4.1.2 [R Core Team (2021)].

© 2023 Zaccari B et al. *JAMA Network Open.*

eMethods 2. COVID Sensitivity Analysis

Statistical Analysis Post hoc: COVID Sensitivity Analysis:

Methods:
Site 1 had 9 cohorts of participants who enrolled from Sept 2015 to Jan 2020 and Site 2 had participants who enrolled from June 2019 through May 2020. Due to COVID, the last cohort at Site 1 was conducted using a hybrid/virtual format and Site 2 was also conducted entirely virtually. Given these protocol deviations, sensitivity analyses were conducted running all of the analyses described in the methods section of the manuscript with and without Site 2 and with and without the 9th (last) cohort at Site 1.

Results:
While the sample sizes were smaller after removal of Site 2 and without cohort 9, the estimated effect sizes and statistical significance p-values were similar indicating that the effects of the protocol deviations (in person versus virtual) and COVID impact were minimal leading to the same conclusions. The results are presented for the full dataset in the main manuscript were robust (insensitive) to these COVID-related issues.

© 2023 Zaccari B et al. *JAMA Network Open.*

eTable 1. Demographics, Clinical Characteristics, and Trauma Exposure by Study Site

	Atlanta (N=103)	Portland (N=28)	Total (N=131)	p value
**Age**				0.700[1]
Mean (SD)	48.4 (11.1)	47.5 (11.7)	48.23 (11.23)	
Range	22 - 71	32 – 68	22 - 71	
**Race Black/African American v Other**				< 0.001[2]
African American	93 (90%)	2 (7%)	95 (72.5%)	
Non-African American	10 (10%)	26 (93%)	36 (27.5%)	
**Race White v Other**				< 0.001[2]
White	1 (1%)	24 (86%)	25 (19.1%)	
non-White	102 (99%)	4 (14%)	106 (80.9%)	
**Relationship Status**				0.105[2]
non-partnered	72 (70%)	15 (54%)	87 (66.4%)	
married/partnered	31 (30%)	13 (46%)	44 (33.6%)	
**Education Level**				0.060[2]
less than college	46 (45%)	7 (25%)	53 (40.5%)	
college degree or more	57 (55%)	21 (75%)	78 (59.5%)	
**Monthly Income**				0.014[2]
less than $2K/month	44 (43%)	5 (18%)	49 (37.7%)	
$2K/month or more	58 (57%)	23 (82%)	81 (62.3%)	
**Employment**				0.534[2]
less than full time	71 (69%)	21 (75%)	92 (70.2%)	
full time	32 (31%)	7 (25%)	39 (29.8%)	
**Depression**				
**Beck Depression Inventory (BDI) Total**				0.402[1]
N-Miss	5	0	5	
Mean (SD)	28.9 (10.3)	27.0 (12.2)	28.47 (10.72)	
Range	5 - 58	8 - 55	5 - 58	
**BDI Total Categories**				0.159[2]
N-Miss	5	0	5	
0-13 Minimal	5 (5%)	5 (18%)	10 (7.9%)	
14-19 Mild	12 (12%)	4 (14%)	16 (12.7%)	
20-28 Moderate	32 (33%)	7 (25%)	39 (31.0%)	
29-63 Severe	49 (50%)	12 (43%)	61 (48.4%)	
**Suicidality (Past Month) MINI-5**				0.036[2]
No	70 (68%)	13 (46%)	83 (63.4%)	
Yes	33 (32%)	15 (54%)	48 (36.6%)	
**Suicidality (Lifetime Attempt) MINI-5**				0.642[2]
No	78 (76%)	20 (71%)	98 (74.8%)	
Yes	25 (24%)	8 (29%)	33 (25.2%)	
**Suicidality (MINI-5)**				0.750[3]
N-Miss	70	13	83	
1. 1-8 Low	15 (46%)	9 (60%)	24 (50.0%)	
2. 9-16 Moderate	5 (15%)	1 (7%)	6 (12.5%)	
3. =>17 High	13 (39%)	5 (33%)	18 (37.5%)	
**Lifetime Trauma Experiences (LEC)**				
**1. Fire or explosion**				
Happened to Me	27 (26%)	7 (25%)	34 (26.0%)	0.897[2]
Witnessed it	23 (22%)	9 (32%)	32 (24.4%)	0.284[2]
**2. Transportation accident (for example, car accident, boat accident, train wreck, plane crash)**				
Happened to me	82 (80%)	21 (75%)	103 (78.6%)	0.598[2]

© 2023 Zaccari B et al. *JAMA Network Open.*

	Atlanta (N=103)	Portland (N=28)	Total (N=131)	p value
Witnessed it	28 (27%)	10 (36%)	38 (29.0%)	0.378[2]
**3. Serious accident at work, home, or during recreational activity**				
Happened to me	38 (37%)	13 (46%)	51 (38.9%)	0.359[2]
Witnessed it	22 (21%)	13 (46%)	35 (26.7%)	0.008[2]
**4. Exposure to toxic substance (for example, dangerous chemicals, radiation)**				
Happened to me	26 (25%)	9 (32%)	35 (26.7%)	0.464[2]
Witnessed it	7 (7%)	3 (11%)	10 (7.6%)	0.445[3]
**5. Physical assault (for example, being attacked, hit, slapped, kicked, beaten up)**				
Happened to me	90 (87%)	24 (86%)	114 (87.0%)	0.759[3]
Witnessed it	24 (23%)	10 (36%)	34 (26.0%)	0.184[2]
**6. Assault with a weapon (for example, being shot, stabbed, threatened with a knife, gun, bomb)**				
Happened to me	37 (36%)	14 (50%)	51 (38.9%)	0.176[2]
Witnessed it	21 (20%)	4 (14%)	25 (19.1%)	0.466[2]
**7. Sexual assault (rape, attempted rape, made to perform any type of sexual act through force or threat of harm)**				
Happened to me	102 (99%)	27 (96%)	129 (98.5%)	0.383[3]
Witnessed it	5 (5%)	3 (11%)	8 (6.1%)	0.367[3]
**8. Other unwanted or uncomfortable sexual experience**				
Happened to me	93 (90%)	26 (93%)	119 (90.8%)	1.000[3]
Witnessed it	8 (8%)	6 (21%)	14 (10.7%)	0.076[3]
**9. Combat or exposure to a warzone (in the military or as a civilian)**				
Happened to me	34 (33%)	9 (32%)	43 (32.8%)	0.931[2]
Witnessed it	7 (7%)	4 (14%)	11 (8.4%)	0.247[3]
**10. Captivity (for example, being kidnapped, abducted, held hostage, prisoner of war)**				
Happened to me	15 (15%)	7 (25%)	22 (16.8%)	0.252[3]
Witnessed it	1 (1%)	2 (7%)	3 (2.3%)	0.115[3]
**12. Life-threatening illness or injury**				
Happened to me	22 (21%)	10 (36%)	32 (24.4%)	0.117[2]
Witnessed it	33 (32%)	12 (43%)	45 (34.4%)	0.285[2]
**12. Severe human suffering**				
Happened to me	9 (9%)	3 (11%)	12 (9.2%)	0.719[3]
Witnessed it	22 (21%)	11 (39%)	33 (25.2%)	0.053[2]
**Childhood Trauma Questionnaire (CTQ) Physical Abuse**				0.634[1]
Mean (SD)	10.3 (4.4)	9.9 (3.5)	10.19 (4.21)	
Range	5 - 21	5 - 18	5.00 - 21.00	
**CTQ Emotional Neglect**				0.255[1]
Mean (SD)	12.0 (5.2)	13.3 (5.3)	12.25 (5.26)	
Range	5 - 25	5 - 23	5.00 - 25.00	
**CTQ Emotional Abuse**				0.194[1]
Mean (SD)	11.5 (5.4)	13.0 (5.4)	11.83 (5.38)	
Range	5 - 24	5 – 23	5.00 - 24.00	
**CTQ Physical Neglect**				0.480[1]
Mean (SD)	8.4 (3.6)	8.9 (3.4)	8.469 (3.57)	
Range	5 – 2	5 - 17	5.00 - 20.00	

© 2023 Zaccari B et al. *JAMA Network Open.*

	Atlanta (N=103)	Portland (N=28)	Total (N=131)	p value
**CTQ Physical Abuse**				0.337[1]
Mean (SD)	12.5 (7.1)	11.0 (6.9)	12.13 (7.02)	
Range	5 - 25	5 – 25	5.00 - 25.00	
**Combat Exposure (DRRI Combat Total)**				0.875[1]
Mean (SD)	20.3 (5.6)	20.5 (6.0)	20.39 (5.65)	
Range	17 - 50	17 – 38	17.00 - 50.00	

© 2023 Zaccari B et al. *JAMA Network Open.*

eTable 2. CAPS-5 Intent-to-Treat and Per-Protocol Group Differences Detailed

eTable 2: PTSD Outcome CAPS-5:Intent to Treat (ITT) and Per Protocol (PP) Group Difference Effect Sizes (Full Detail)

**CAPS-5**	**(ITT)**						**Group Diff**	**Sidak Adj**	**Effect Size**
**Group**	**Time**	**N**	**Mean**	**SD**	**95% CI LB**	**95% CI UB**	**Estimate +/- SE**	**p-value**	**Cohen's d**
TCTSY	Baseline	71	36.73	8.79	34.65	38.81	1.14 ± 1.88	.543	0.148
TCTSY	Midpoint	59	26.32	10.09	23.69	28.95	-1.15 ± 2.19	.600	-0.243
TCTSY	2 weeks	50	23.24	11.68	19.92	26.56	-2.93 ± 2.39	.221	-0.354
TCTSY	3 months	39	24.03	11.55	20.28	27.77	2.80 ± 2.45	.254	0.151
CPT	Baseline	58	35.52	7.49	33.55	37.49			
CPT	Midpoint	34	28.97	12.20	24.71	33.23			
CPT	2 weeks	26	27.77	14.77	21.80	33.74			
CPT	3 months	27	22.15	13.56	16.78	27.51			
Group: $F_{(1, 141.6)}$ = 0.0004, p = .984; Time: $F_{(3, 258.6)}$ = 48.1331, p < .001; Group-by-Time: $F_{(3, 258.6)}$ = 1.8721, p = .135									
**CAPS-5**	**(PP)**						**Group Diff**	**Sidak Adj**	**Effect Size**
**Group**	**Time**	**N**	**Mean**	**SD**	**95% CI LB**	**95% CI UB**	**Estimate +/- SE**	**p-value**	**Cohen's d**
TCTSY	Baseline	46	35.13	8.36	32.65	37.61	-2.19 ± 2.67	.413	-0.249
TCTSY	Midpoint	45	26.56	10.04	23.54	29.57	-3.83 ± 2.75	.166	-0.367
TCTSY	2 weeks	45	23.51	11.60	20.03	26.99	-4.31 ± 2.87	.134	-0.357
TCTSY	3 months	36	24.08	11.49	20.20	27.97	1.74 ± 2.91	.551	0.170
CPT	Baseline	27	37.22	8.51	33.86	40.59			
CPT	Midpoint	24	30.58	12.54	25.29	35.88			
CPT	2 weeks	20	28.05	15.03	21.01	35.09			
CPT	3 months	21	22.00	13.46	15.87	28.13			
Group: $F_{(1, 73.6)}$ = 0.9984, p = .321; Time: $F_{(3, 189.8)}$ = 31.7747, p < .001; Group-by-Time: $F_{(3, 189.8)}$ = 1.6087, p = .189									

© 2023 Zaccari B et al. *JAMA Network Open.*

eTable 3. PCL-5 Intent-to-Treat and Per-Protocol Group Differences Detailed

eTable 3: PTSD Outcome PCL-5 Intent to Treat (ITT) and Per Protocol (PP) Group Difference Effect Sizes (Full Detail)

**PCL-5**	**(ITT)**						**Group Diff**	**Sidak Adj**	**Effect Size**
**Group**	**Time**	**N**	**Mean**	**SD**	**95% CI LB**	**95% CI UB**	**Estimate +/- SE**	**p-value**	**Cohen's d**
TCTSY	Baseline	71	49.62	12.19	46.73	52.51	0.92 ± 2.60	.722	0.072
TCTSY	Midpoint	59	42.49	14.25	38.78	46.20	-3.04 ± 3.00	.312	-0.225
TCTSY	2 weeks	50	38.68	15.72	34.21	43.15	1.73 ± 3.26	.596	-0.019
TCTSY	3 months	39	36.97	17.74	31.22	42.72	4.20 ± 3.37	.214	0.200
CPT	Baseline	59	48.69	13.62	45.14	52.24			
CPT	Midpoint	35	45.83	15.77	40.41	51.25			
CPT	2 weeks	27	39.00	17.65	32.02	45.98			
CPT	3 months	27	33.59	15.51	27.46	39.73			
Group: $F_{(1, 139.2)}$ = 0.1737, p = .678; Time: $F_{(3, 257.2)}$ = 23.3036, p < .001; Group-by-Time: $F_{(3, 257.2)}$ = 1.5488, p = .202									
**PCL-5**	**(PP)**						**Group Diff**	**Sidak Adj**	**Effect Size**
**Group**	**Time**	**N**	**Mean**	**SD**	**95% CI LB**	**95% CI UB**	**Estimate +/- SE**	**p-value**	**Cohen's d**
TCTSY	Baseline	47	47.02	12.07	43.48	50.57	-0.46 ± 3.62	.899	-0.034
TCTSY	Midpoint	45	41.69	15.00	37.18	46.20	-2.72 ± 3.69	.463	-0.155
TCTSY	2 weeks	45	39.04	15.71	34.32	43.77	2.25 ± 3.84	.559	0.095
TCTSY	3 months	36	36.22	17.36	30.35	42.10	4.56 ± 3.94	.248	0.283
CPT	Baseline	27	47.48	15.53	41.34	53.62			
CPT	Midpoint	25	44.04	15.36	37.70	50.38			
CPT	2 weeks	21	37.52	16.76	29.89	45.16			
CPT	3 months	21	31.76	12.47	26.09	37.44			
Group: $F_{(1, 74.1)}$ = 0.0967, p = .757; Time: $F_{(3, 192.5)}$ = 16.7132, p < .001; Group-by-Time: $F_{(3, 192.5)}$ = 1.2716, p = .285									

© 2023 Zaccari B et al. *JAMA Network Open.*

eFigure 4. PTSD Severity Scores Over Time: [A] CAPS-5 Severity Scores (ITT Sample); [B] PCL-5 Severity Scores (ITT Sample)

[caption: plot on left shows the means +/- 95% confidence interval (CI) limits for the Original Scores; plot on the right show the average within subject changes from baseline for the Scores +/- 95% CI limits]

[A] CAPS-5 Severity Scores (ITT)

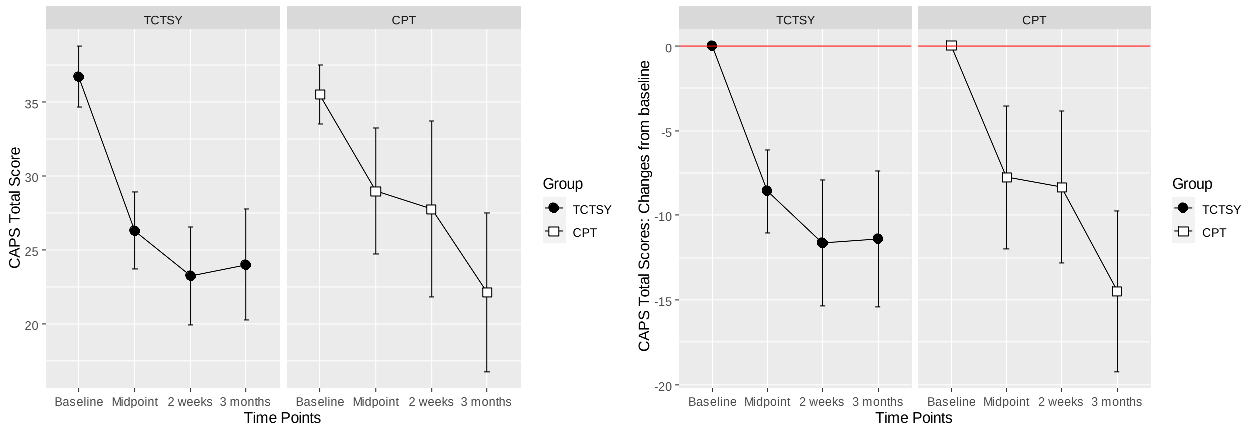

[B] PCL-5 Severity Scores (ITT)

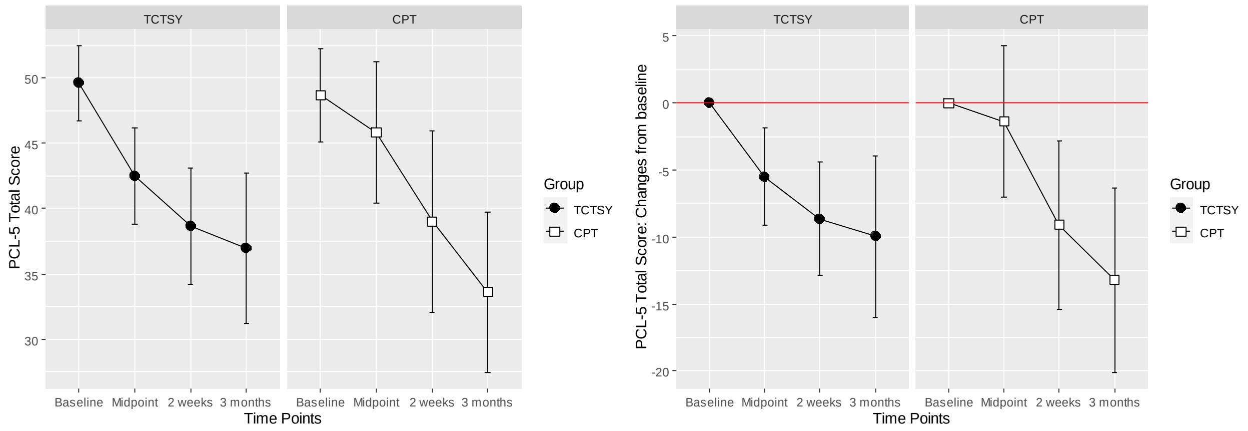

© 2023 Zaccari B et al. *JAMA Network Open.*

eFigure 5. CAPS-5 and PCL-5 Severity Scores: [A] CAPS-5 Severity Scores (PP sample); [B] PCL-5 Severity Scores (PP Sample)

[caption: plot on left shows the means +/- 95% confidence interval (CI) limits for the Original Scores; plot on the right show the average within subject changes from baseline for the Scores +/- 95% CI limits]

[A] CAPS-5 Severity Scores (PP)

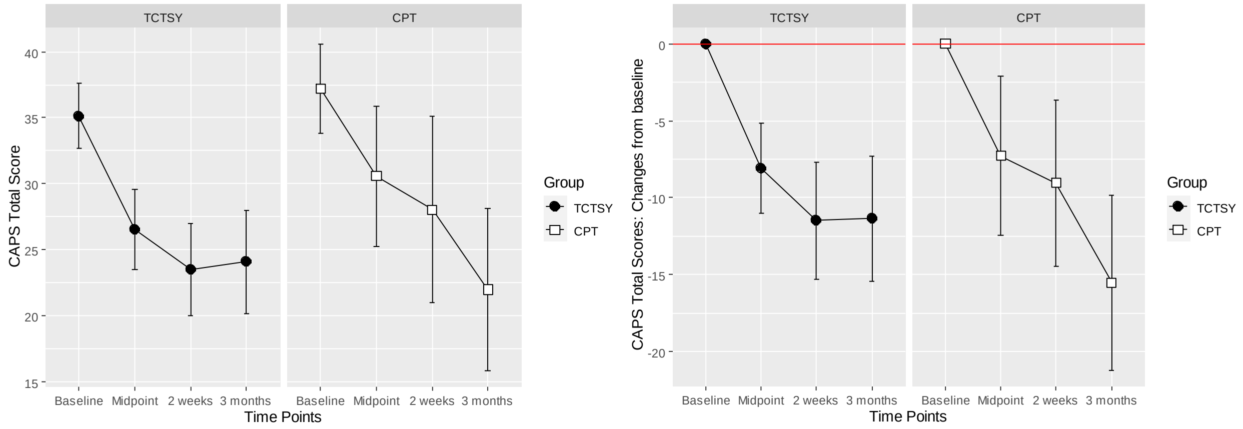

[B] PCL-5 Severity Scores (PP)

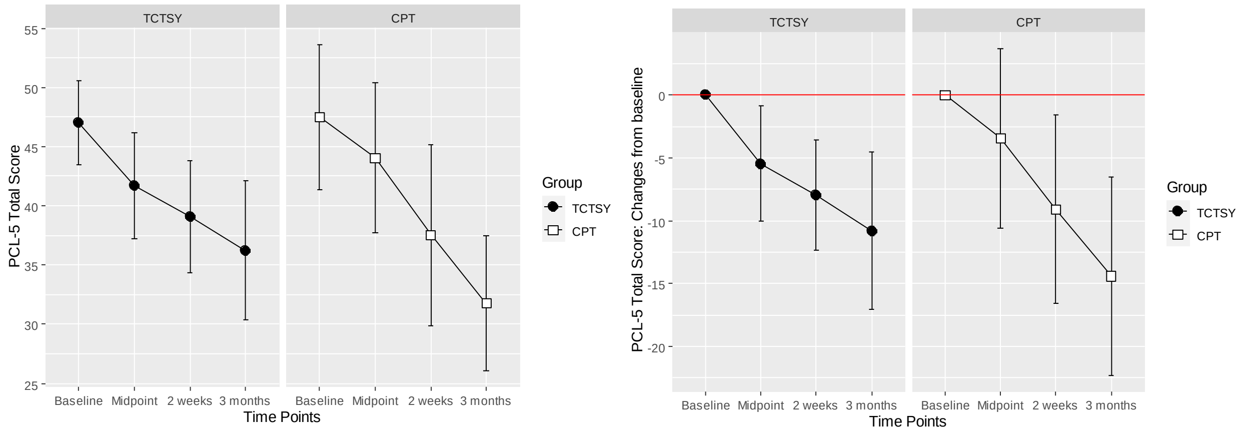

© 2023 Zaccari B et al. *JAMA Network Open.*

eTable 4. CAPS-5 and PCL-5 Intent-to-Treat (ITT) and Per-Protocol (PP) Within-Group Differences from Baseline

CAPS-5 (ITT)			Within Group Changes From Baseline		Within Group Changes From Baseline
**Group**	**Time - Baseline**	**N**	**Mean**	**SD**	**Effect Size**
TCTSY	Midpoint – BL	58	-8.59	9.32	-0.92
TCTSY	2 weeks – BL	49	-11.65	12.94	-0.90
TCTSY	3 months – BL	39	-11.41	12.32	-0.93
CPT	Midpoint – BL	34	-7.76	12.05	-0.64
CPT	2 weeks – BL	26	-8.35	11.06	-0.75
CPT	3 months – BL	27	-14.48	12.01	-1.21
**CAPS-5 (PP)**					
**Group**	**Time - Baseline**	**N**	**Mean**	**SD**	**Effect Size**
TCTSY	Midpoint – BL	44	-8.11	9.66	-0.84
TCTSY	2 weeks – BL	44	-11.50	12.55	-0.92
TCTSY	3 months – BL	36	-11.36	11.98	-0.95
CPT	Midpoint – BL	24	-7.29	12.27	-0.59
CPT	2 weeks – BL	20	-9.05	11.52	-0.79
CPT	3 months – BL	21	-15.52	12.51	-1.24
**PCL-5 (ITT)**					
**Group**	**Time - Baseline**	**N**	**Mean**	**SD**	**Effect Size**
TCTSY	Midpoint – BL	59	-5.49	13.83	-0.40
TCTSY	2 weeks – BL	50	-8.64	14.93	-0.58
TCTSY	3 months – BL	39	-9.95	18.60	-0.53
CPT	Midpoint – BL	35	-1.37	16.50	-0.08
CPT	2 weeks – BL	27	-9.11	15.85	-0.57
CPT	3 months – BL	27	-13.22	17.39	-0.76
**PCL-5 (PP)**					
**Group**	**Time - Baseline**	**N**	**Mean**	**SD**	**Effect Size**
TCTSY	Midpoint – BL	45	-5.47	15.20	-0.36
TCTSY	2 weeks – BL	45	-7.98	14.66	-0.54
TCTSY	3 months – BL	36	-10.81	18.45	-0.59
CPT	Midpoint – BL	25	-3.44	17.29	-0.20
CPT	2 weeks – BL	21	-9.10	16.47	-0.55
CPT	3 months – BL	21	-14.43	17.28	-0.84

© 2023 Zaccari B et al. *JAMA Network Open.*

eTable 5. Tests of Equivalence: CAPS-5 Severity and PCL-5 Change Scores from Baseline

	Midpoint - BL		2 Week Post - BL		3 Month Post - BL	
CAPS-5 (ITT)	n	Mean Diff (SD)	n	Mean Diff (SD)	n	Mean Diff (SD)
TCTSY	58	-8.59 (9.32)	49	-11.65 (12.94)	39	-11.41 (12.32)
CPT	34	-7.76 (12.05)	26	-8.35 (1.06)	27	-14.48 (12.01)
∆ Groups						
Mean ± SE		-0.82 ± 2.25		-3.31 ± 2.99		3.07 ± 3.05
p-value		.716		.273		.318
Equivalence Bounds [LB, UB]		[-4.56, 2.91]		[-8.29, 1.68]		[-2.03, 8.17]
CAPS-5 (PP)	n	Mean Diff (SD)	n	Mean Diff (SD)	n	Mean Diff (SD)
TCTSY	44	-8.11 (9.66)	44	-11.50 (12.55)	36	-11.36 (11.98)
CPT	24	-7.29 (12.27)	20	-9.05 (1.52)	21	-15.52 (12.51)
∆ Groups						
Mean ± SE		-0.82 ± 2.70		-2.45 ± 3.30		4.16 ± 3.34
p-value		.762		.461		.218
Equivalence Bounds [LB, UB]		[-5.33, 3.68]		[-7.97, 3.06]		[-1.43, 9.76]
PCL-5 (ITT)	n	Mean Diff (SD)	n	Mean Diff (SD)	n	Mean Diff (SD)
TCTSY	59	-5.49 (13.83)	50	-8.64 (14.93)	39	-9.95 (18.60)
CPT	35	-1.37 (16.50)	27	-9.11 (15.85)	27	-13.22 (17.39)
∆ Groups						
Mean ± SE		-4.12 ± 3.17		0.47 ± 3.64		3.27 ± 4.54
p-value		.197		.897		.473
Equivalence Bounds [LB, UB]		[-9.39, 1.15]		[-5.60, 6.54]		[-4.30, 10.84]
PCL-5 (PP)	n	Mean Diff (SD)	n	Mean Diff (SD)	n	Mean Diff (SD)
TCTSY	45	-5.47 (15.20)	45	-7.98 (14.66)	36	-10.81 (18.45)
CPT	25	-3.44 (17.29)	21	-9.10 (16.47)	21	-14.43 (17.28)
∆ Groups						
Mean ± SE		-2.06 ± 3.98		1.12 ± 4.03		3.62 ± 4.95
p-value		.613		.782		.468
Equivalence Bounds [LB, UB]		[-8.67, 4.62]		[-5.61, 7.84]		[-4.66, 11.91]

© 2023 Zaccari B et al. *JAMA Network Open.*

eTable 6. CAPS-5 Severity and PCL-5 Change Scores From Baseline by Time and Group and Group Equivalence Tests (ITT and PP)

CAPS-5	(ITT)		Change from BL		Δ from BL	Group Diff		Mean Group Diff	Equivalence Test		Equivalence Bounds
Group	Time - Baseline	N	Mean	SD	Cohen's d	Estimate +/- SE	p-value	Within +/- 5	TOST LB	TOST UB	Within +/- 10
TCTSY	Midpoint – BL	58	-8.59	9.32	-0.92	-0.82 ± 2.25	.716	YES	-4.56	2.91	YES
TCTSY	2 weeks – BL	49	-11.65	12.94	-0.90	-3.31 ± 2.99	.273	YES	-8.29	1.68	YES
TCTSY	3 months – BL	39	-11.41	12.32	-0.93	3.07 ± 3.05	.318	YES	-2.03	8.17	YES
CPT	Midpoint – BL	34	-7.76	12.05	-0.64						
CPT	2 weeks – BL	26	-8.35	11.06	-0.75						
CPT	3 months – BL	27	-14.48	12.01	-1.21						
**CAPS-5**	**(PP)**		**Change from BL**			**Group Diff**		**Mean Group Diff**	**Equivalence Test**		**Equivalence Bounds**
**Group**	**Time - Baseline**	**N**	**Mean**	**SD**		**Estimate +/- SE**	**p-value**	**Within +/- 5**	**TOST LB**	**TOST UB**	**Within +/- 10**
TCTSY	Midpoint – BL	44	-8.11	9.66	-0.84	-0.82 ± 2.70	.762	YES	-5.33	3.68	YES
TCTSY	2 weeks – BL	44	-11.50	12.55	-0.92	-2.45 ± 3.30	.461	YES	-7.97	3.06	YES
TCTSY	3 months – BL	36	-11.36	11.98	-0.95	4.16 ± 3.34	.218	YES	-1.43	9.76	YES
CPT	Midpoint – BL	24	-7.29	12.27	-0.59						
CPT	2 weeks – BL	20	-9.05	11.52	-0.79						
CPT	3 months – BL	21	-15.52	12.51	-1.24						
**PCL-5**	**(ITT)**		**Change from BL**			**Group Diff**		**Mean Group Diff**	**Equivalence Test**		**Equivalence Bounds**
**Group**	**Time - Baseline**	**N**	**Mean**	**SD**		**Estimate +/- SE**	**p-value**	**Within +/- 5**	**TOST LB**	**TOST UB**	**Within +/- 10**
TCTSY	Midpoint – BL	59	-5.49	13.83	-0.40	-4.12 ± 3.17	.197	YES	-9.39	1.15	YES
TCTSY	2 weeks – BL	50	-8.64	14.93	-0.58	0.47 ± 3.64	.897	YES	-5.60	6.54	YES
TCTSY	3 months – BL	39	-9.95	18.60	-0.53	3.27 ± 4.54	.473	YES	-4.30	10.84	NO
CPT	Midpoint – BL	35	-1.37	16.50	-0.08						
CPT	2 weeks – BL	27	-9.11	15.85	-0.57						

© 2023 Zaccari B et al. *JAMA Network Open.*

CPT	3 months – BL	27	-13.22	17.39	-0.76						
**PCL-5**	**(PP)**		**Change from BL**			**Group Diff**		**Mean Group Diff**	**Equivalence Test**		**Equivalence Bounds**
**Group**	**Time - Baseline**	**N**	**Mean**	**SD**		**Estimate +/- SE**	**p-value**	**Within +/- 5**	**TOST LB**	**TOST UB**	**Within +/- 10**
TCTSY	Midpoint – BL	45	-5.47	15.20	-0.36	-2.06 ± 3.98	.613	YES	-8.67	4.62	YES
TCTSY	2 weeks – BL	45	-7.98	14.66	-0.54	1.12 ± 4.03	.782	YES	-5.61	7.84	YES
TCTSY	3 months – BL	36	-10.81	18.45	-0.59	3.62 ± 4.95	.468	YES	-4.66	11.91	NO
CPT	Midpoint – BL	25	-3.44	17.29	-0.20						
CPT	2 weeks – BL	21	-9.10	16.47	-0.55						
CPT	3 months – BL	21	-14.43	17.28	-0.84						

© 2023 Zaccari B et al. *JAMA Network Open.*

eTable 7. Clinical PTSD Diagnostic Changes: PTSD Yes; Positive Response (CAPS-5 Decrease 10+ points); Loss of Diagnosis; and Remission: Percentage of Subjects Within Each Group and Time Point

eTable 7: Clinical PTSD Diagnostic Changes: PTSD Yes; Positive Response (CAPS-5 Decrease 10+ points); Loss of Diagnosis; and Remission: Percentage of Subjects Within Each Group and Time Point

Clinical Outcome		Midpoint		2 weeks post		3 months post	
		TCTSY (n=59)	CPT (n=35)	TCTSY (n=50)	CPT (n=27)	TCTSY (n=40)	CPT (n=27)
**PTSD Yes**	**N (%)**	35 (59%)	25 (71%)	25 (50%)	16 (59%)	20 (50%)	12 (56%)
	**OR 95% CI[1]**	1.714 (0.698, 4.211)		1.455 (0.564, 3.749)		1.250 (0.469, 3.331)	
**Response[3,4]**	**N (%)**	27 (47%)	13 (38%)	25 (51%)	12 (46%)	22 (56%)	17 (63%)
	**OR 95% CI[1]**	0.711 (0.300, 1.685)		0.823 (0.317, 2.134)		1.314 (0.481, 3.590)	
**Loss of Diagnosis[3,4]**	**N (%)**	16 (28%)	8 (24%)	16 (33%)	7 (27%)	15 (39%)	8 (30%)
	**OR 95% CI[1]**	0.808 (0.303, 2.151)		0.760 (0.265, 2.177)		0.674 (0.236, 1.922)	
**Remission[3,4]**	**N (%)**	2 (3%)	5 (15%)	8 (16%)	5 (19%)	6 (15%)	7 (26%)
	**OR 95% CI[1]**	ned[2]		ned[2]		1.925 (0.566, 6.545)	

[1] TCTSY is the reference group

[2] ned = not enough data, more than 20% of cell had expected counts < 5

[3] one subject did not have CAPS data: sample sizes are TCTSY n=58, 49, 39; CPT n=34, 26, 27 (at midpoint, 2wk, 3m)

[4] Response defined as reduction of CAPS-5 severity scores ≥ 10 points; Loss of diagnosis defined as ≥10 point improvement in CAPS-5 and no PTSD diagnosis and CAPS-5 severity score <25); Remission defined as loss of diagnosis plus CAPS-5 score < 12.

© 2023 Zaccari B et al. *JAMA Network Open.*
