# Supplementary material for: Yoga vs Cognitive Processing Therapy for Military Sexual Trauma–Related Posttraumatic Stress Disorder: A Randomized Clinical Trial
Source: JAMA Netw Open. 2023 Dec 8;6(12):e2344862. doi: 10.1001/jamanetworkopen.2023.44862 (PMC10709771; doi:10.1001/jamanetworkopen.2023.44862)
Supplement: Supplement 3. — Data Sharing Statement [file jamanetwopen-e2344862-s003.pdf]

Data Sharing Statement

Zaccari. Yoga vs Cognitive Processing Therapy for Military Sexual Trauma–Related Posttraumatic Stress Disorder. *JAMA Netw Open*. Published December 08, 2023. doi:10.1001/jamanetworkopen.2023.44862

Data
Data available: No

Additional Information
Explanation for why data not available: Additional Information Explanation for why data not available: Data are available to VA investigators who obtain a signed data use agreement from the PI. Because the study was approved and initiated before VA implemented its current data sharing policies, the consent form did not include consent language for open data sharing. We are glad to accommodate requests for data by collaboration on secondary analyses when feasible.
